# Supplementary material for: Explainable AI unravels sepsis heterogeneity via coagulation-inflammation profiles for prognosis and stratification
Source: Nat Commun. 2025 Nov 24;16:10396. doi: 10.1038/s41467-025-65365-z (PMC12644763; doi:10.1038/s41467-025-65365-z)
Supplement: Supplementary file 1 — Supplementary Information [file 41467_2025_65365_MOESM1_ESM.pdf]

Supplementary Information for

Explainable AI Unravels Sepsis Heterogeneity via Coagulation-Inflammation Profiles for Prognosis and Stratification

Table of Contents

Supplementary Figure 1. Integrated framework: An overview of models, methods, biomarkers, and clinical applications in our study 1

Supplementary Figure 2. Research framework for cluster and subphenotype. 2

Supplementary Figure 3. Chord diagrams and radar plots showing abnormal clinical variables of clusters obtained by different unsupervised methods 3

Supplementary Figure 4. Correlation analysis diagram 4

Supplementary Figure 5. Hyperparameter sensitivity analysis of the number of Transformer layers and attention heads on model prognostic performance 5

Supplementary Figure 6. Identification of genes as diagnostic biomarkers 6

Supplementary Figure 7. Identification of genes as prognostic biomarkers 7

Supplementary Figure 8. The prognostic and diagnostic efficacy of STAT5B, MTHFR, HPSE, AAK1 and MX1 in external cohort 9

Supplementary Figure 9. The diagnostic efficacy of CD59, SERPINB2, CFD, and P2RX1 in external cohorts 9

Supplementary Figure 10. Bin visualization of coagulation-inflammatory markers with scorecard 10

Supplementary Figure 11. Potential benefits of heparin in sepsis management 11

Supplementary Figure 12. Flowchart depicting the cohort selection process 12

Supplementary Figure 13. Schematic illustration of the working principle of SMART .. 13

Supplementary Table 1. Prognostic prediction performance of models 14

Supplementary Table 2. Baseline characteristics of the MIMIC-III/IV 15

Supplementary Table 3. Baseline characteristics of the eICU-CRD and Local ICU 18

Supplementary Table 4. Assessment of optimal cluster number for 36 sepsis predictors 19

Supplementary Table 5. Performance comparison of the pre-trained SepsisFormer and baseline models on the internal test set 20

Supplementary Table 6. Performance comparison of the fine-tuned SepsisFormer and baseline models on the two external validation cohorts 21

Supplementary Table 7. Comparison the of domain adaptation methods 22

Supplementary Table 8. Different roles of the prognostic genes 23

Supplementary Table 9. Different roles of the diagnostic genes 24

Supplementary Table 10. Cohen's Kappa agreement metrics for survival prediction 25

Supplementary Table 11. The optimal number of clusters for subphenotype 26

Supplementary Table 12. Clinical characteristics between subphenotypes 27

Supplementary Table 13. SIRI values of the four study cohorts 28

Supplementary Table 14. HTEs of heparin across subphenotype and risk level 29

Supplementary Table 15. Hyperparameters of SepsisFormer. 30

Supplementary Table 16. Primer sequences for real-time polymerase chain reaction. 31

Supplementary Method 1. Detailed algorithm derivation of SepsisFormer. 32
Supplementary Method 2. DEGs screening steps and RT-qPCR experimental steps....... 40
Supplementary Method 3. External validation of diagnostic performance of the DGEs . 41
Supplementary Method 4. External validation of prognostic performance of the DGEs. 41
Supplementary Method 5. Detailed algorithm derivation of SMART.............................. 42
Supplementary Method 6. Pseudocode of discretization based on ChiMerge................. 44
Supplementary Method 7. Pseudocode of adjusting bins 45
Supplementary case material. The external validation cohort of 40 cases of SMART 46

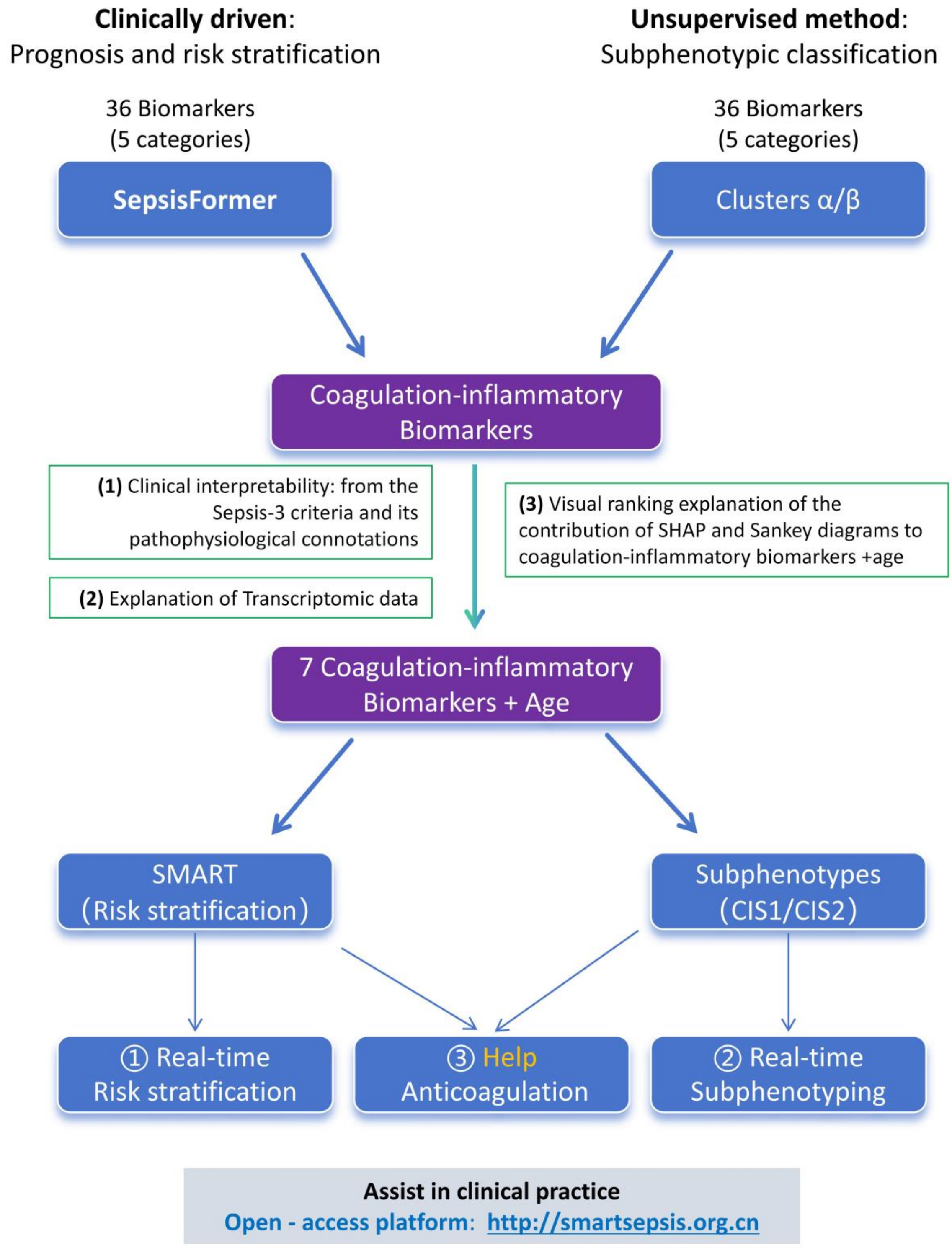

Supplementary Figure 1. Integrated framework: An overview of models, methods, biomarkers, and clinical applications in our study.

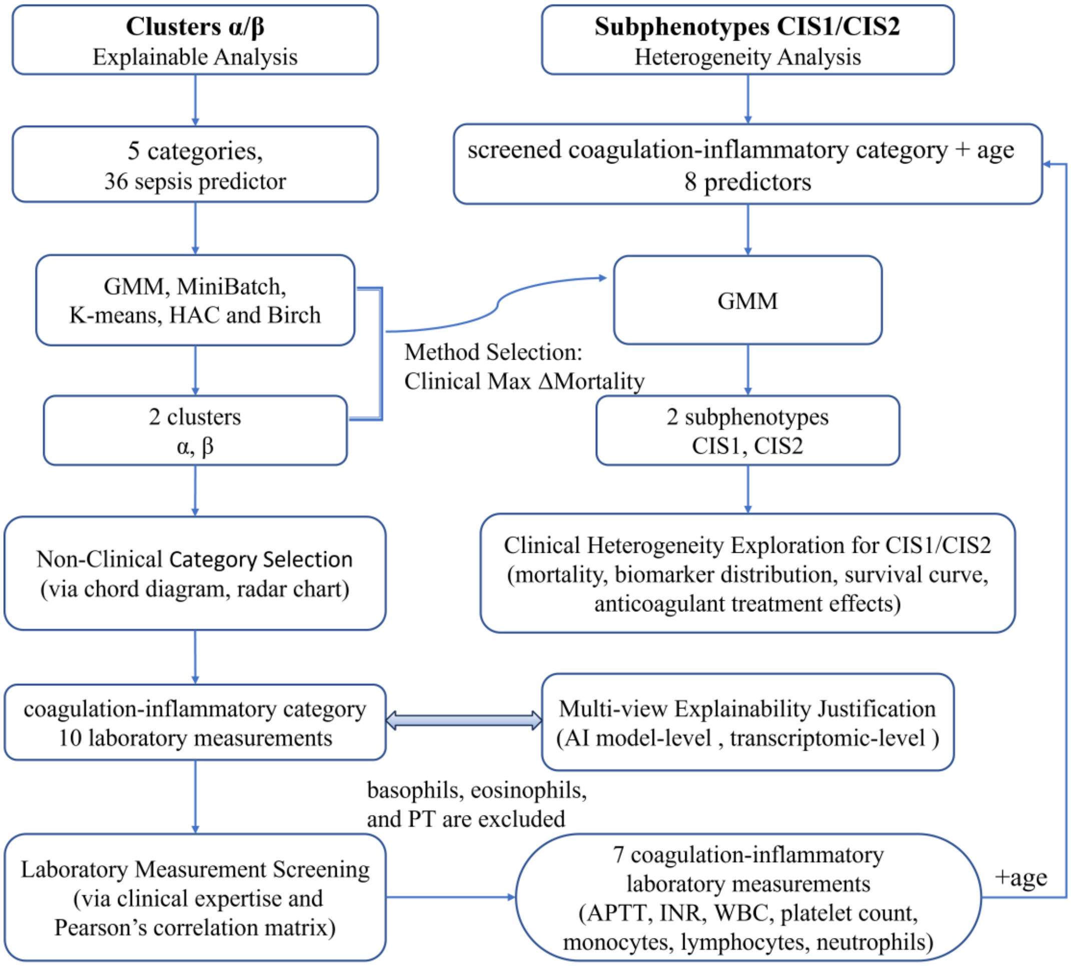

Supplementary Figure 2. Research framework for cluster and subphenotype.

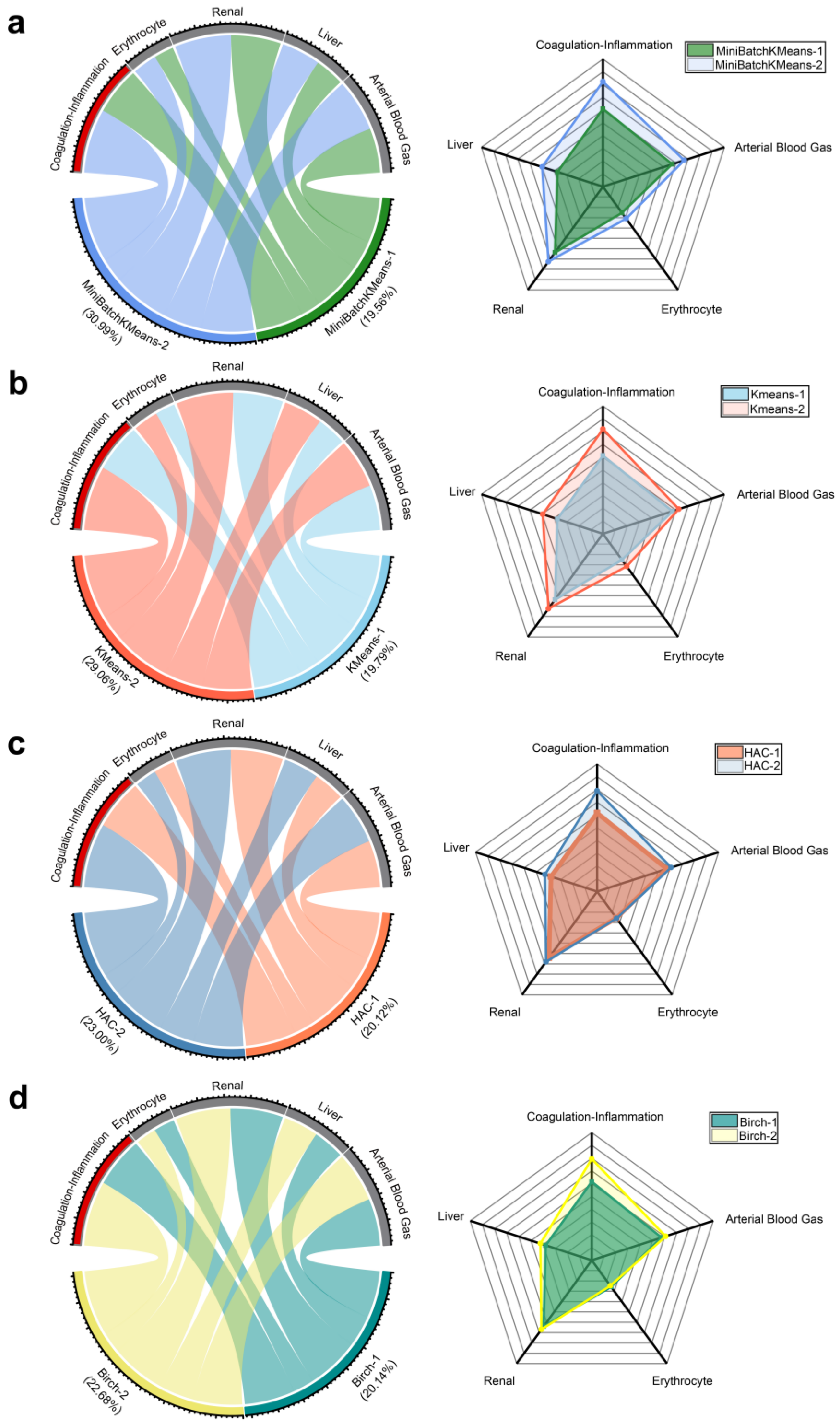

Supplementary Figure 3. Chord diagrams and radar plots showing abnormal clinical variables of clusters obtained by different unsupervised methods. a, MiniBatchKMeans. **b**, K-means. **c**, Hierarchical Agglomerative Clustering (HAC). **d**, Balanced Iterative Reducing and Clustering using Hierarchies (Birch).

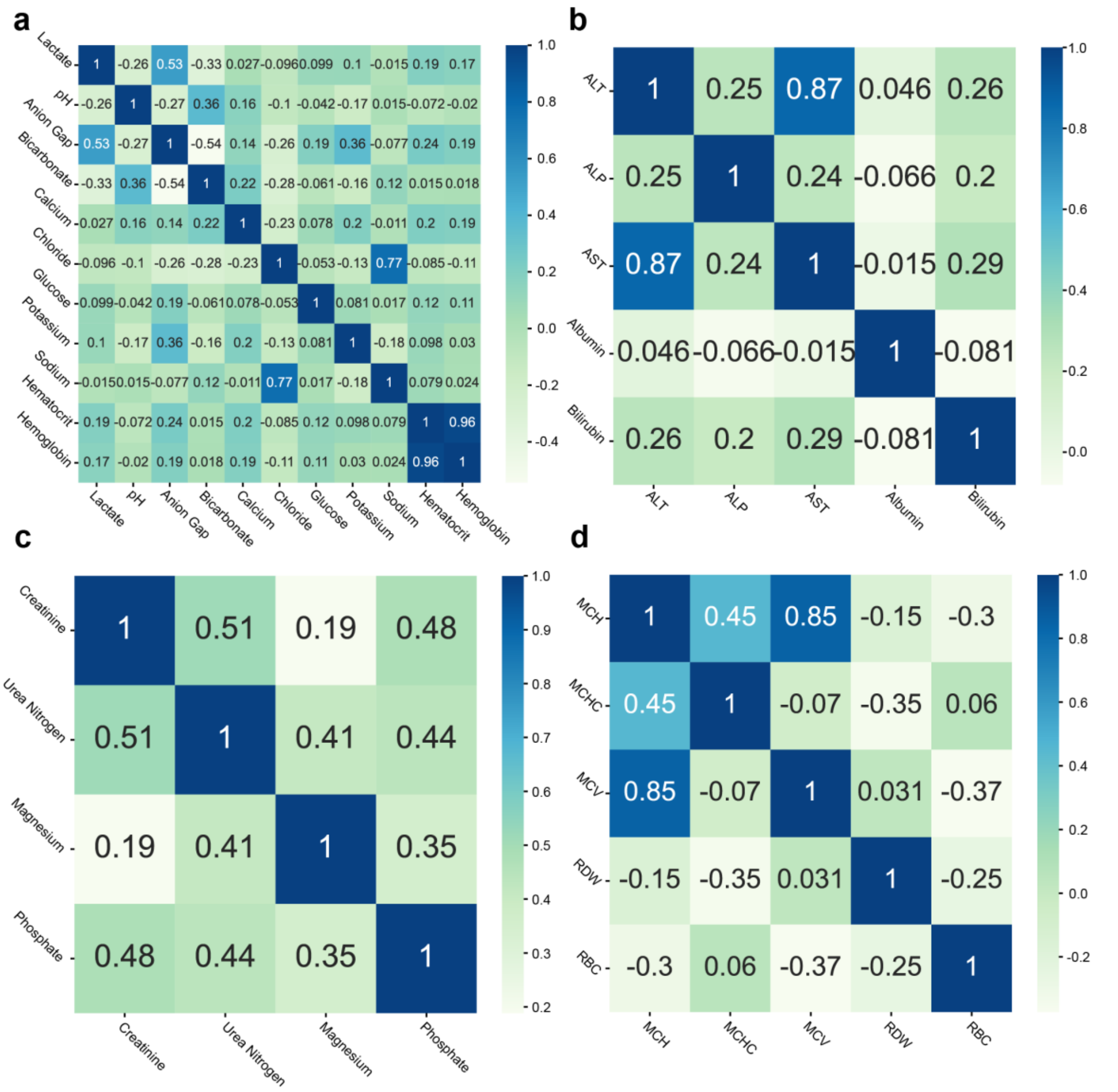

Supplementary Figure 4. Correlation analysis diagram. **a**, Correlation analysis of arterial blood gas markers. **b**, Correlation analysis of liver markers. **c**, Correlation analysis of renal markers. **d**, Correlation analysis of erythrocyte markers.

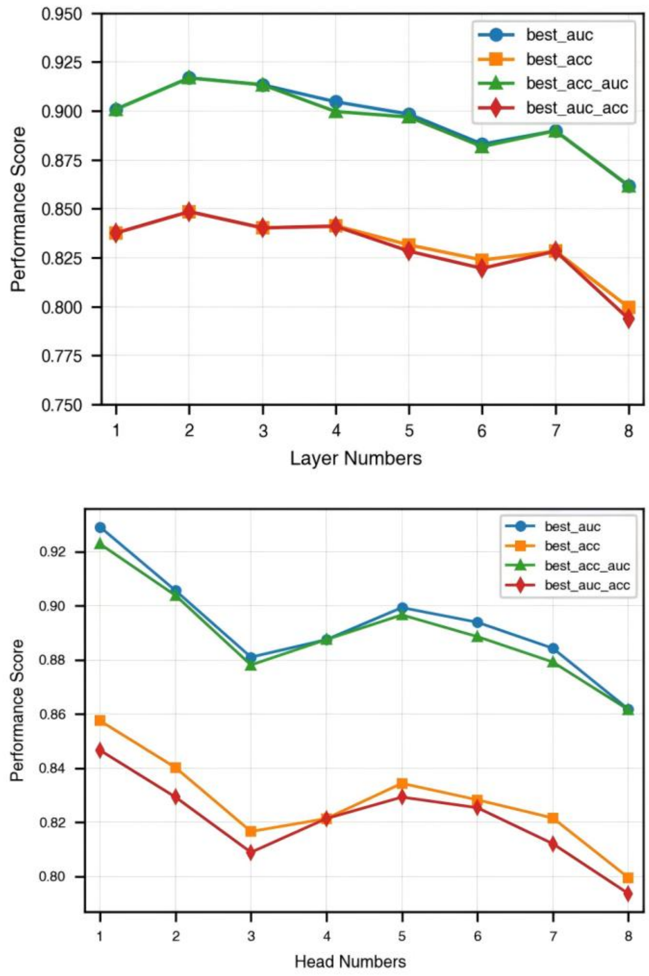

Supplementary Figure 5. Hyperparameter sensitivity analysis of the number of Transformer layers and attention heads on model prognostic performance.

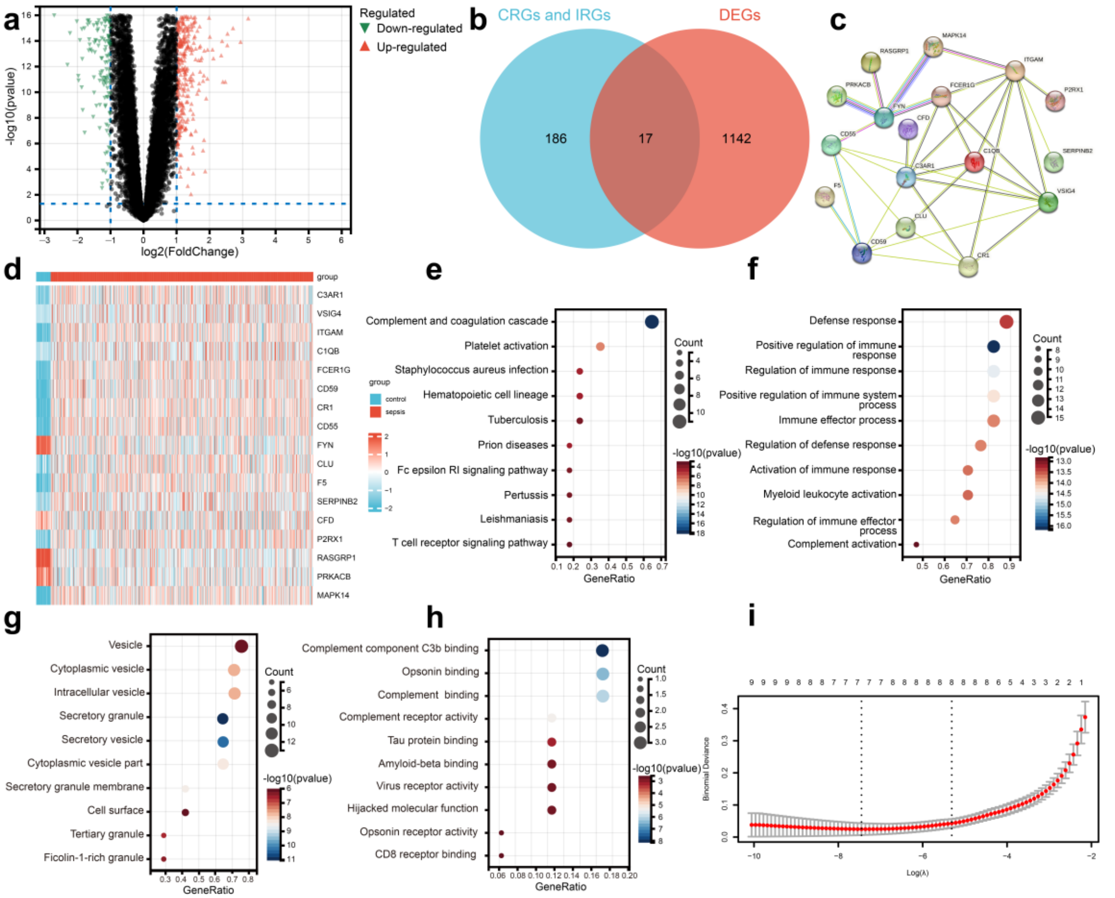

Supplementary Figure 6. Identification of genes as diagnostic biomarkers. a, Volcano plot generated to display the DEGs between sepsis patients and healthy controls. **b,** Venn diagram presenting the overlapping genes between CRGs, IRGs, and DEGs. **c,** The PPI network of intersection genes, applying a filter condition (combined score ＞ 0.4). **d,** The heat map displaying the 17 DECRGs and DEIRGs. **e-h**, Pathway enrichment analysis shows the results of KEGG (**e**), GO biological pathway (**f**), GO cell (**g**), and GO molecular function (**h**) in 17 DECRGs and DEIRGs. *P*-value < 0.05 and FDR < 0.1 were considered as statistically significant thresholds. **i,** Identification of 7 DECRGs and DEIRGs using Lasso regression.

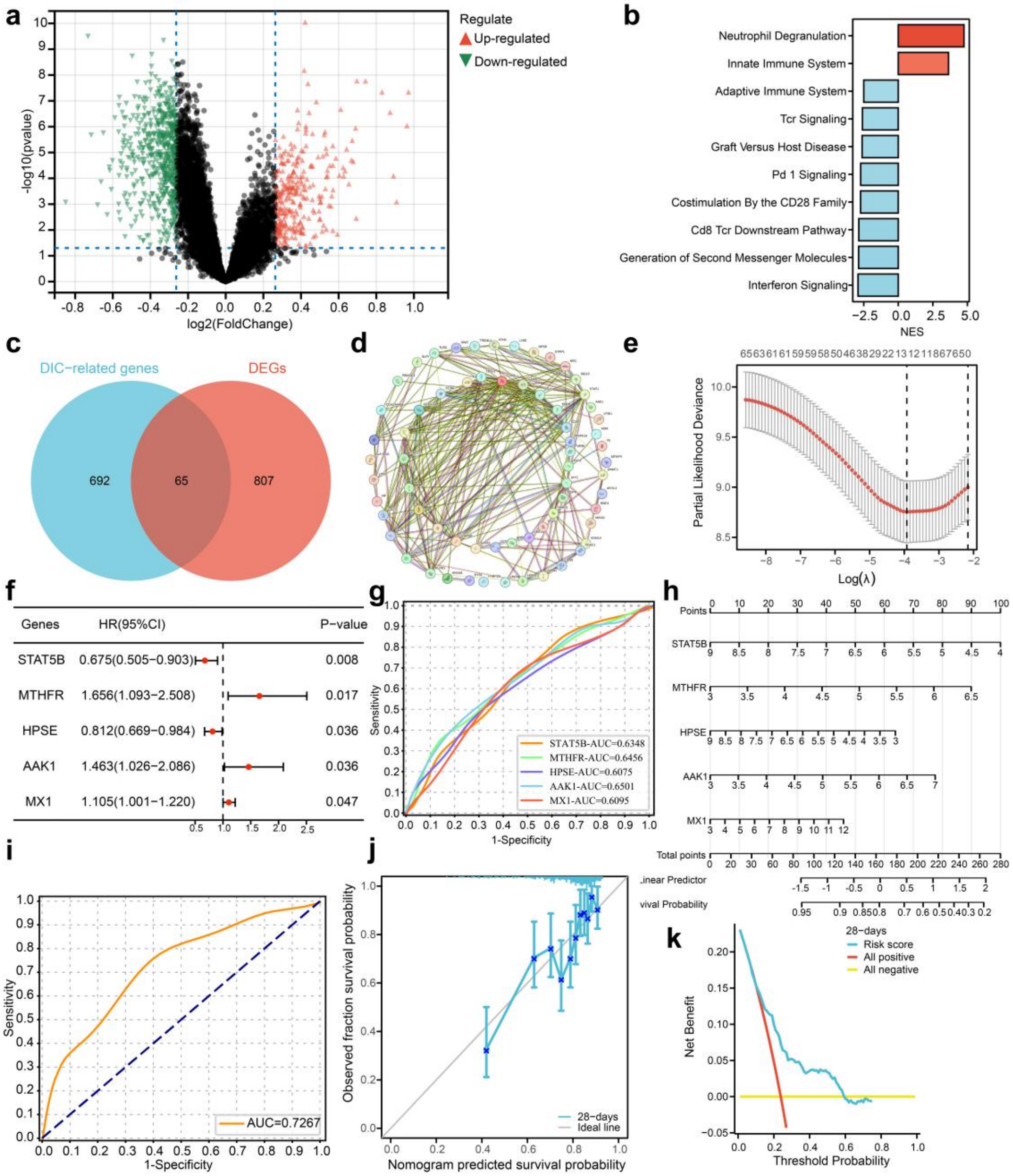

Supplementary Figure 7. Identification of genes as prognostic biomarkers. a, Volcano plot showing DEGs between septic survivors and non-survivors. The threshold for DEGs was set at $p < 0.05$ and fold change > 1.2. **b,** The top ten GSEA enrichment analyses of DEGs and the adjusted $p < 0.05$ and FDR < 0.25 were considered statistically significant. The blue represents down-regulation in sepsis survivors, while red represents up-regulation. **c,** Venn diagram illustrating the overlapping genes between DIC-related genes and DEGs. **d,** The PPI network of intersection genes, applying a filter condition (combined score > 0.4). **e,** Identification of 12 potential DIC-related DEGs using Lasso regression. **f,** Forest plot of prognostic-related DEGs based on stepwise multivariate Cox regression analysis ($p < 0.1$).

g, ROC curves generated to evaluate the predictive value of each biomarker. **h,** A nomogram to predict the 28-day survival probability of sepsis patients. **i,** ROC curves (AUC = 0.7267) of the nomogram model in sepsis. **j,** Calibration curve evaluating the predictive ability of the nomogram model. **k,** DCA curve assessing the clinical value of the nomogram model.

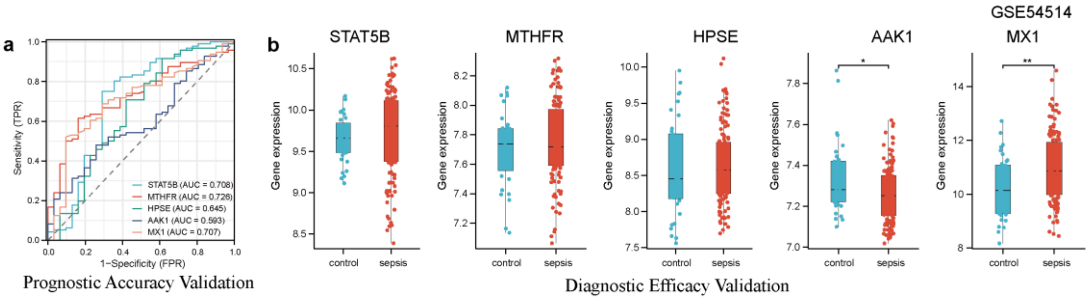

Supplementary Figure 8. The prognostic and diagnostic efficacy of STAT5B, MTHFR, HPSE, AAK1 and MX1 in external cohort. From GSE54514. a. prognostic AUC curves; b. Expression levels of STAT5B, MTHFR, HPSE, AAK1 and MX1. *$p < 0.05$, ***$p < 0.001$ vs. Healthy control.

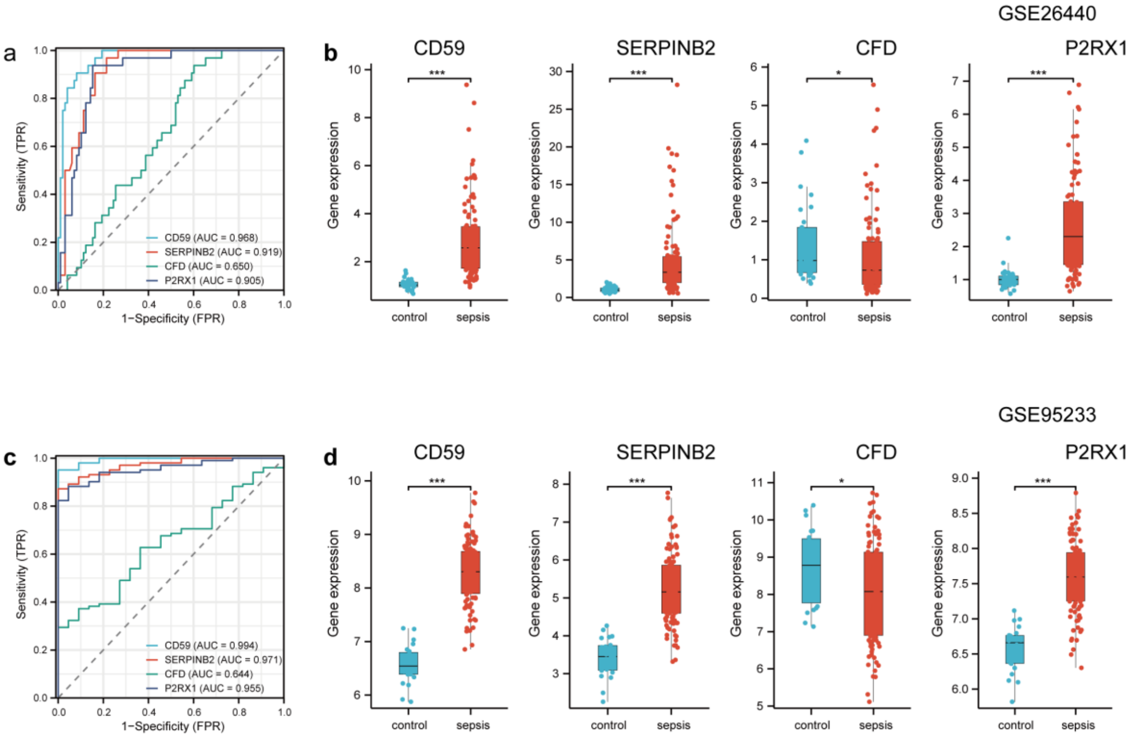

Supplementary Figure 9. The diagnostic efficacy of CD59, SERPINB2, CFD, and P2RX1 in external cohorts. a. and c., AUC curves; b. and d., Expression levels of CD59, SERPINB2, CFD, and P2RX1. *$p < 0.05$, *** $p < 0.001$.

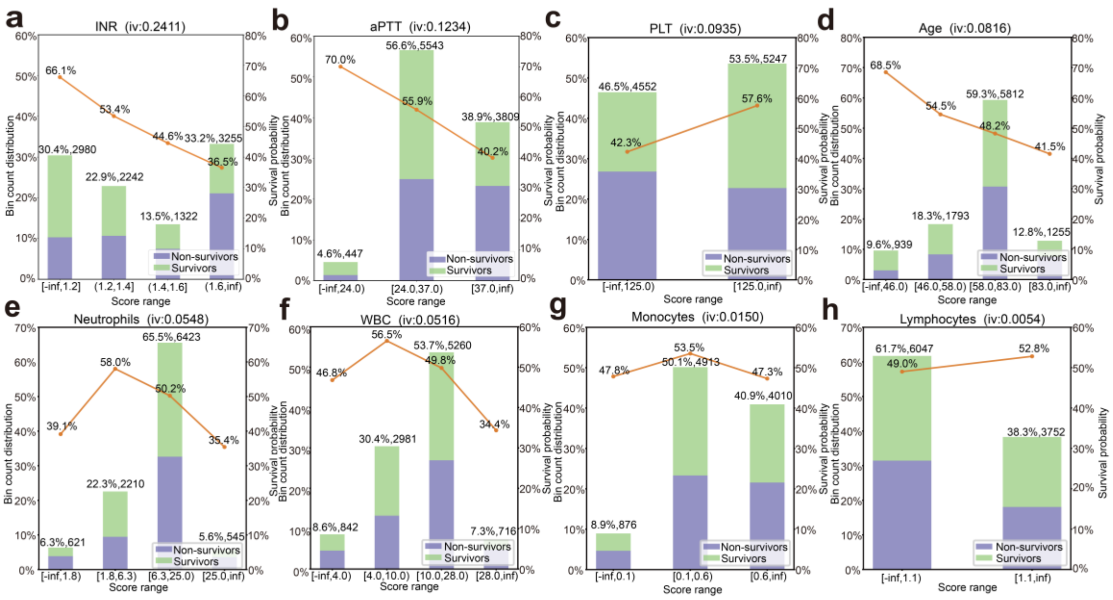

Supplementary Figure 10. Bin visualization of coagulation-inflammatory markers with scorecard. a-h, For non-discrete data within routine laboratory measurements, this study utilized chi-square binning to convert them into discrete data, aiming to enhance the sepsis predictors' capabilities of the model by fully extracting their latent information content. WOE (Weight of Evidence) was employed to describe the relationship between various routine laboratory measurements and patient prognosis, while IV (Information Value) reflected the strength of this relationship. It can be observed that INR and APTT both have IV values greater than 0.1, indicating their strong predictive power. In contrast, Platelet Count, Age, Neutrophils, and WBC have IV values ranging from 0.02 to 0.1, signifying moderate predictive capabilities. On the other hand, Lymphocytes and Monocytes exhibit IV values below 0.02, suggesting weak predictive abilities regarding patient prognosis.

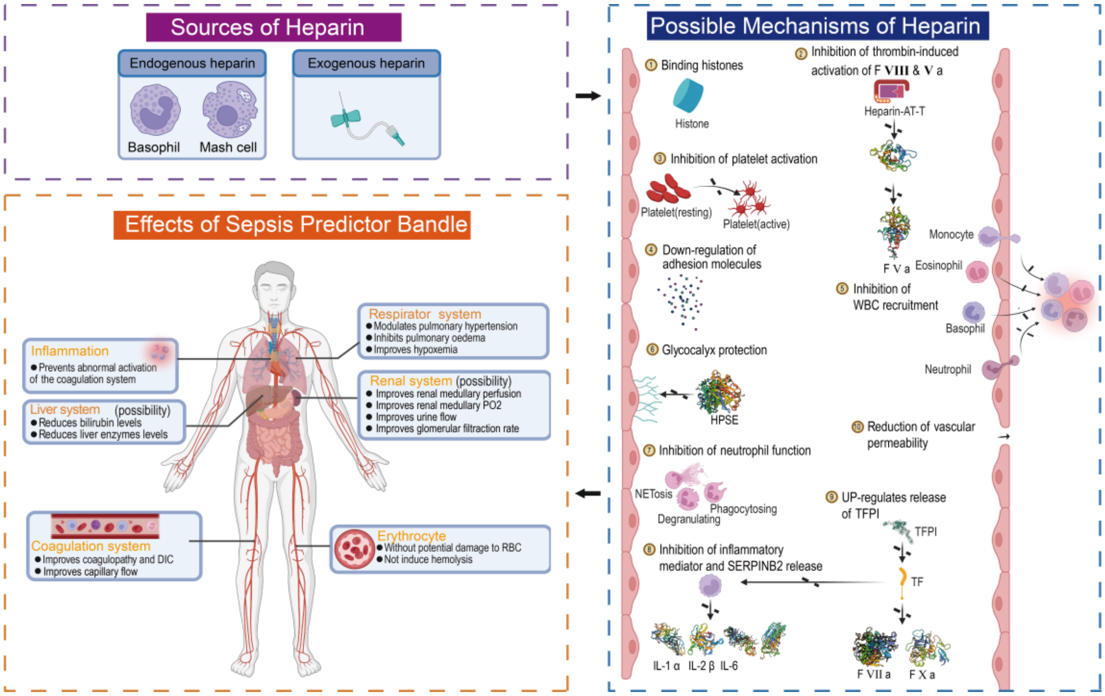

Supplementary Figure 11. Potential benefits of heparin in sepsis management. Created in BioRender. Niu, B. (2025) https://BioRender.com/uioeb32.

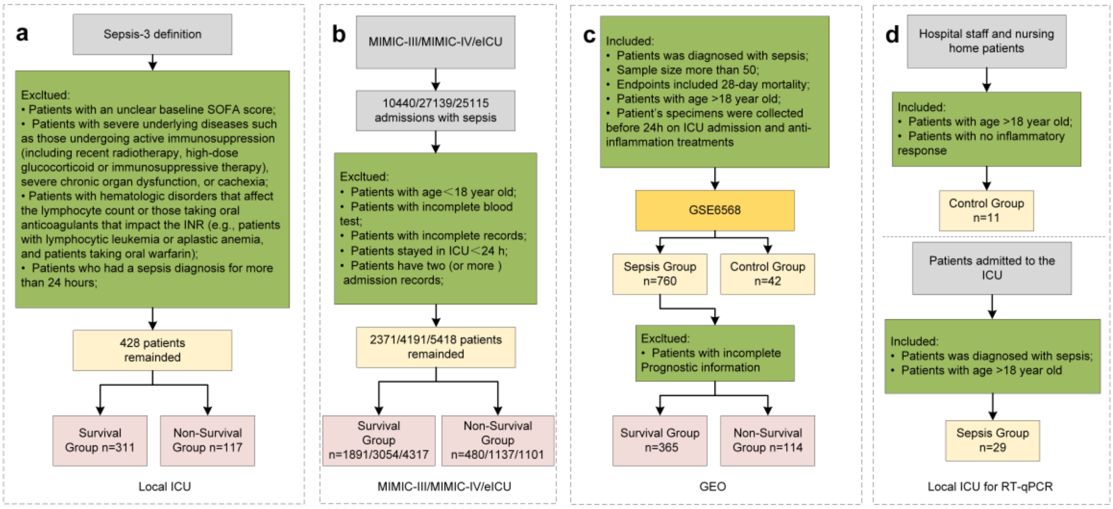

Supplementary Figure 12. Flowchart depicting the cohort selection process. a, Routine measurement cohort acquisition process for sepsis in local hospitals. **b,** Electronic health record dataset acquisition process for sepsis. **c,** The process of obtaining mRNA expression profiles for DIC-related genes in the database. **d,** the process of obtaining the mRNA expression profiling of genes from a local hospital.

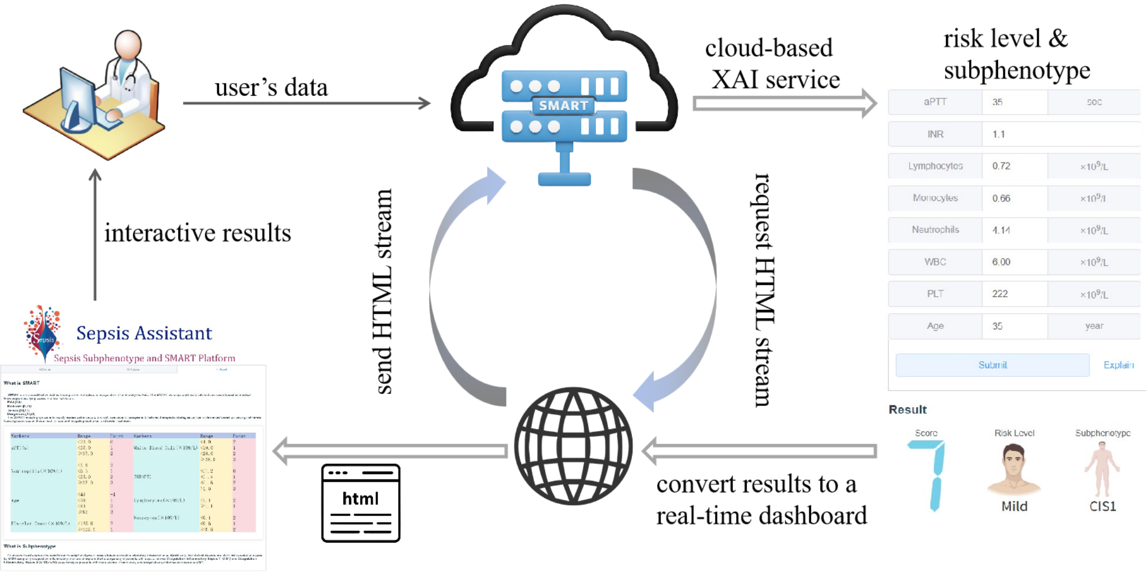

Supplementary Figure 13. Schematic illustration of the working principle of SMART. When the clinician accesses the sepsis subphenotype and SMART platform, they first enter the system's project address correctly into the web browser. The browser then sends a page request to the server, which locates the corresponding system files based on the project address provided. The server converts the retrieved files into an HTML data stream and returns them to the front-end browser for display. Finally, the clinician inputs the patient's clinical information into the system, which automatically computes and returns three results: the score, risk level, and subphenotype. Created in BioRender. Niu, B. (2025) https://BioRender.com/gfidyqi.

Supplementary Table 1. Prognostic prediction performance of models. Using 36 sepsis predictors.

Model	MIMIC-III					MIMIC-IV				
	AUC	ACC	Specificity	Sensitivity	F1	AUC	ACC	Specificity	Sensitivity	F1
LR	0.7895	0.7203	0.7123	0.7281	0.7256	0.7956	0.7104	0.6947	0.7324	0.7247
SGD	0.7761	0.7107	0.7211	0.7005	0.7109	0.7853	0.7123	0.6852	0.7380	0.7250
SVM	0.8408	0.7666	0.7666	0.7504	0.7656	0.8539	0.7791	0.7846	0.7738	0.7826
DT	0.8990	0.8287	0.8011	0.8554	0.8353	0.8946	0.8228	0.7763	0.8667	0.8340
RF	0.9067	0.8540	0.8211	0.8554	0.8519	0.8964	0.8395	0.8172	0.8511	0.8449
LSTM	0.9111	0.8584	0.7833	0.9312	0.8698	0.9026	0.8470	0.8248	0.8679	0.8535
GRU	0.9109	0.8645	0.8135	0.9139	0.8726	0.9018	0.8579	0.8178	0.8959	0.8663
GPT_1	0.9183	0.8829	0.8252	0.9208	0.8847	0.9068	0.8680	0.8533	0.9015	0.8736
SepsisFormer	0.9301	0.8837	0.8312	0.9346	0.8909	0.9178	0.8705	0.8556	0.9158	0.8760

Supplementary Table 2. Baseline characteristics of the MIMIC-III/IV.

MIMIC-III<br>MIMIC-IV<br>Median (Min-Max)				
	Non-survivors<br>(N=480)<br>(N=1137)	Survivors<br>(N=1891)<br>(N=3054)	All Data<br>(N=2371)<br>(N=4191)	*P* value
Age	69(61-80)<br>68(58-78)	64(54-74)<br>64(53-75)	65(55-76)<br>65(54-76)	<0.001<br><0.001
Arterial Blood Gas				
Lactate	2.90(1.80-5.00)<br>2.20(1.50-3.60)	2.20(1.50-3.30)<br>1.70(1.20-2.50)	2.30(1.50-3.60)<br>1.80(1.30-2.80)	<0.001<br><0.001
pH	7.34(7.25-7.39)<br>7.35(7.28-7.42)	7.34(7.32-7.41)<br>7.38(7.33-7.44)	7.34(7.30-7.41)<br>7.38(7.31-7.43)	<0.001<br><0.001
Anion Gap	18(15-21)<br>16(13-19)	16(14-19)<br>14(12-17)	17(14-20)<br>14(12-17)	<0.001<br><0.001
Bicarbonate	22(18-26)<br>21(18-25)	23(20-27)<br>24(21-27)	23(20-27)<br>23(20-27)	<0.001<br><0.001
Calcium	8.1(7.4-8.8)<br>8.1(7.6-8.7)	8.3(7.7-9.0)<br>8.2(7.7-8.7)	8.3(7.6-9.0)<br>8.2(7.7-8.7)	<0.001<br><0.05
Chloride	102(97-107.25)<br>103(98-108)	101(97-106)<br>104(99-108)	101(97-106)<br>103(99-108)	0.093<br><0.05
Glucose	129(98-175)<br>125(96-163)	129(103-179)<br>120(98-153)	129(101-178)<br>121(98-156)	0.077<br>0.091
Potassium	4.50(3.90-5.20)<br>4.10(3.70-4.70)	4.30(3.80-4.90)<br>4.00(3.60-4.40)	4.30(3.80-4.90)<br>4.00(3.70-4.40)	<0.001<br><0.001
Sodium	138(133-142)<br>138(134-142)	138(134-141)<br>139(135-142)	138(134-141)<br>138(135-142)	0.366<br><0.01
Hematocrit	32.30(28.88-37.75)<br>29.30(25.50-34.00)	33.60(29.90-37.70)<br>28.60(25.30-32.70)	33.40(29.60-37.70)<br>28.70(25.30-33.10)	<0.05<br><0.001
Hemoglobin	10.5(9.3-12.3)<br>9.5(8.3-11.0)	11.0(9.7-12.6)<br>9.2(8.1-10.7)	11.0(9.6-12.6)<br>9.3(8.2-10.8)	<0.001<br><0.01
Renal				
Creatinine	1.90(1.20-3.00)<br>1.50(0.90-2.60)	1.40(1.00-2.60)<br>1.10(0.70-1.80)	1.50(1.00-2.70)<br>1.20(0.80-2.00)	<0.001<br><0.001
Urea Nitrogen	40.00(26.00-60.25)<br>34.00(20.00-56.00)	29.00(19.00-48.00)<br>24.00(15.00-40.00)	31.00(20.00-50.00)<br>26.00(16.00-45.00)	<0.001<br><0.001
Magnesium	1.90(1.60-2.30)<br>2.00(1.80-2.30)	1.80(1.60-2.10)<br>2.00(1.80-2.20)	1.80(1.60-2.10)<br>2.00(1.80-2.20)	<0.01<br><0.001
Phosphate	4.00(3.20-5.30)<br>3.80(2.90-4.90)	3.40(2.60-4.30)<br>3.30(2.60-4.00)	3.50(2.70-4.50)<br>3.40(2.70-4.20)	<0.001<br><0.001
Coagulation-inflammation				

PT	15.70(13.90-20.82)<br>16.30(13.60-21.50)	14.40(13.20-17.05)<br>14.00(13.00-17.70)	14.60(13.20-17.75)<br>15.00(13.10-18.80)	<0.001<br><0.001
aPTT	33.80(28.30-42.38)<br>37.30(30.20-49.30)	30.60(26.70-36.60)<br>33.10(28.80-41.78)	31.10(27.00-37.60)<br>34.00(29.10-43.90)	<0.001<br><0.001
INR	1.50(1.27-2.20)<br>1.50(1.20-2.00)	1.30(1.10-1.60)<br>1.30(1.20-1.60)	1.30(1.20-1.70)<br>1.40(1.20-1.70)	<0.001<br><0.001
Platelet count	214.5(133.75-330.25)<br>155.0(86.00-247.00)	233.0(156-332)<br>200.5(119-301)	230(152-331)<br>189(110-286.5)	<0.05<br><0.001
Lymphocytes	0.86(0.44-1.45)<br>0.83(0.42-1.47)	0.95(0.53-1.53)<br>0.86(0.47-1.47)	0.92(0.52-1.52)<br>0.85(0.46-1.47)	<0.05<br>0.086
Monocytes	0.44(0.23-0.76)<br>0.55(0.27-0.98)	0.41(0.23-0.67)<br>0.53(0.29-0.89)	0.42(0.23-0.69)<br>0.54(0.28-0.92)	0.064<br>0.231
Neutrophils	11.18(6.47-17.02)<br>10.13(5.62-15.77)	9.30(5.84-14.12)<br>8.56(5.39-12.73)	9.64(5.92-14.64)<br>8.84(5.44-13.58)	<0.001<br><0.001
WBC	13.80(8.67-20.23)<br>12.60(7.70-19.00)	11.7(7.7-17.0)<br>10.8(7.2-15.8)	12.2(7.8-17.6)<br>11.2(7.3-16.6)	<0.001<br><0.001
Basophils	0.0(0.0-0.2)<br>0.1(0.0-0.3)	0.1(0.0-0.3)<br>0.2(0.0-0.4)	0.1(0.0-0.3)<br>0.1(0.0-0.3)	<0.001<br><0.001
Eosinophils	0.1(0.0-0.7)<br>0.2(0.0-1.0)	0.4(0.0-1.3)<br>0.5(0.0-1.8)	0.3(0.0-1.0)<br>0.4(0.0-1.6)	<0.001<br><0.001
Liver				
ALT	27(15-54)<br>29(17-64)	25(15-52)<br>27(15-55)	26(15-52)<br>27(16-58)	0.147<br><0.001
ALP	123(85-190)<br>109(75-176)	107(75-152)<br>101(71-169)	110(76-159)<br>104(72-170)	<0.001<br><0.01
AST	41.00(24.00-92.75)<br>52.00(27.00-112.00)	33.00(21.00-68.00)<br>36.00(22.00-70.00)	34.00(21.00-72.50)<br>39.00(23.00-79.00)	<0.001<br><0.001
Albumin	2.7(2.2-3.0)<br>2.6(2.2-3.1)	2.9(2.6-3.4)<br>2.8(2.4-3.2)	2.9(2.5-3.3)<br>2.7(2.3-3.2)	<0.001<br><0.001
Bilirubin	0.7(0.4-1.5)<br>0.9(0.5-3.1)	0.6(0.3-1.1)<br>0.7(0.4-1.5)	0.6(0.3-1.2)<br>0.7(0.4-1.8)	<0.001<br><0.001
Erythrocyte				
MCH	30.10(28.10-31.70)<br>30.30(28.60-32.00)	30.00(28.50-31.70)<br>29.85(28.30-31.30)	30.00(28.40-31.70)<br>30.00(28.40-31.50)	0.670<br><0.001
MCHC	32.60(31.40-33.60)	33.10(32.00-34.20)<br>32.40(31.40-33.50)	33.00(31.90-34.10)<br>32.40(31.30-33.50)	<0.001<br>0.191

	32.40(31.20-33.50)			
MCV	92(86-97)<br>94(88-99)	91(86-95)<br>92(87-96)	91(86-96)<br>92(88-97)	<0.01<br><0.001
RDW	16.35(14.90-18.02)<br>16.70(15.10-18.70)	15.40(14.20-17.00)<br>15.90(14.50-17.80)	15.70(14.25-17.30)<br>16.10(14.60-18.10)	<0.001<br><0.001
RBC	3.58(3.10-4.10)<br>3.17(2.70-3.69)	3.72(3.26-4.21)<br>3.14(2.74-3.63)	3.70(3.23-4.19)<br>3.14(2.73-3.64)	<0.001<br>0.575

Note: Non-parametric two-tailed Mann-Whitney U test was applied to evaluate median differences.

Supplementary Table 3. Baseline characteristics of the eICU-CRD and Local ICU.

eICU-CRD<br>Local ICU<br>Median (Min-Max)				
	Non-survivors<br>(N=1101)<br>(N=117)	Survivors<br>(N=4307)<br>(N=311)	All Data<br>(N=5418)<br>(N=428)	*P* value
aPTT	37.50(32.00-47.30)<br>45.30(38.70-53.70)	34.00(29.60-40.60)<br>40.50(34.50-48.05)	34.70(30.00-42.00)<br>41.65(35.38-49.40)	<0.001<br><0.001
INR	1.50(1.27-2.10)<br>1.41(1.28-1.55)	1.30(1.11-1.60)<br>1.29(1.19-1.43)	1.31(1.20-1.70)<br>1.31(1.23-1.47)	<0.001<br><0.001
Platelet count	170(97-243)<br>134(88-207)	182(121-255)<br>140(89-212)	178(116-254)<br>138(88-212)	<0.001<br>0.954
Lymphocytes	0.76(0.43-1.28)<br>0.51(0.33-0.68)	0.89(0.49-1.42)<br>0.55(0.38-0.73)	0.86(0.48-1.40)<br>0.54(0.37-0.72)	<0.001<br>0.148
Monocytes	0.67(0.32-1.13)<br>0.32(0.16-0.68)	0.74(0.40-1.18)<br>0.49(0.26-0.74)	0.72(0.38-1.17)<br>0.46(0.22-0.72)	<0.001<br><0.05
Neutrophils	11.56(6.47-17.76)<br>9.03(5.71-13.58)	10.92(6.54-16.00)<br>10.51(6.24-15.48)	11.00(6.54-16.29)<br>9.99(6.12-15.05)	0.056<br>0.083
WBC	14.20(8.60-21.24)<br>9.80(6.33-15.43)	13.50(8.60-19.29)<br>11.68(7.22-16.62)	13.60(8.60-19.60)<br>11.02(6.96-16.25)	<0.05<br>0.075
Age	69(58-78)<br>67(53-77)	65(54-76)<br>67(55-75)	66(55-76)<br>67(55-76)	<0.001<br>0.664

Note: Non-parametric two-tailed Mann-Whitney U test is applied to evaluate median differences.

Supplementary Table 4. Assessment of optimal cluster number for 36 sepsis predictors.

Class number	Silhouette score	Davies-Bouldin score	Calinski-Harabasz score
2	0.16	2.38	292.84
3	0.12	4.06	207.35
4	0.06	4.28	155.00
5	0.05	4.16	152.17
6	0.05	4.24	180.07
7	0.07	4.56	161.02
8	0.07	5.52	139.85
9	0.04	5.02	128.32

The Silhouette score, which indicates how similar a sample is to its assigned cluster, ranges from -1 to 1, with higher values (closer to 1) being preferred.

The Davies–Bouldin Score, which quantifies the average similarity between each cluster and its most similar cluster, ranges from zero onward, with lower values (closer to zero) being better.

Calinski–Harabasz score, the ratio between the within-cluster dispersion and the between-cluster dispersion, ranged from zero upward—higher values being better for a dataset.

Supplementary Table 5. Performance comparison of the pre-trained SepsisFormer and baseline models on the internal test set.

Model	AUC	ACC	Specificity	Sensitivity	F1
LR	0.6295	0.5997	0.6033	0.5961	0.5992
SGD	0.6304	0.5981	0.6012	0.5950	0.5979
SVM	0.6513	0.6005	0.5302	0.6705	0.6270
DT	0.7629	0.6975	0.6695	0.7253	0.7065
RF	0.7545	0.6861	0.6498	0.7221	0.6979
LSTM	0.8249	0.7524	0.7261	0.7783	0.7600
GRU	0.8140	0.7804	0.7972	0.8116	0.7879
GPT_1	0.8225	0.7590	0.6636	0.8533	0.7807
SepsisFormer	0.8558	0.8337	0.7398	0.9264	0.8487

Note: The internal test set (*N*= 2,337) was derived from Cohort 1 (*N*= 7,789 septic patients from MIMIC-III and eICU-CRD), following a 70% training and 30% testing split.

Supplementary Table 6. Performance comparison of the fine-tuned SepsisFormer and baseline models on the two external validation cohorts.

Model	MIMIC-IV					Local ICU				
	AUC	ACC	Specificity	Sensitivity	F1	AUC	ACC	Specificity	Sensitivity	F1
LR	0.7173	0.6525	0.6367	0.6674	0.6636	0.7034	0.6471	0.7356	0.5700	0.6333
SGD	0.7211	0.6657	0.6343	0.6954	0.6812	0.7020	0.6257	0.7701	0.5000	0.5882
SVM	0.7147	0.6611	0.5254	0.7895	0.7053	0.7444	0.6952	0.7011	0.6900	0.7077
DT	0.7912	0.7363	0.7078	0.7626	0.7030	0.7760	0.7273	0.7701	0.6900	0.7302
RF	0.8039	0.7560	0.7138	0.7581	0.7615	0.7942	0.7112	0.7931	0.6400	0.7033
LSTM	0.8121	0.7514	0.6852	0.8141	0.7709	0.8007	0.7380	0.6667	0.8000	0.7656
GRU	0.8157	0.7693	0.7172	0.8186	0.7848	0.8336	0.7433	0.7128	0.7742	0.7500
GPT_1	0.7914	0.7405	0.6769	0.8007	0.7602	0.8250	0.7487	0.7340	0.7634	0.7513
SepsisFormer	0.8596	0.8247	0.7037	0.9312	0.8497	0.8364	0.7754	0.7093	0.7940	0.7701

Note: External validation was performed on Cohort 2 (N=4,191 septic patients from MIMIC-IV) and Cohort 3 (N=428 septic patients from a local ICU) using 10-fold cross-validation.

Supplementary Table 7. Comparison the of domain adaptation methods. SepsisFormer in septic patients using coagulation-inflammatory markers and age obtained from the Local ICU cohort under different domain adaptation methods.

Method	AUC	ACC	Specificity	Sensitivity	F1 Score
No-adaptation baseline	0.8364	0.7754	0.7093	0.8317	0.8000
Mean-teacher	0.8014	0.7594	0.8046	0.7200	0.7619
Whitening	0.8091	0.7807	0.8046	0.7600	0.7876
Moment Matching	0.8374	0.7540	0.7558	0.7525	0.7677
MMID-SMOTE	0.8540	0.7914	0.7209	0.8515	0.8152

Supplementary Table 8. Different roles of the prognostic genes.

Genes	Description	Function
STAT5B	Signal transducer and activator of transcription 5B	Involves the JAK/STAT signaling pathway
		Positively regulates hematopoietic differentiation, including B, T, NK, erythroid and megakaryocytic cells
		Endotoxin attenuates growth hormone-induced hepatic insulin-like growth factor I expression by inhibiting JAK2/STAT5 signal transduction and STAT5b DNA binding
		Hyperactivation of STAT5B is associated with the development of various blood malignancies, tumors and a syndrome of severe allergic inflammation
		Mutations in the STAT5B gene are associated with autoimmunity and immunodeficiency
MTHFR	Methylenetetrahydrofolate reductase	Polymorphism of MTHFR is associated with the activation of the coagulation system, disease susceptibility, and sepsis
		Both low and excessive expression can worsen MTX-induced myelosuppression
HPSE	Heparanase	Acts as procoagulant by increasing the generation of activation factor X in the presence of tissue factor and activation factor VII
		Compromises the endothelial glycocalyx
		Affects activities of several types of innate immunocytes
		Promotes inflammation and virus replication to promote cell and tissue damage
		Enhances tumor progression, size, metastasis, and angiogenesis
AAK1	AP2-associated kinase 1	Restrains the coagulation response
		Expresses in mononuclear cells, T lymphocytes, and NK cells, mediating virus endocytosis and promoting inflammatory response
MX1	MX dynamin like GTPase 1	Interferon-induced dynamin-like GTPase with antiviral activity against a wide range of RNA viruses and some DNA viruses

Supplementary Table 9. Different roles of the diagnostic genes.

Genes	Description	Function
CD59	CD59 Molecule	Potent inhibitor of the complement membrane attack complex action
CD59<br>P2RX1	CD59 Molecule<br>Purinergic Receptor P2X 1	Prevents hemolysis
		Correlates with the severity of organ damage assessed by SOFA in septic patients
		Involves c-SRC-related tyrosine phosphorylation of the creatine transporter in skeletal muscle during sepsis
		Enables extracellularly ATP-gated monoatomic cation channel activity
P2RX1<br>CFD	Purinergic Receptor P2X 1<br>Complement Factor D	Promotes neutrophil glycolysis and NETs formation
		Proteases catalyze the cleavage of factor B, the rate-limiting step of the alternative pathway of complement activation
CFD<br>SERPINB2	Complement Factor D<br>Serpin Family B Member 2	Inhibits the activation of complement, neutrophils, and platelets
		Inhibits urokinase-type plasminogen activator
SERPINB2	Serpin Family B Member 2	Appearance in plasma was associated with non-survival of the septic patient
		Regulates immune response in renal injury and aging
		Links to cellular senescence, inflammation, and coagulation

Supplementary Table 10. Cohen's Kappa agreement metrics for survival prediction.

	Kappa	Asymptotic standard error	z	*P* value
Linear	0.266	0.037	6.841	<0.001

Note: Kappa significance via asymptotic normal test
($z = \kappa$ / SE; two-tailed p under H_0:chance agreement).

Supplementary Table 11. The optimal number of clusters for subphenotype.Using 7 coagulation-inflammatory markers and age.

Class number	Silhouette score	Davies-Bouldin score	Calinski-Harabasz score
2	0.52	1.03	2877.16
3	0.48	2.34	1672.61
4	0.30	2.03	1792.53
5	0.28	3.00	1355.92
6	0.27	2.45	1141.57
7	0.23	3.49	992.48
8	0.24	2.77	979.37
9	0.20	2.62	952.58

Silhouette score, a measure of how similar a sample is to its cluster, ranged from -1 to 1—values closer to 1 being better.

Davies–Bouldin Score, a measure of the average similarity of each cluster with its most similar cluster, ranged from zero upward—values closer to zero being better.

Calinski–Harabasz score, the ratio between the within-cluster dispersion and the between-cluster dispersion, ranged from zero upward—higher values being better for a dataset.

Supplementary Table 12. Clinical characteristics between subphenotypes.

MIMIC III<br>MIMIC IV<br>eICU-CRD<br>Local ICU			
Cluster	CIS1 (*N*=1973)<br>CIS1 (*N*=3347)<br>CIS1 (*N*=4360)<br>CIS1 (*N*=366)	CIS2 (*N*=398)<br>CIS2 (*N*=844)<br>CIS2 (*N*=1058)<br>CIS2 (*N*=62)	*P* value
aPTT(s)	30.20(26.60-35.60)<br>32.20(28.40-38.70)<br>33.50(29.20-39.00)<br>40.40(34.50-46.80)	42.50(31.82-71.60)<br>56.15(39.00-76.90)<br>44.45(34.80-64.00)<br>55.80(46.03-68.72)	<0.001<br><0.001<br><0.001<br><0.001
INR	1.30(1.10-1.60)<br>1.30(1.20-1.60)<br>1.30(1.10-1.60)<br>1.30(1.22-1.45)	1.70(1.30-2.50)<br>1.70(1.30-2.40)<br>1.60(1.30-2.50)<br>1.43(1.29-1.72)	<0.001<br><0.001<br><0.001<br><0.001
Lymphocytes<br>($\times 10^9$/L)	0.92(0.52-1.48)<br>0.81(0.43-1.38)<br>0.81(0.46-1.30)<br>0.53(0.37-0.71)	0.94(0.50-1.78)<br>1.09(0.59-1.81)<br>1.11(0.61-1.89)<br>0.61(0.39-0.93)	0.156<br><0.001<br><0.001<br><0.05
Monocytes<br>($\times 10^9$/L)	0.39(0.22-0.62)<br>0.50(0.26-0.83)<br>0.67(0.36-1.07)<br>0.44(0.22-0.70)	0.66(0.28-1.51)<br>0.79(0.39-1.46)<br>1.04(0.55-1.73)<br>0.52(0.24-1.02)	<0.001<br><0.001<br><0.001<br>0.193
Neutrophils<br>($\times 10^9$/L)	9.34(5.82-13.93)<br>8.52(5.30-12.80)<br>10.51(6.32-15.44)<br>10.95(1.15-38.12)	11.47(6.66-20.08)<br>10.79(6.30-16.92)<br>14.05(8.28-21.02)<br>15.73(1.43-71.53)	<0.001<br><0.001<br><0.001<br><0.01
WBC<br>($\times 10^9$/L)	11.70(7.60-16.50)<br>10.70(6.90-15.50)<br>12.72(8.20-18.10)<br>10.69(6.94-15.58)	16.65(9.60-26.65)<br>14.40(8.97-21.80)<br>18.30(11.70-26.16)<br>14.55(8.11-24.41)	<0.001<br><0.001<br><0.001<br><0.01
Platelet count<br>($\times 10^9$/L)	231.00(156.00-331.00)<br>198.00(119.00-300.00)<br>178.00(120.00-250.00)<br>144.00(96.00-218.25)	215.50(130.00-343.75)<br>146.00(83.75-234.00)<br>178.00(101.25-274.00)<br>108.00(48.25-164.25)	0.147<br><0.001<br>0.812<br><0.001
Age	66(58-77)<br>68(58-78)<br>68 (59-78)<br>69(60-77)	58(43-68)<br>53(41-64)<br>51(34-64)<br>46(33-53)	<0.001<br><0.001<br><0.001<br><0.001

Note: Non-parametric two-tailed Mann-Whitney U test was applied to evaluate median differences.

Supplementary Table 13. SIRI values of the four study cohorts.

	MIMIC-III	MIMIC-III	eICU-CRD	Local ICU
Subphenotype				
CIS1	3.52(1.58-7.94)	4.78(1.98-10.37)	7.81(3.38-16.31)	8.14(3.31-16.73)
CIS2	5.81(2.00-26.05)	6.95(2.74-16.77)	11.66(4.60-25.70)	8.77(2.73-24.54)
P value	<0.001	<0.001	<0.001	<0.001
Risk level				
Mild	1.85(1.04-2.99)	2.21(1.22-4.34)	3.36(1.67-6.48)	1.78(1.34-6.72)
Moderate	3.43(1.74-7.48)	4.44(2.07-8.77)	6.61(3.00-12.77)	5.36(2.69-10.73)
Severe	6.63(2.37-16.10)	7.30(2.91-16.06)	11.16(4.91-22.67)	11.31(3.90-22.08)
Dangerous	20.93(2.09-59.26)	26.56(14.65-55.47)	24.58(12.82-44.46)	24.94(16.10-39.92)
P value	<0.001	<0.001	<0.001	<0.001

Note: SIRI= (Neutrophil count × Monocyte count) / Lymphocyte count

Non-parametric two-tailed Mann-Whitney U test was applied to evaluate median differences.

Supplementary Table 14. HTEs of heparin across subphenotype and risk level.

Subgroup	Heparin	Control	ΔR_{H-C}	ΔD_{mean}	ΔD_{median}	P
CIS1_Mild	90.14% (64/71)	93.12% (176/189)	-2.98%	10.76	8.00	0.9992
CIS1_Moderate	80.05% (337/421)	78.66% (1102/1401)	1.39%	11.31	9.00	<0.001
CIS1_Severe	66.38% (152/229)	63.80% (624/978)	2.58%	10.92	8.00	<0.001
CIS1_Dangerous	50.00% (3/6)	46.15% (24/52)	3.85%	1.30	3.00	0.8267
CIS2_Mild	95.65% (22/23)	96.08% (49/51)	-0.43%	4.60	6.00	0.8523
CIS2_Moderate	80.00% (80/100)	75.23% (164/218)	4.77%	15.88	10.50	<0.05
CIS2_Severe	70.33% (64/91)	53.46% (170/318)	16.87%	8.28	9.00	<0.001
CIS2_Dangerous	100.00% (5/5)	47.37% (18/38)	52.63%	12.19	22.50	<0.05

Note: Survival analysis with Log-rank tests to evaluate heparin treatment effects.

Supplementary Table 15. Hyperparameters of SepsisFormer.

Input predictor	36/8
Dense layer1	216/66/48
Dense layer2	36/8
Number of self-attention head	8
Number of Transformer layer head	8
Head dimension	36/8
Batch size	5000
Dropout rate	0.1000
Learning rate	0.0010
Training epoch	1400

Supplementary Table 16. Primer sequences for real-time polymerase chain reaction.

Primer name	Primer sequence (5′ to 3′)
GAPDH-F	CAGGGCTGCTTTTAACTCTGGT
GAPDH-R	GATTTTGGAGGGATCTCGCT
SERPINB2-F	TGATGCGATTTTGCAGGCAC
SERPINB2-R	AAGCTCGCAGACTTCTCACC
P2RX1-F	TCTACGTCATCGGGTGGGT
P2RX1-R	CGTAGTCAGCCACATCCCAG
CFD-F	GACAGCTGCAAGGGTGACTC
CFD-R	GTAGATCCCGGGCTTCTTGC
CD59-F	GCGCCGCCAGGTTCT
CD59-R	GACGGCTGTTTTGCAGTCAG

Supplementary Method 1. Detailed algorithm derivation of SepsisFormer.

A. Domain-adaptive Generator for fine-tuning.

Rationale for Domain Adaptation in Prognostic Modeling. In multi-center Electronic Health Records (EHR) studies, class imbalance within single centers and distribution heterogeneity across multiple clinical sites are prevalent, especially in sepsis prognosis prediction tasks. Existing data augmentation methods, such as the Synthetic Minority Over-sampling Technique (SMOTE), primarily address intra-center class imbalance without considering inter-center distributional shifts. To bridge this gap, we first theoretically justify the necessity of domain adaptation by analyzing class imbalance in mortality outcomes and distribution divergence across clinical centers. These domain discrepancies can degrade model performance when transferring knowledge from one clinical center (source domain) to another (target domain), making domain-adaptive strategies an essential component for robust prognostic modeling.

Class imbalance in sepsis mortality outcomes is a well-established empirical finding. In this study, the statistical chart of eICU, Local ICU, MIMIC III, and MIMIC IV datasets exhibit significant class imbalance between positive samples (non-survivors) and negative samples (survivors), with mortality rates of 25.38%, 37.23%, 25.56%, and 37.62% respectively. The number of non-survivors was significantly lower than that of survivors.

We conducted multi-center covariate distribution shift analyses to clearly demonstrate the significant distribution shifts in laboratory measurements observed across different medical centers. Kolmogorov-Smirnov (KS) non-parametric tests were utilized to assess the consistency of distributions between two medical centers.Results indicate significant distributional shifts (p<0.0001) for the 7 laboratory measurements between every pair of centers across eICU, Local ICU, MIMIC III, and MIMIC IV (see Supplementary Figure for Method). The 7 coagulation-inflammatory laboratory measurements exhibit significant class imbalance within individual centers and notable distribution shifts across different centers. Meanwhile, theses clinical markers also share common feature patterns among these centers, indicating their suitability for domain adaptation approaches to address domain discrepancy (Supplementary Table 2 and 3).

Considering the heterogeneity and commonalities of multi-center laboratory measurements, this paper introduces a method combining domain adaptation and SMOTE to alleviate class imbalance and distribution bias. This ultimately enhances model generalization to unobserved target domains. We assume that the sepsis predictors in source domain (S) and target domain (T) share a common feature space, but their data distributions differ $p(\chi^S) \neq p(\chi^T)$. Domain adaptation aims to reduce this discrepancy by optimizing:

$$\min_\theta \mathcal{L}_S(\theta) + \lambda \times \mathcal{D}(p(\chi^S), p(\chi^T)) \tag{1}$$

where $\mathcal{L}_S(\theta)$ denotes the supervised loss on the source domain, and $\mathcal{D}$ measures the

distribution discrepancy between the source and target domains, with the balancing parameter λ.

Implementation of Existing Domain Adaptation Methods. This study designates a combined detaset of 7,789 septic patients from MIMIC-III and eICU-CRD, Cohort 1, as the source domain. The target domain included two external detasets from previously unobserved centers: Cohort 2 with 4,191 septic patients from MIMIC-IV and the smaller Cohort 3, with 428 septic patients from a local ICU. Each patient's sepsis predictors are derived from the five distinct classification dimensions. To address domain discrepancy in the mortality prediction model, we incorporated several state-of-the-art domain adaptation techniques during model fine-tuning phase.

(1) Mean-Teaching. Employs a teacher-student architecture to enforce prediction consistency under perturbations across domains. (2) Whitening Transformation.Aligns the feature distributions by normalizing covariance structures between source and target domains. (3)Moment Matching: Minimizes the statistical moment differences (e.g., means, variances) between feature representations across domains.Thus, these methods were applied to synthesize domain-adaptive training samples for the fine-tuning phase of the pre-trained sepsis prognosis prediction model.

A Novel Clinically Practical Domain Adaptation Method MMID-SMOTE. Despite the effectiveness of existing domain adaptation approaches, their high computational cost, complex optimization, and resource requirements limit their direct applicability in clinical environments. To overcome this, we propose a novel and lightweight cross-domain data augmentation method, Maximum and Minimum Interval Difference-based SMOTE (MMID-SMOTE).This method integrates statistical moment analysis, domain adaptation, and clinical interpretability constraints into the classic SMOTE framework, ensuring that the synthetic data generated are both statistically valid and clinically meaningful.

Step 1: Statistical Moment Calculation and Distribution Profiling

For each prognostic predictors, compute its origin moments (mean $\mu_X = \frac{1}{n}\sum_{i=1}^{n} X_i$, variance $\sigma_X^2 = \frac{1}{n}\sum_{i=1}^{n}(X_i - \mu_X)^2$) and central moments (skewness, kurtosis) to fully capture those distributional characteristics across the source domain S and target domain T.These moments reveal both location and dispersion, providing the statistical foundation for cross-domain feature alignment to reduce distributional shift.

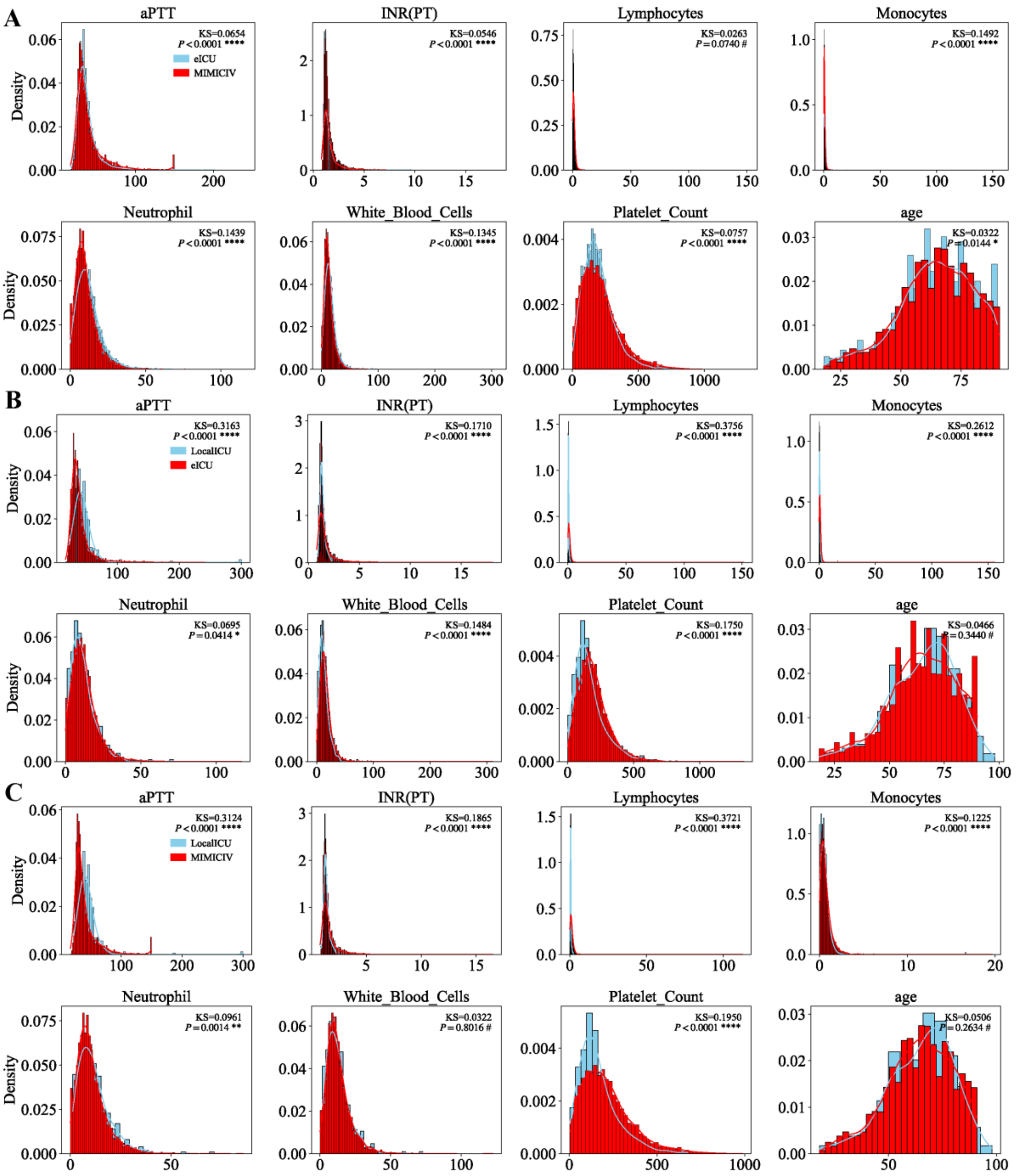
A
B
C
aPTT
INR(PT)
Lymphocytes
Monocytes
Neutrophil
White_Blood_Cells
Platelet_Count
age
Density
eICU
MIMICIV
LocalICU
KS=0.0654
P < 0.0001 ****
KS=0.0546
KS=0.0263
P = 0.0740 #
KS=0.1492
KS=0.1439
KS=0.1345
KS=0.0757
KS=0.0322
P = 0.0144 *
KS=0.3163
KS=0.1710
KS=0.3756
KS=0.2612
KS=0.0695
P = 0.0414 *
KS=0.1484
KS=0.1750
KS=0.0466
P = 0.3440 #
KS=0.3124
KS=0.1865
KS=0.3721
KS=0.1225
KS=0.0961
P = 0.0014 **
KS=0.0322
P = 0.8016 #
KS=0.1950
KS=0.0506
P = 0.2634 #

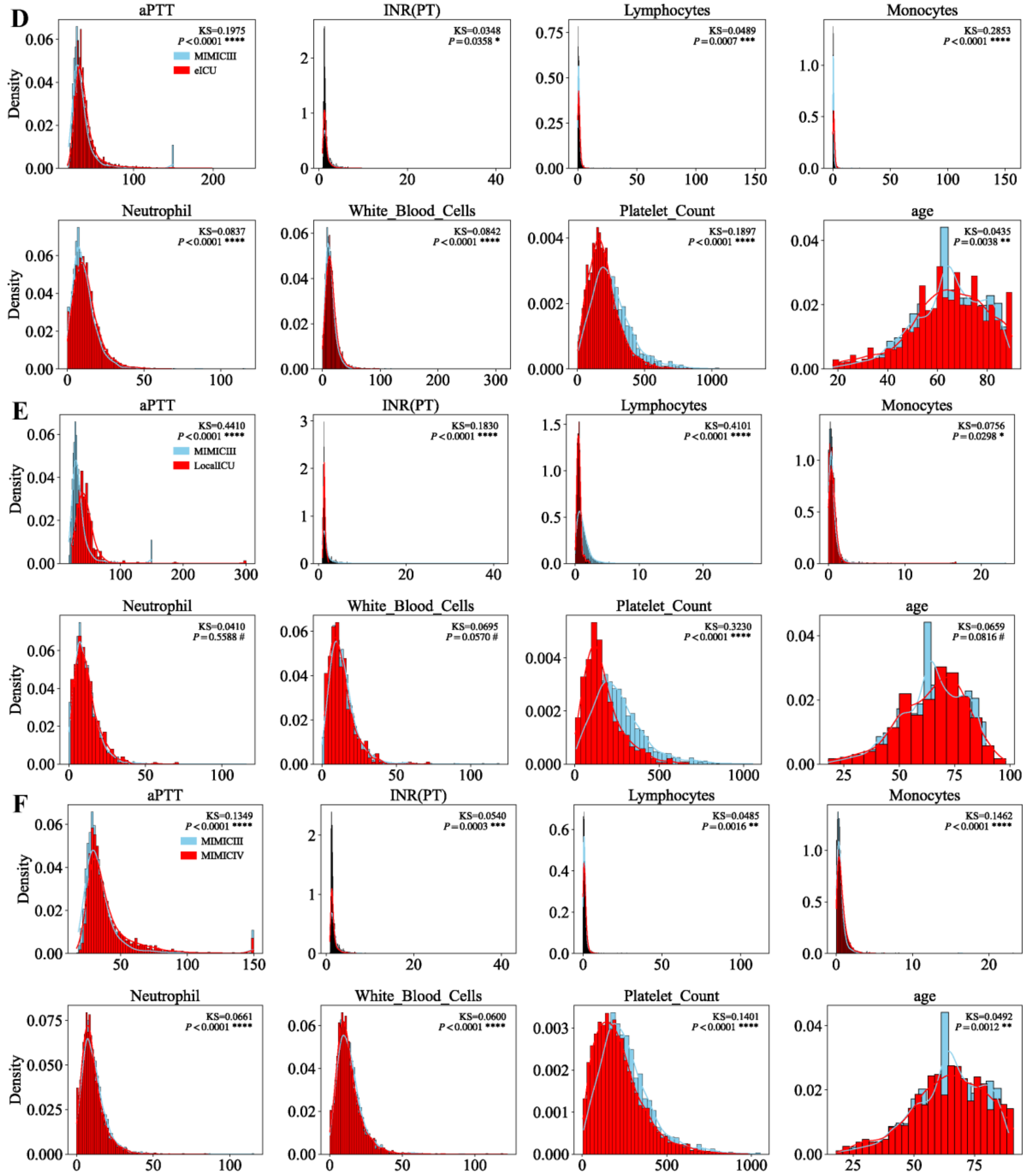

Supplementary Figure for Method. Multi-center covariate distribution shift analyses. Data pairs used for these analyses are A, eICU and MIMICIV. B, Local ICU and eICU. C, Local ICU and MIMICIV. D, MIMICIII and eICU. E, MIMICIII and eICU. F, MIMIC III and MIMIC IV.

Step 2: Clinical Regularization to Address Heteroscedasticity

Considering the biological variability in medical data (e.g., laboratory test results), features exhibiting excessive variance (e.g., $\sigma^2 > 100$) are subject to a logarithmic

transformation to stabilize distribution and mitigate heteroscedasticity without compromising the internal correlation structure:

$$X' = \log_{10}(X+1) \quad (2)$$

In this study, this adjustment was necessary only for the White Blood Cell (WBC) count, which showed extreme dispersion across centers—a known clinical variability marker in sepsis patients.

Step 3: Moment-Based Cross-Domain Projection and Alignment

To reduce distributional divergence between domains, MMID-SMOTE employs moment normalization and projection, aligning the target domain T data into the statistical space of the source domain S:

$$X_T^{\text{aligned}} = \frac{X_T - \mu_T}{\sigma_T} \times \sigma_S + \mu_S \quad (3)$$

This operation ensures domain-invariant feature learning, prevents the generation of outlier samples, and aligns the data distributions—a fundamental requirement in domain adaptation theory for machine learning models applied to heterogeneous clinical data.

Step 4: Min-Max Interval Constraint Definition for SMOTE

Given that the KS test relies on the maximum vertical distance of the empirical cumulative distribution function (ECDF) $D = \max\|\mathrm{F}_1(\mathrm{x}) - \mathrm{F}_2(\mathrm{x})\|$, and as evidenced by Supplementary Tables 3 and 4, the data exhibit substantial range discrepancies (e.g., maximum and minimum values). We define a strict min-max interval constraint for each predictor to maintain clinical plausibility.

$$X_{\min} = \min\left(X_S, X_T\right), \quad X_{\max} = \max\left(X_S, X_T\right) \quad (4)$$

Synthetic instances must satisfy:

$$X_{\text{new}} \in \left[X_{\min}, X_{\max}\right] \quad (5)$$

This constraint respects both statistical boundaries and medical interpretability, ensuring that no biologically implausible values are introduced into the dataset—a key consideration in real-world clinical AI applications.

Step 5: Customized SMOTE Sample Generation

Finally, synthetic samples are generated via SMOTE under these constraints, with the following parameters optimized for medical data context.

Number of nearest neighbors : 4. Minority class oversampling ratio: 1:1 (achieving balanced class distribution).Random seed (random_state): Fixed for reproducibility. Parallel jobs: 3 (accelerating computation).Each new sample is computed as:

$$X_{\text{new}} = X_i + \delta \times (X_j - X_i) \quad (6)$$

where X_i and X_j are minority class instances within the $k = 4$ nearest neighbors and

$\delta \sim U(0,1)$ .The synthetic point is only accepted if $X_{\text{new}} \in [X_{\min}, X_{\max}]$.This ensures the synthetic data remain both statistically valid and clinically feasible.

B. Encoder for feature extraction.

The feature extraction layer of SepsisFormer consists of an integrated network of *K* (*K*=8) Transformer encoders. The feature extraction process is divided into five sections, as follows:

Section 1: Embedding. The normalized indicators $Z^{Xj} = \{\overline{x_j^i}, d^i\} \in \mathbb{R}^{j \times 36}$ are used as input to the feature extraction layer. Let the column vector $\{i \mid 1 \le i \le 35\}$, the row vector $\{j | 1 \le j \le 3811\}$. Let (Z^{Xj}, Y_j) represent indicator-target pair, and each indicator is encoded $E_\varphi(Z^{Xj})$ using Linear Layer, as explained in detail below:

$$E_\varphi(Z^{Xj}) = L(Z^{Xj}) = W_L^T Z^{Xj} + b_L \tag{7}$$

Where $W_L^T \in \mathbb{R}^{36 \times 36}, b_L \in \mathbb{R}^{36}$ is the learnable parameter matrix in the neural network.

Section 2: Indicator dependence. The encode indicators $E_\varphi(Z^{Xj})$ are then inputted to the first Transformer Encoder layer. The output of the first Transformer Encoder layer is then passed onto the second layer, and so on. Each Transformer Encoder layer consists of two sublayers. In the first sublayer, the self-attention mechanism extracts the long-term dependencies between indicators in the sepsis predictors through linear transformation and inner product calculation between $Q^{Xj} \in \mathbb{R}^{m \times k}$, $K^{Xj} \in \mathbb{R}^{m \times k}$, $V^{Xj} \in \mathbb{R}^{m \times v}$. Specifically, the q_t^{Xj} of indicator $E_\varphi(Z^{Xj})$ interacts with the k_l^{Xj} of other indicators in the patient's sepsis predictors, and the attention weight score $\alpha_{t,l}^{Xj} \in \mathbb{R}^{m \times k}$ is used to quantify the long-term dependency relationship:

$$\begin{cases} \alpha_{t,l}^{Xj} = sorce(q_t^{Xj}, k_l^{Xj}) = \{\dfrac{q_t^{Xj} \times k_l^{Xj}}{\sqrt{d_k}}\} \\ \alpha^{Xj} = softmax(\alpha_{t,1}^{Xj}, \alpha_{t,2}^{Xj}, \cdots, \alpha_{t,d_k}^{Xj}) \end{cases} \tag{8}$$

Among $Q^{Xj} = (q_1^{Xj}, q_2^{Xj}, \cdots q_{d_k}^{Xj}) = W_q^{Xj} E_\varphi(Z^{Xj})$, $K^{Xj} = (k_1^{Xj}, k_2^{Xj}, \cdots k_{d_k}^{Xj}) = W_k^{Xj} E_\varphi(Z^{Xj})$; Where $W_q^{Xj} \in \mathbb{R}^{m \times m}$ and $W_k^{Xj} \in \mathbb{R}^{m \times m}$ respectively represent the Q^{Xj}, K^{Xj} projection weight matrix corresponding to the input sequence $E_\varphi(Z^{Xj})$.

The self-attention output $\partial_t^{Xj} \in \mathbb{R}^m$ corresponding to the indicator $E_\varphi(Z^{Xj})_t$ is obtained by weighted summation of $\alpha_{t,l}^{Xj} \in \mathbb{R}^{m \times k}$ and $v_l^{Xj} \in \mathbb{R}^m$:

$$\partial_t^{Xj} = \sum_{l=1}^{c} \alpha_{t,l}^{Xj} v_l^{Xj} \tag{9}$$

Where, $V^{Xj} = (v_1^{Xj}, v_2^{Xj}, \cdots, v_m^{Xj}) = W_v^{Xj} I^{Xj}$ and $W_v^{Xj} \in \mathbb{R}^{m \times m}$ represents the V^{Xj} projected weight matrix corresponding to the input sequence $E_\varphi(Z^{Xj})$.

Section 3: Enhanced sepsis predictors. This paper utilizes multi-head attention to simultaneously integrate the long-term dependencies of sepsis predictors from different levels and perspectives, thereby enhancing the feature extraction capability of the model. All single-headed outputs are aggregated, and a local dependent feature $\partial^{Xj} = \{\partial_1^{Xj}, \partial_2^{Xj}, \cdots, \partial_m^{Xj}\}$ is extracted. A linear layer is then used to project the dimension back to its original size. The global long-range dependent eigenvector $\delta^{x^i} \in \mathbb{R}^m$ is obtained,

as follows:

$$\delta^{X^j} = MultiHead(Q^{X^j}, K^{X^j}, V^{X^j}) = Concat[\partial_{h,1}^{X^j}, \partial_{h,2}^{X^j}, \cdots, \partial_{h,d_{\text{head}}}^{X^j}] \times W_{\text{head}} \quad (10)$$

Where $\partial_{h,j}^{X^j}$ represents the single-headed attention output, d_{head} denotes the number of multiple heads, and $W_{\text{head}} \in \mathbb{R}^{d_{\text{head}} m \times m}$ is the learnable projective parameter matrix.

Section 4: Information fusion. These long-range dependent features δ^{x^i} are directly added across levels to the current input $E_{\varphi}(Z^{X^j})$, and then passed through the Layer Normalization. This process is followed by the final output $\alpha^{X^j} \in \mathbb{R}^{1\times m}$ of the first sublayer of Transformer Encoder layer.

$$\alpha^{X^j} = LN(E_{\varphi}(Z^{X^j}) + \delta^{X^j}) = LN(E_{\varphi}(Z^{X^j}) + MHA(LN(E_{\varphi}(Z^{X^j})))) \quad (11)$$

Section 5: Feed-Forward Network. In the Feed-Forward Network sublayer, the output passes through two dense layers. The first dense layer maps the global long-range dependent feature $\alpha_{first}^{X^j}$ to a size five times larger, thereby increasing the feature space dimension and improving the model's ability to fuse sepsis predictor information. The second dense layer projects it back to its original size. The final output of the Transformer Encoder layer obtained in this paper is given by:

$$I^{X^j} = \alpha^{X^j} + \left[W_{\text{head1}}^T (LN(\alpha^{X^j}) + b_{\text{head1}}) \times \Phi(W_{\text{head1}}^T (LN(\alpha^{X^j}) + b_{\text{head1}}))\right] W_{\text{head2}} + b_{\text{head2}} \quad (12)$$

Where $W_{\text{head1}}^T \in \mathbb{R}^{m\times m}, W_{\text{head2}}^T \in \mathbb{R}^{m\times m}$, $b_{\text{head1}} \in \mathbb{R}^{m\times m}, b_{\text{head2}} \in \mathbb{R}^{m\times m}$ is the learnable parameter matrix in the neural network. $\Phi(x)$ stands for GELU activation function.

Prognostic prediction. In this paper, a Multi-Layer Perceptron (MLP) is used as a prognostic predictor in SepsisFormer. The indicator information I^{X^j} in the sepsis predictors is fused and flattened, resulting in a two-channel feature vector. This future vector, $I_{\Phi}^{X^j}$, is then mapped to a higher dimensional hidden state $H_{\Phi}^{x^i} \in \mathbb{R}^m$, using a Linear-layer of 64 neurons. To introduce nonlinearity and prevent overfitting, the GELU activation function is applied:

$$H_{\Phi}^{X^j} = \Phi(W_{\text{mlp1}}^T (I_1^{X^j}, I_2^{X^j}, \cdots, I_8^{X^j}) + b_{\text{mlp1}}) \quad (13)$$

Where $W_{\text{mlp1}}^T \in \mathbb{R}^{m\times 64}$, $b_{\text{mlp1}} \in \mathbb{R}^{m\times 64}$ is the learnable parameter matrix, $\Phi(x)$ stands for the GELU activation function.

Subsequently, the context vector is predicted through a fully connected layer for binary classification. Finally, the mortality rate of patients was obtained through Softmax function:

$$\begin{cases} \beta_{\Phi}^{X^j} = W_{\text{mlp2}}^T H_{\Phi}^{X^j} + b_{\text{mlp2}} \\ Y^{X^j} = softmax(\beta_1^{X^j}, \beta_2^{X^j}) = e^{\beta_1^{X^j}} / (e^{\beta_1^{X^j}} + e^{\beta_2^{X^j}}) \end{cases} \quad (14)$$

Where $W_{\text{mlp2}}^T \in \mathbb{R}^{64\times 2}$, $b_{\text{mlp2}} \in \mathbb{R}^{64\times 2}$ denotes the learnable parameter matrix in the neural network.

C. Objective function.

To optimize model training, we utilize a label-smoothed cross-entropy loss function. This approach addresses the variations in sepsis predictors in the MIMIC dataset. The cross-entropy loss function measures the discrepancy between prognostic predictions and true labels. Additionally, label smoothing penalizes overly confident prognostic predictions,

which helps mitigate overfitting. The formula for the label-smoothed cross-entropy loss is as follows:

$$\mathcal{L} = -\frac{1}{\mathrm{N}}\sum_{i=1}^{\mathrm{N}}\sum_{k=1}^{2}\log\left(p_k\right)q'_k = \left(1-\varepsilon\right)\mathcal{L}\left(q,p\right)+\varepsilon\mathcal{L}\left(u,p\right)$$
$$= (1-\varepsilon)\times[-\frac{1}{\mathrm{N}}\sum_{i=1}^{\mathrm{N}}\sum_{k=1}^{2}\log\left(p_k\right)\delta_k]+\varepsilon\times[\frac{1}{\mathrm{N}}\sum_{i=1}^{\mathrm{N}}\sum_{k=1}^{2}\log\left(p_k\right)\frac{1}{k}] \quad (15)$$

Where q represents the true label, p denotes the prognosis prediction obtained from SepsisFormer, and N represents the number of patients.

D. Model evaluation metrics.

In this study, the SepsisFormer model developed in this paper is compared with traditional machine learning models including Random Forest (RF), Decision Tree (DT), Support Vector Machine (SVM), Logistic Regression (LR), Stochastic Gradient Descent (SGD), as well as deep learning models such as Long Short-Term Memory (LSTM), Gated Recurrent Unit (GRU), and GPT_1. Prognostic prediction performance is evaluated using metrics such as area under the curve (AUC), accuracy, specificity, sensitivity, and F1-score.

Supplementary Method 2. DEGs screening steps and RT-qPCR experimental steps.

The sepsis expression profile was downloaded from the Gene Expression Omnibus (GEO) (https://www.ncbi.nlm.nih.gov/geo/) database, and GSE65682 was selected. DEGs between sepsis (n = 760) and healthy control (n = 42) blood samples were identified using "limma" R package. Subsequently, the candidate biomarkers were identified through the intersection of CRGs, IRGs, and DEGs. We analyzed the obtained intersection genes based on KEGG, GO, and protein-protein interactions (PPI) to understand the underlying biological mechanisms and establish a significant association.

Four hundred seventy-nine sepsis patients with complete survival data were enrolled and identified DEGs between septic survivors (n = 365) and non-survivors (n = 114) transcriptomes, which were genetically analyzed using gene set enrichment analysis (GSEA). Subsequently, the candidate biomarkers were identified through the intersection of DIC-related genes and DEGs. Furthermore, an analysis of the PPI of these intersecting genes was conducted.

mRNA was isolated from PBMCs using TrasZol Up (TransGen, ET111-01-V2, China) following the manufacturer's instructions. The RNA concentration was determined using a Nano-400a spectrophotometer (ALLSHENG, Hangzhou Allsheng Instruments Co., Ltd., China). For reverse transcription and cDNA synthesis, 0.5ug RNA was used with the one-step cDNA reverse transcription kit (TransGen, AE311-03, China) following the manufacturer's instructions. The qPCR experiment was conducted on the 7500 Real-Time PCR System (ABI, Thermo Fisher Scientific) using the SYBR-Green mixture (TransGen, AQ601-02-V2, China) with the following thermocycling conditions: 40 cycles at 94°C for 5 seconds and 60°C for 30 seconds. Relative mRNA quantities were normalized by GAPDH expression. The primer sequences (synthesized by Shanghai Jierui Bioengineering Co., Ltd., catalog number kr001) used in the RT-qPCR are listed in Supplementary Table 15.

Supplementary Method 3. External validation of diagnostic performance of the DGEs.

To independently validate the diagnostic efficacy of the identified four-gene signature, we utilized two publicly available gene expression datasets: GSE26440 and GSE95233, serving as independent validation cohorts. First, differential expression analysis of the four candidate genes between sepsis patients and control subjects within each dataset was performed the limma R package. Subsequently, the diagnostic performance of this four-gene signature for discriminating sepsis patients from controls was rigorously evaluated. ROC curves were generated and the AUC was calculated using the pROC package in R.

Supplementary Method 4. External validation of prognostic performance of the DGEs.

To independently validate the prognostic efficacy of the identified five-gene signature, we utilized the publicly available gene expression dataset GSE54514 as an independent validation cohort. First, differential expression analysis of the five candidate genes between sepsis patients and control subjects was performed using the limma R package. Subsequently, the prognostic performance of this signature in stratifying sepsis patients by survival outcome was rigorously evaluated. ROC curves were generated and the AUC was calculated using the pROC package in R.

Supplementary Method 5. Detailed algorithm derivation of SMART.

In this retrospective study, we advanced risk stratification by refining it through high-risk and low-risk sepsis patients, creating the Sepsis Mortality And Risk Tool (SMART) model and developing a corresponding web-based risk scoring system. The scoring system primarily employs a lasso-based feature selection method driven by medical knowledge. The core code is implemented using scorecardpy Python package (https://github.com/ShichenXie/scorecardpy version 0.1.9.6). The simplified procedure of medical knowledge-driven supervised SMLR can be outlined as follows.

Data preprocessing and splitting. Seven coagulation-inflammatory markers and age are considered risk variables, and survivor/non-survivor is the target variable. Data augmentation was employed on MIMIC-III and IV to address the issue of imbalanced mortality rates, resulting in a total of 9,799 sepsis patients used for supervised modeling. Data are encoded into numerical format and then split into training and testing sets at 7:3.

Medical knowledge-driven variable binning. Ordered continuous variables are discretized using the supervised bottom-up method ChiMerge. However, ChiMerge tends to generate a larger number of bins. In this study, a medical knowledge-driven criterion is proposed for optimizing bins. Firstly, variable binning is performed, and χ^2 tests are then conducted on all pairs of initial intervals. Those adjacent bins with $p < 0.05$ are merged. Secondly, the information value for each binned variable is calculated to predict the power of each variable about the target variable--survivor or non-survivor. When the number of bins ranges from 2 to 8, the inflection point on the curve formed by the corresponding information value is identified as the optimal number of bins. Thirdly, the optimal binning points are identified based on the lower and upper limits of the normal range (as specified in Table 2) for each risk factor. Pseudo codes are provided.

Weight of evidence (WOE) transformation, information value (IV), and lasso-based logistic regression. The WOE transformation and IV are employed to assess and explain the association between risk factors and the predictive event. WOE converts the original markers into a more informative and predictive format, indicating the difference between the "proportion of survivors in the current bin among all survivors" and the "proportion of non-survivors in the current bin among all survivors." IV reflects the ability of the risk factor to distinguish between survival and non-survival under the current binning method, with higher IV values indicating more substantial prognostic capabilities for that factor. The formulas are as follows：

$$\mathrm{woe}_{ij} = \ln\left(\frac{s_{ij}/s_{iT}}{d_{ij}/d_{iT}}\right) \tag{16}$$

$$iv_j = \sum_{i=1}^{N}\left(\frac{s_{ij}}{s_{iT}} - \frac{d_{ij}}{d_{iT}}\right) \times woe_{ij} \tag{17}$$

Wherein, s_{ij} and d_{ij} denote the number of survivors and non-survivors in the ith bin for

the ith coagulation-inflammatory marker, while, s_{iT} and d_{iT} represent the total number of survivors and non-survivors in the ith bin for the entire sample.

Considering the heterogeneity of the data, logistic regression with LASSO penalty is developed to reduce the impact of abnormal data on the model. Lasso regularization mitigates high variance in logistic regression coefficients with numerous correlated parameters. The parameter λ of L1 regularization can be adjusted to control the sparsity of coefficients. A larger λ leads to more feature coefficients becoming zero, reducing the variance of coefficients and facilitating feature selection. The lasso-based logistic regression models are expressed as follows:

$$\min_{\beta} \left\{ \sum_{i=1}^{N} \left[y_i \log(p_i) - (1-y_i) \log(1-p_i) \right] - \lambda \sum_{j=1}^{m} \left| \beta_j \right| \right\} \quad (18)$$

Where y_i are the responses, p_i is the probability, λ are the coefficients, and λ is the complexity parameter that controls the strength of regularization. Cross-validation is employed to dynamically choose an optimal λ that minimizes the deviance.

Scorecard development and model evaluation

To visualize and interpret clinical application, we mapped its results to a scorecard.

$$score = A - B \times \ln(odds) \quad (19)$$

Where A is the score when probabilities of non-survival and survival are equal, B is a margin while $score$ against $\ln(odds)$, $odds$ is a probability of non-survival/survival probability.

We set a target score po, when $odds = odds_0$, the point-to-double odds (pdo) refer to the difference when the $odds$ is halved. Here, $po = 10$, $odds_0 = 2$ and $pdo = 2$. These initialization factors were brought back into the following formula.

$$po = A - B \times \ln(odds_0) \quad (20)$$

$$po + pdo = A - B \times \ln(\mathrm{odds}_0/2) \quad (21)$$

Then,

$$B = \frac{pdo}{\ln 2} \quad (22)$$

$$A = po + B \times \ln(odds_0)$$

Based on the setting above, the score function can be deduced as follows.

$$score = \left[\left(po + \frac{pdo}{\ln 2} \times \ln(odds_0) \right) - \frac{pdo}{\ln 2} \times \ln(odds) \right] \quad (23)$$

Web-based risk stratification system

A visual web interface was developed using HTML5 and jQuery 3.2.1. An interactive method was employed to compute the risk score and define the risk level of patients.

Supplementary Method 6. Pseudocode of discretization based on ChiMerge.

Algorithm 1: Pseudocode of our proposed discretization algorithm based on ChiMerge.

Input: A is MIMIC data set. The max num of bin. Merge the critical p-Value of adjacent bins. The minimum percentage of final binning class number over total.

Output: Optimal binning dataframe.

```
procedure Discretization(A, minN)
    for A[i] ∈ [A[aPTT],A[INR],…,A[age]] do
        sorted(A[i])
        A[i] = set(A[i]) // Duplicate removal
        while p < 0.05 and numbin>minN and minimum percentage < 0.05 do
            numbin = len(A[i])
            for j ∈ A[i] do
                chivalue[j] = chi square test(merge(A[i][j], A[i][j+1]))
            end for
            min(chivalue[j])
            merge(A[i][j], A[i][j+1]
            numbin = len(A[i])
            the number percentage of people in each bin
        end while
        calculation iv and the optimal number of bins
    end for
    return Optimal bin
end procedure
```

Supplementary Method 7. Pseudocode of adjusting bins.

Algorithm 2: Adjust bin.

Input: Bin Splitting Points. The mp is the point of entry based on medical knowledge.

Output: Optimized binning by medical knowledge.

```
procedure Adjustment (A, zp, mp)
    for A[i] ∈ [A[aPTT],A[INR],…,A[age]] do
        sorted(zp,mp)
        delete point of entry within the normal range
        while the survival curve is not monotonic or inverted 'v' shape do
            calculate the survival rate of each bin
            if no difference in survival rate between adjacent bin do
                merge(adjacent bin)
            end if
        end while
    end for
    return Adjustment bin
end procedure
```

Supplementary case material. The external validation cohort of 40 cases of SMART. （**Via SMART risk stratification system:** http://smartsepsis.org.cn)

Case1.

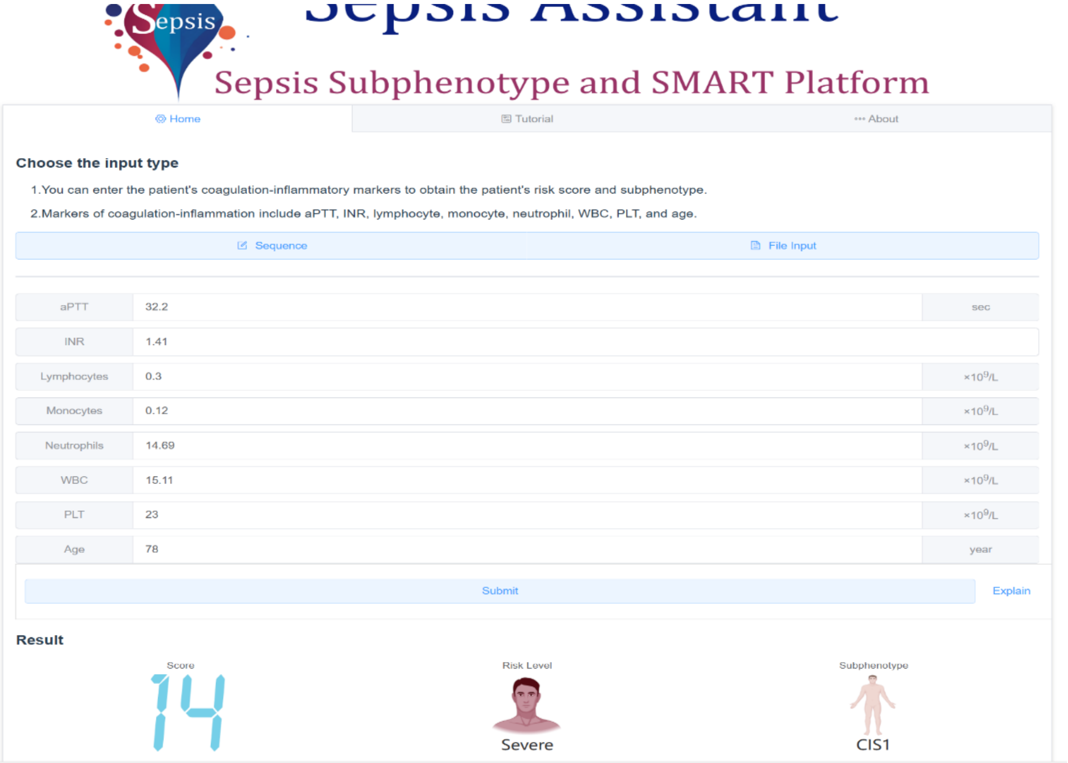

Case2.

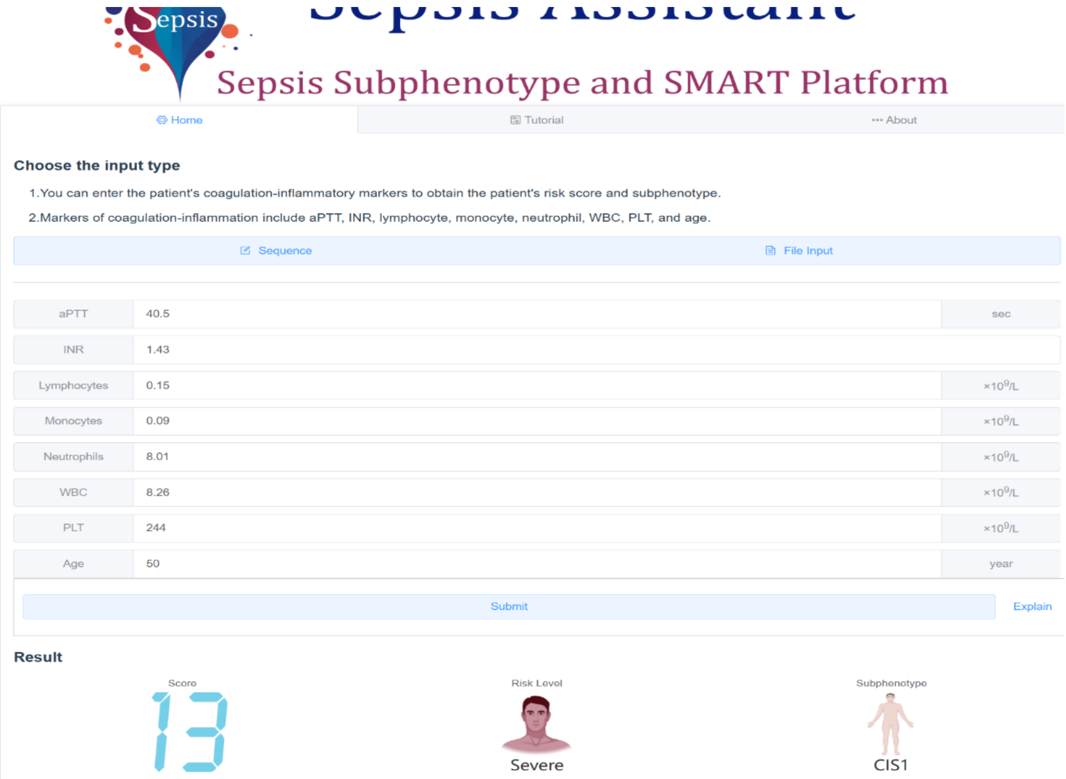
Sepsis
Sepsis Subphenotype and SMART Platform
Home
Tutorial
About
Choose the input type
1.You can enter the patient's coagulation-inflammatory markers to obtain the patient's risk score and subphenotype.
2.Markers of coagulation-inflammation include aPTT, INR, lymphocyte, monocyte, neutrophil, WBC, PLT, and age.
Sequence
File Input
aPTT 40.5 sec
INR 1.43
Lymphocytes 0.15 ×10⁹/L
Monocytes 0.09 ×10⁹/L
Neutrophils 8.01 ×10⁹/L
WBC 8.26 ×10⁹/L
PLT 244 ×10⁹/L
Age 50 year
Submit
Explain
Result
Score
13
Risk Level
Severe
Subphenotype
CIS1

Case3.

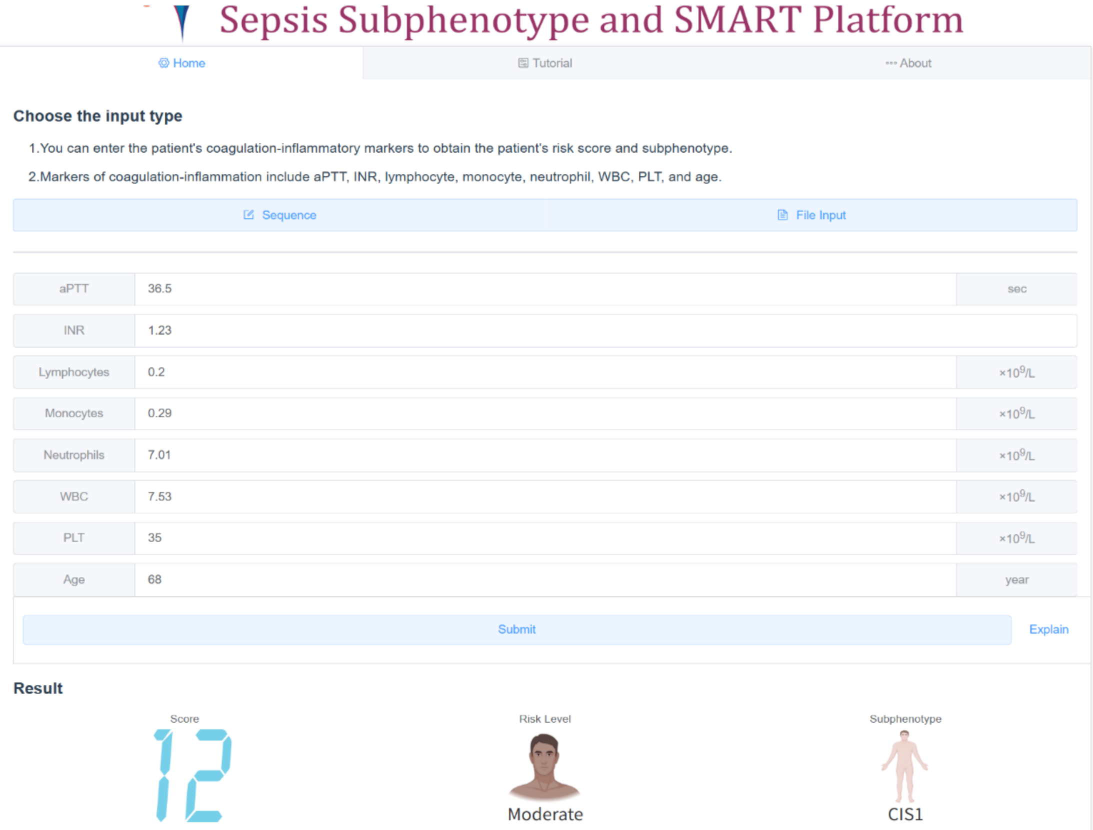
Sepsis Subphenotype and SMART Platform
Home
Tutorial
About
Choose the input type
1.You can enter the patient's coagulation-inflammatory markers to obtain the patient's risk score and subphenotype.
2.Markers of coagulation-inflammation include aPTT, INR, lymphocyte, monocyte, neutrophil, WBC, PLT, and age.
Sequence
File Input
aPTT 36.5 sec
INR 1.23
Lymphocytes 0.2 ×10⁹/L
Monocytes 0.29 ×10⁹/L
Neutrophils 7.01 ×10⁹/L
WBC 7.53 ×10⁹/L
PLT 35 ×10⁹/L
Age 68 year
Submit
Explain
Result
Score
12
Risk Level
Moderate
Subphenotype
CIS1

Case4.

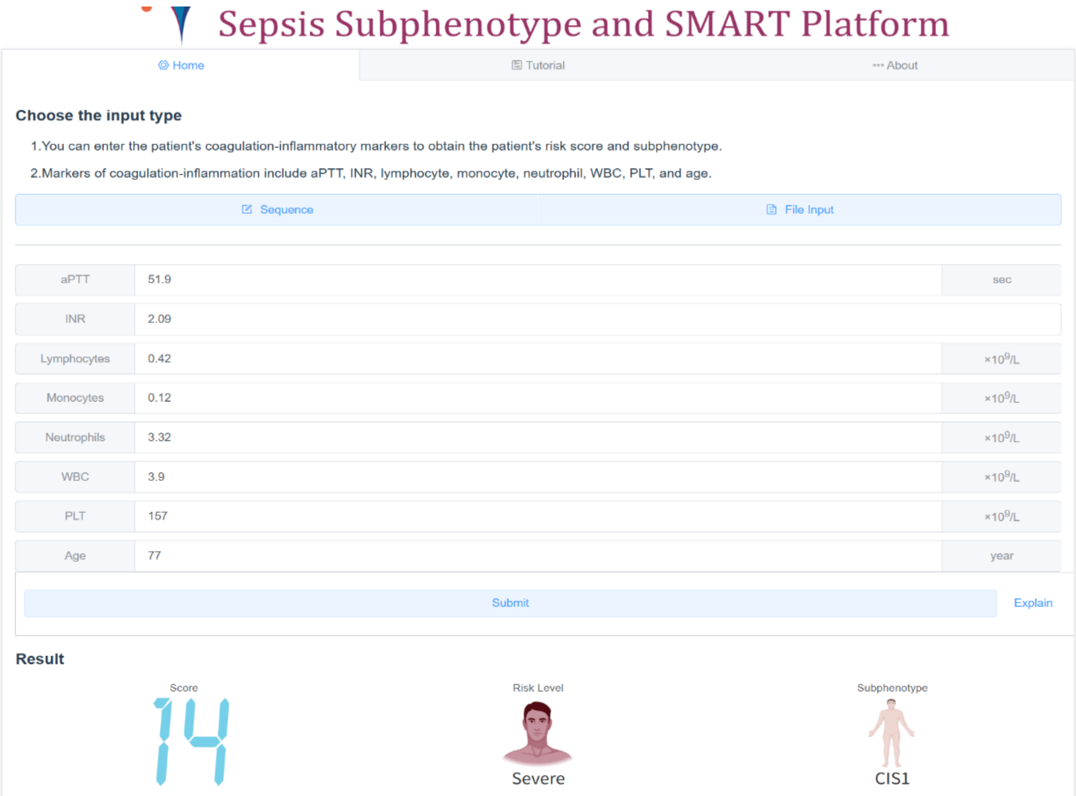
Sepsis Subphenotype and SMART Platform
Home
Tutorial
About
Choose the input type
1.You can enter the patient's coagulation-inflammatory markers to obtain the patient's risk score and subphenotype.
2.Markers of coagulation-inflammation include aPTT, INR, lymphocyte, monocyte, neutrophil, WBC, PLT, and age.
Sequence
File Input
aPTT 51.9 sec
INR 2.09
Lymphocytes 0.42 ×10⁹/L
Monocytes 0.12 ×10⁹/L
Neutrophils 3.32 ×10⁹/L
WBC 3.9 ×10⁹/L
PLT 157 ×10⁹/L
Age 77 year
Submit
Explain
Result
Score
14
Risk Level
Severe
Subphenotype
CIS1

Case5.

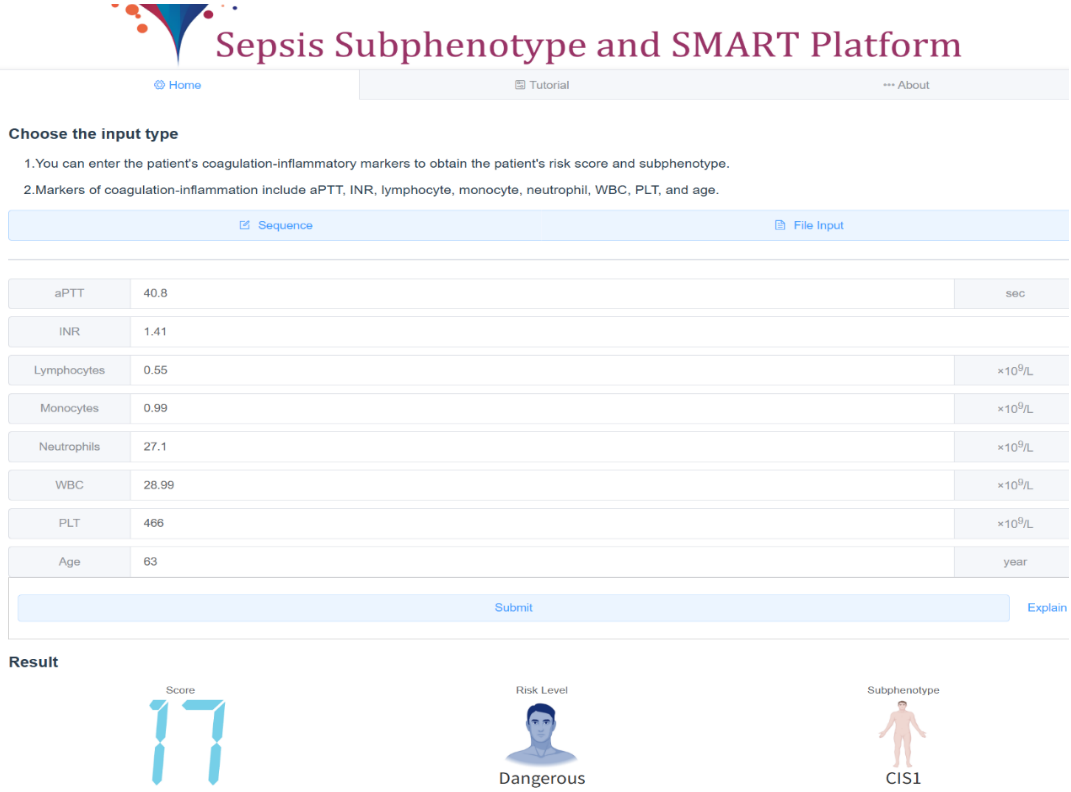
Sepsis Subphenotype and SMART Platform
Home
Tutorial
About
Choose the input type
1.You can enter the patient's coagulation-inflammatory markers to obtain the patient's risk score and subphenotype.
2.Markers of coagulation-inflammation include aPTT, INR, lymphocyte, monocyte, neutrophil, WBC, PLT, and age.
Sequence
File Input
aPTT 40.8 sec
INR 1.41
Lymphocytes 0.55 ×10⁹/L
Monocytes 0.99 ×10⁹/L
Neutrophils 27.1 ×10⁹/L
WBC 28.99 ×10⁹/L
PLT 466 ×10⁹/L
Age 63 year
Submit
Explain
Result
Score
17
Risk Level
Dangerous
Subphenotype
CIS1

Case6.

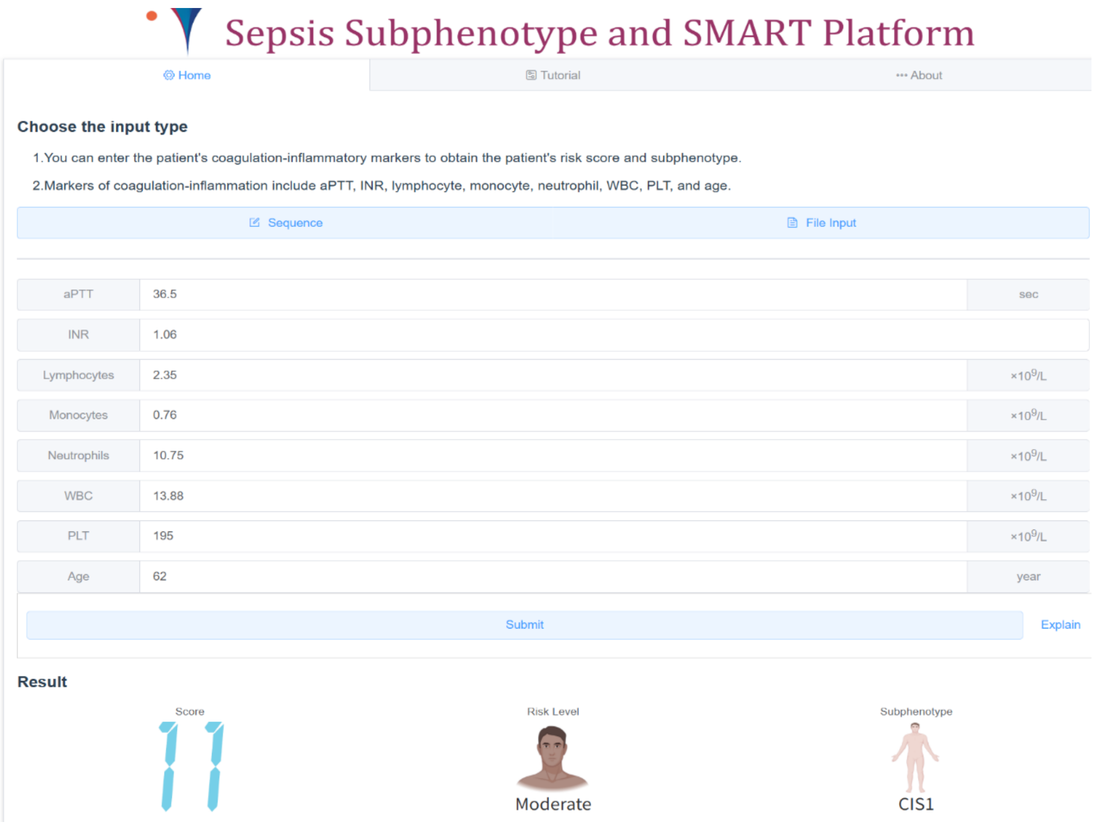
Sepsis Subphenotype and SMART Platform
Home
Tutorial
About
Choose the input type
1.You can enter the patient's coagulation-inflammatory markers to obtain the patient's risk score and subphenotype.
2.Markers of coagulation-inflammation include aPTT, INR, lymphocyte, monocyte, neutrophil, WBC, PLT, and age.
Sequence
File Input
aPTT 36.5 sec
INR 1.06
Lymphocytes 2.35 ×10⁹/L
Monocytes 0.76 ×10⁹/L
Neutrophils 10.75 ×10⁹/L
WBC 13.88 ×10⁹/L
PLT 195 ×10⁹/L
Age 62 year
Submit
Explain
Result
Score
11
Risk Level
Moderate
Subphenotype
CIS1

Case7.

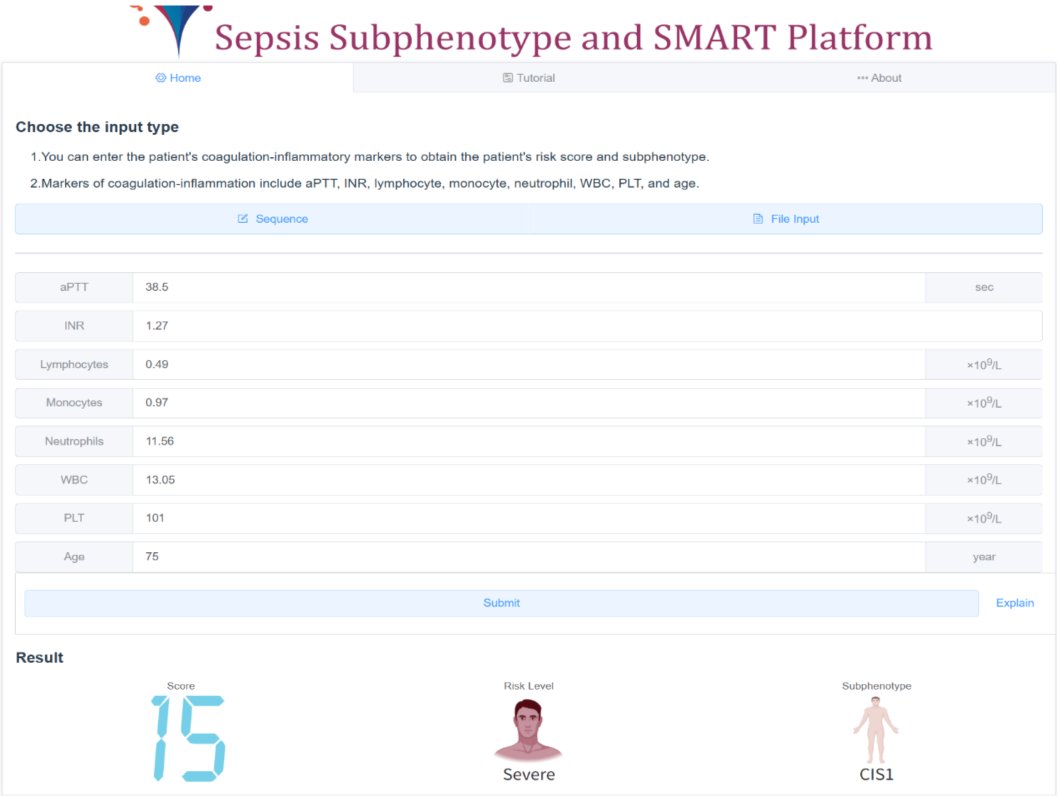
Sepsis Subphenotype and SMART Platform
Home
Tutorial
About
Choose the input type
1.You can enter the patient's coagulation-inflammatory markers to obtain the patient's risk score and subphenotype.
2.Markers of coagulation-inflammation include aPTT, INR, lymphocyte, monocyte, neutrophil, WBC, PLT, and age.
Sequence
File Input
aPTT 38.5 sec
INR 1.27
Lymphocytes 0.49 ×10⁹/L
Monocytes 0.97 ×10⁹/L
Neutrophils 11.56 ×10⁹/L
WBC 13.05 ×10⁹/L
PLT 101 ×10⁹/L
Age 75 year
Submit
Explain
Result
Score
15
Risk Level
Severe
Subphenotype
CIS1

Case8.

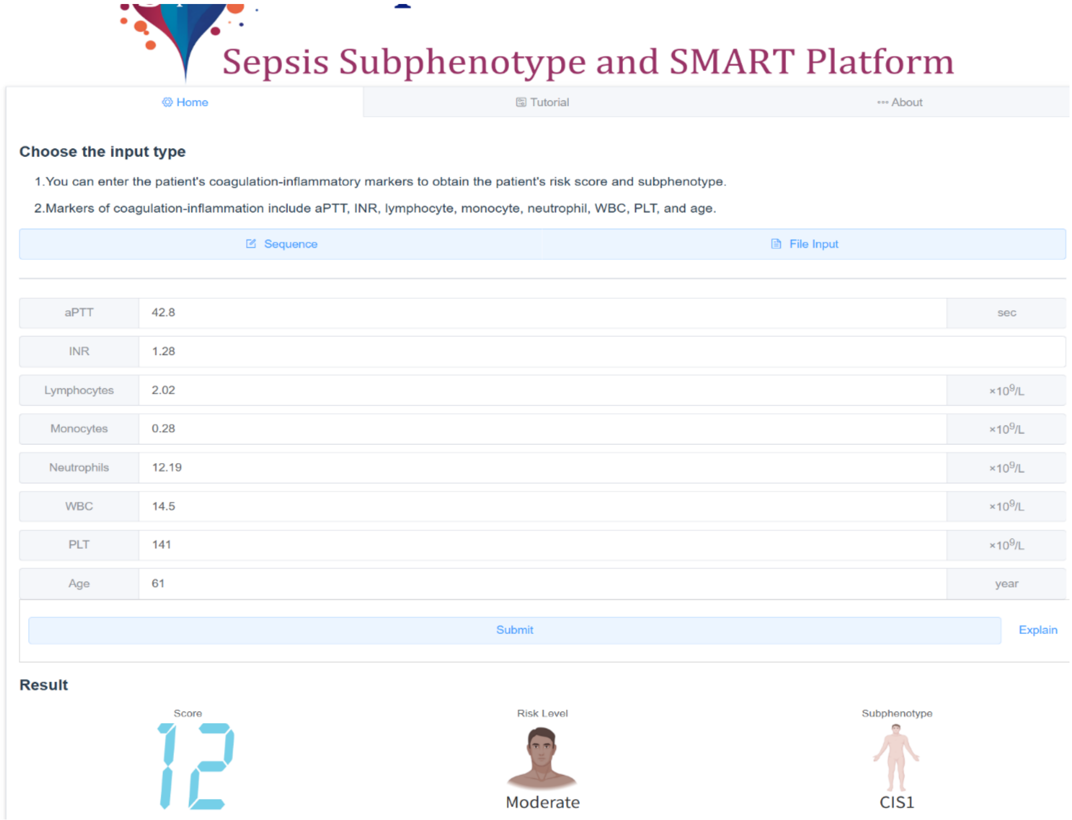
Sepsis Subphenotype and SMART Platform
Home
Tutorial
About
Choose the input type
1.You can enter the patient's coagulation-inflammatory markers to obtain the patient's risk score and subphenotype.
2.Markers of coagulation-inflammation include aPTT, INR, lymphocyte, monocyte, neutrophil, WBC, PLT, and age.
Sequence
File Input
aPTT 42.8 sec
INR 1.28
Lymphocytes 2.02 ×10^9/L
Monocytes 0.28 ×10^9/L
Neutrophils 12.19 ×10^9/L
WBC 14.5 ×10^9/L
PLT 141 ×10^9/L
Age 61 year
Submit
Explain
Result
Score
12
Risk Level
Moderate
Subphenotype
CIS1

Case9.

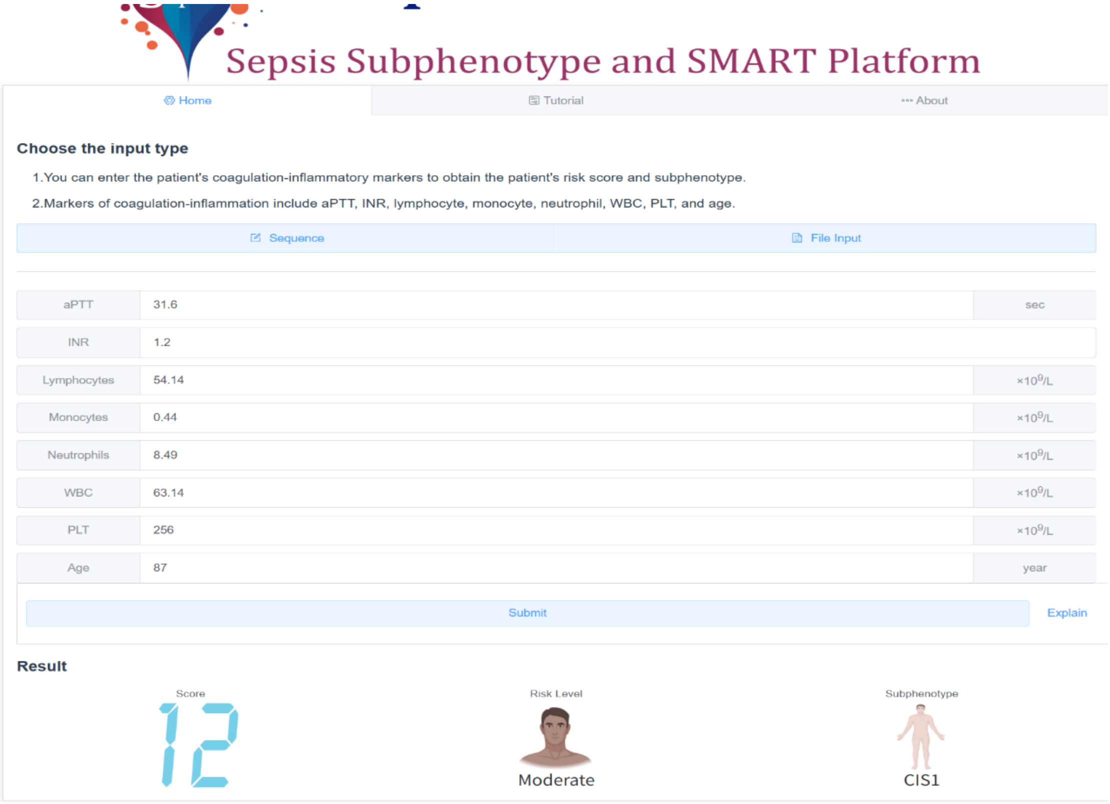
Sepsis Subphenotype and SMART Platform
Home
Tutorial
About
Choose the input type
1.You can enter the patient's coagulation-inflammatory markers to obtain the patient's risk score and subphenotype.
2.Markers of coagulation-inflammation include aPTT, INR, lymphocyte, monocyte, neutrophil, WBC, PLT, and age.
Sequence
File Input
aPTT 31.6 sec
INR 1.2
Lymphocytes 54.14 ×10^9/L
Monocytes 0.44 ×10^9/L
Neutrophils 8.49 ×10^9/L
WBC 63.14 ×10^9/L
PLT 256 ×10^9/L
Age 87 year
Submit
Explain
Result
Score
12
Risk Level
Moderate
Subphenotype
CIS1

Case10.

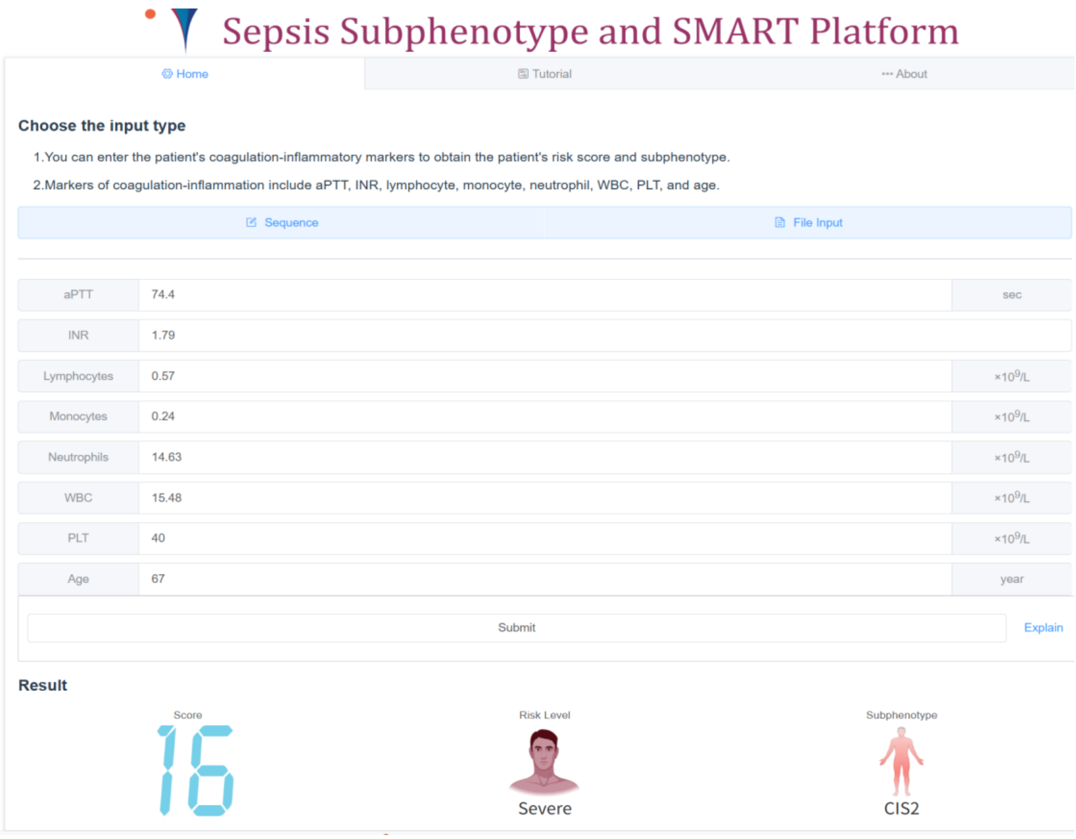

Case11.

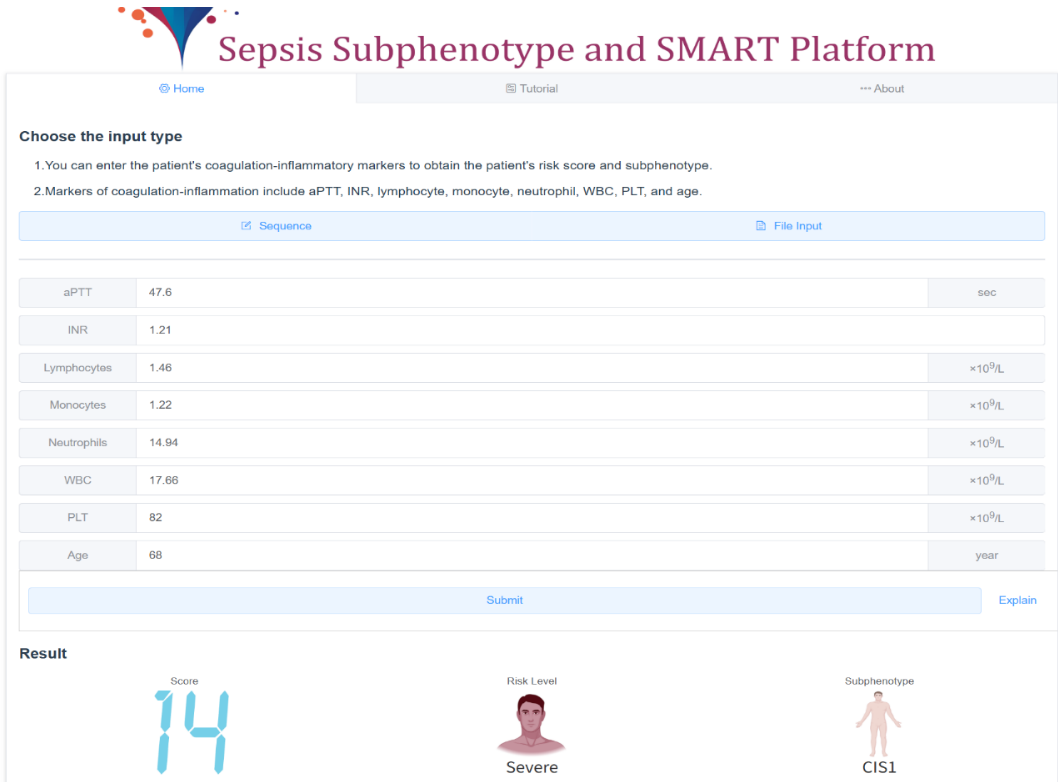

Case12.

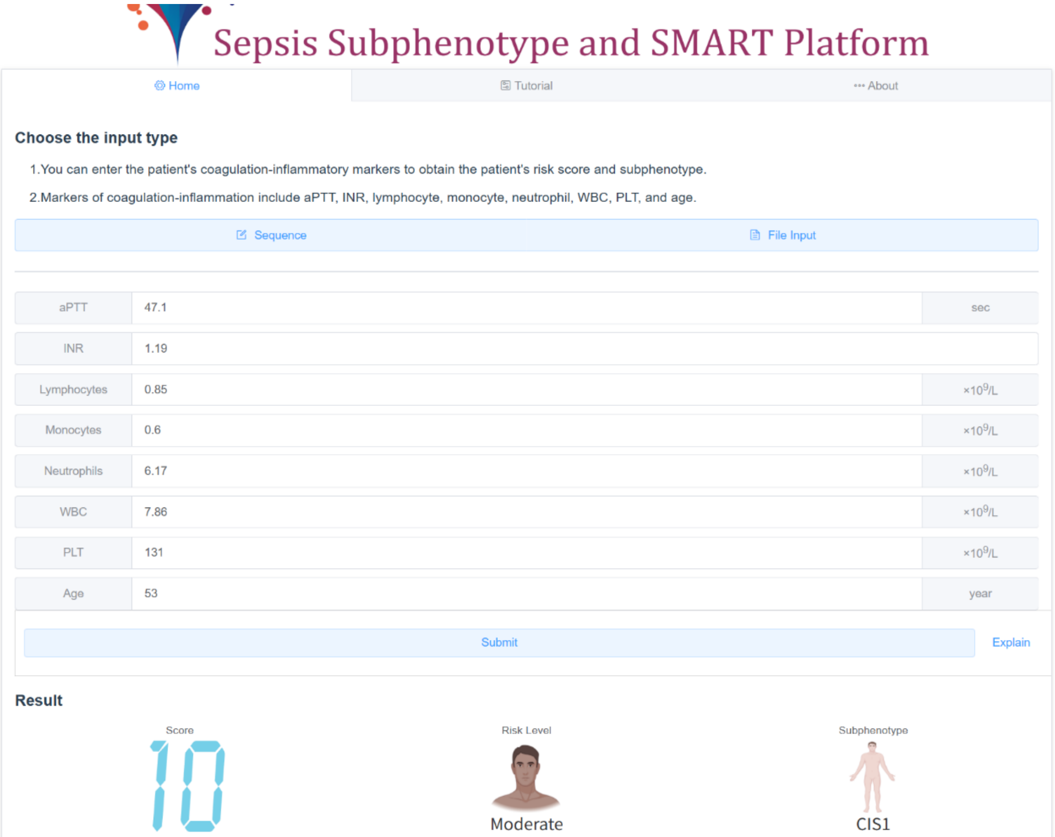

Case13.

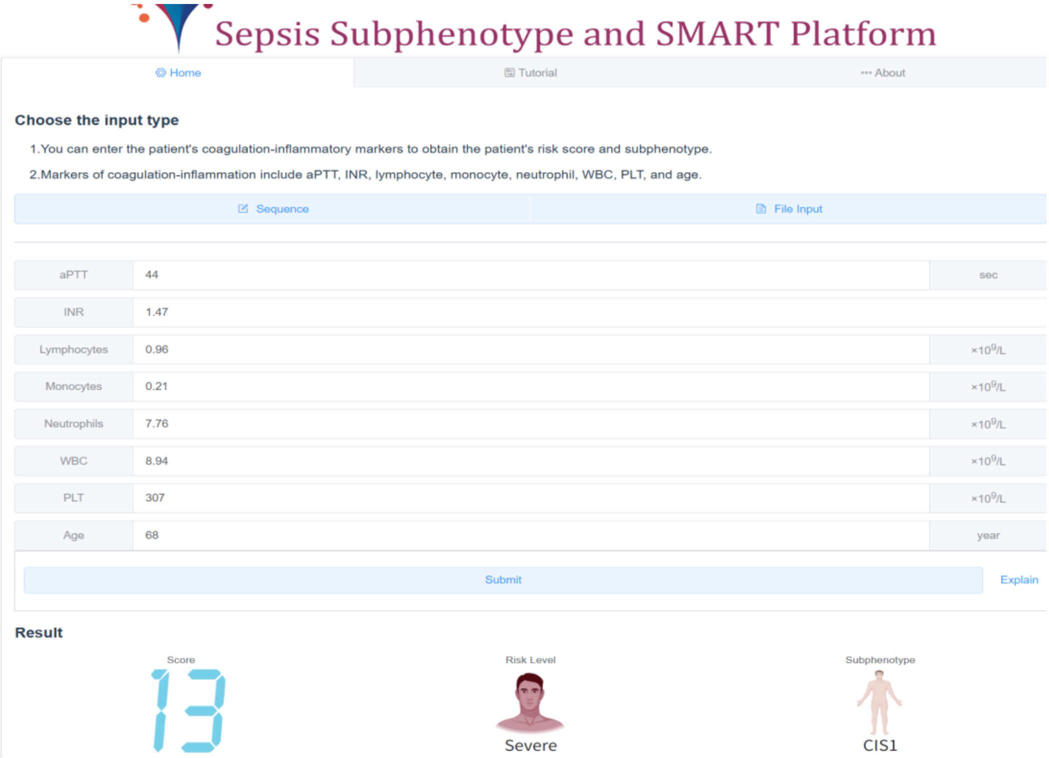

Case14.

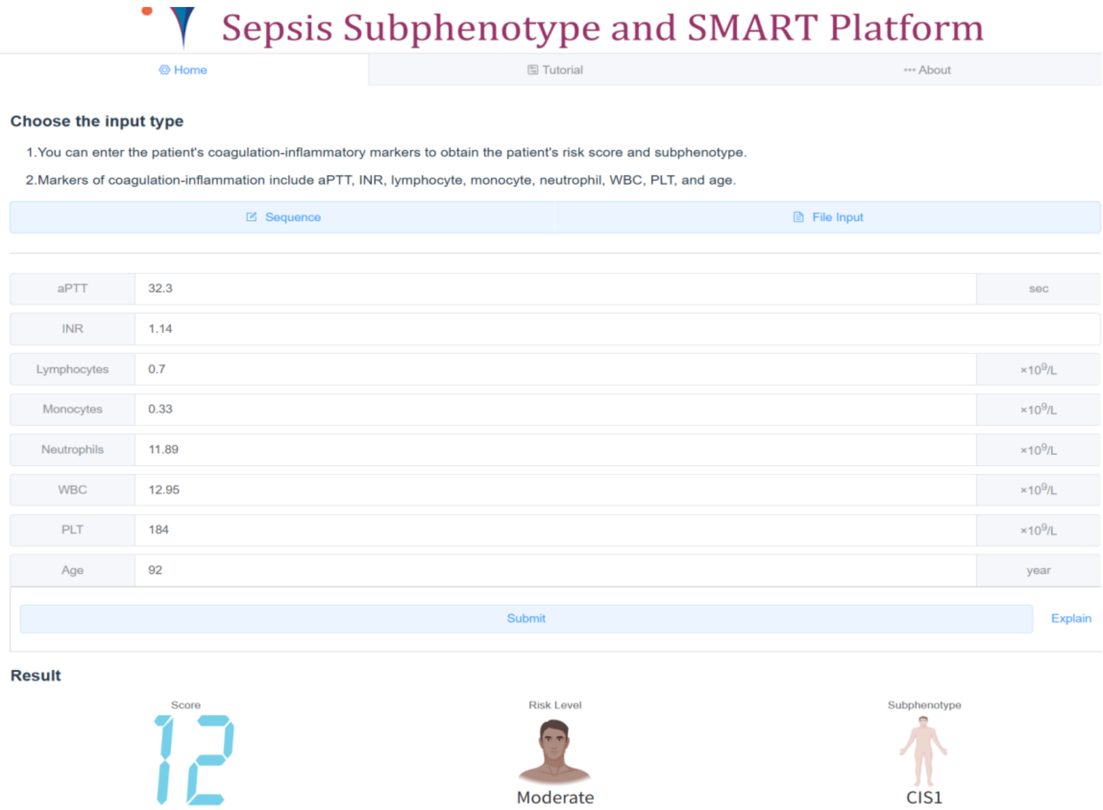

Case15.

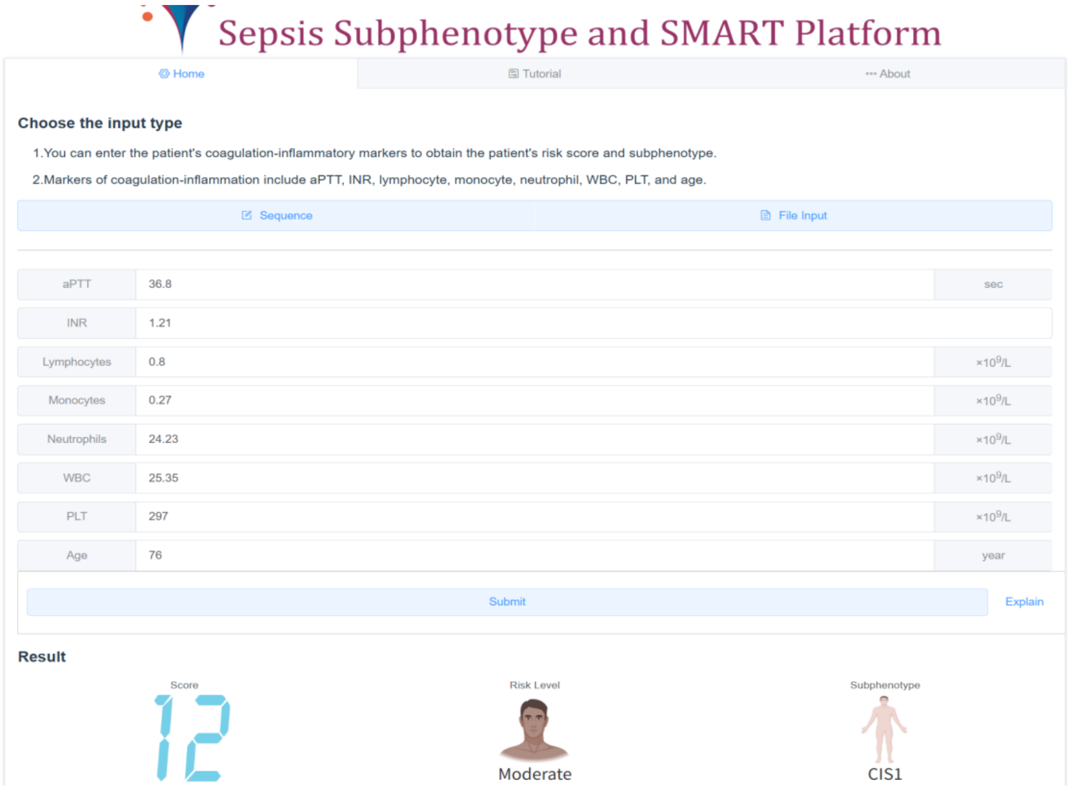

Case16.

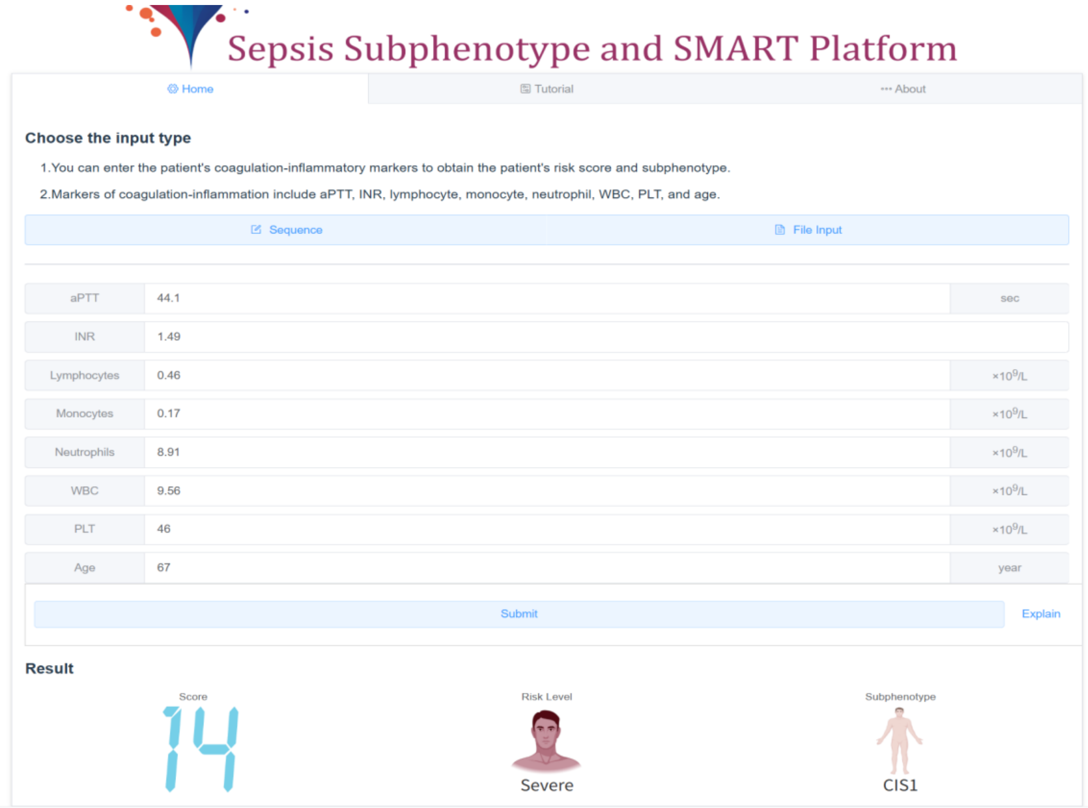

Case17.

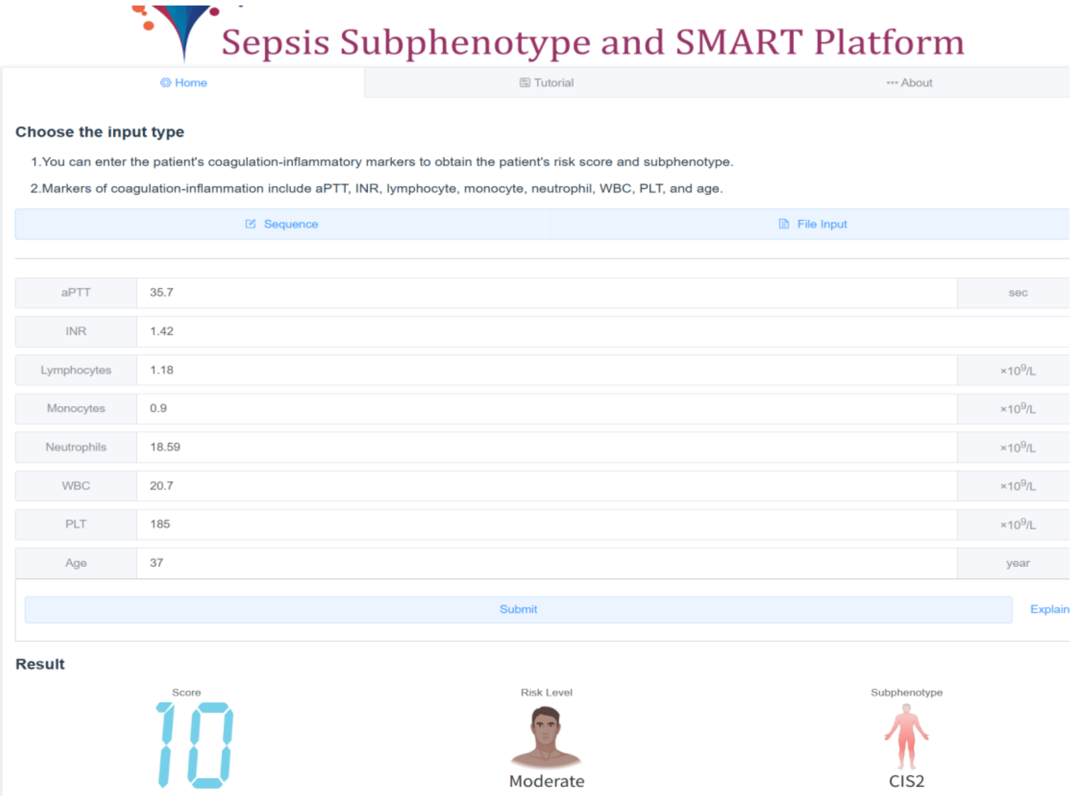

Case18.

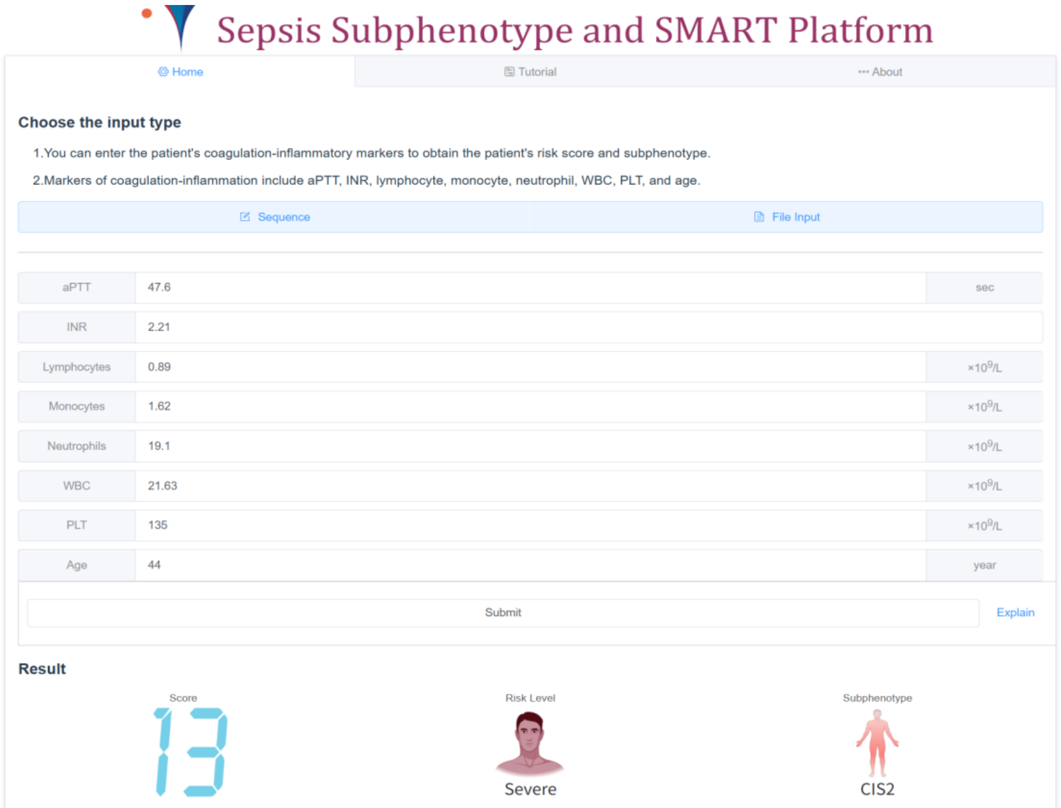

Case19.

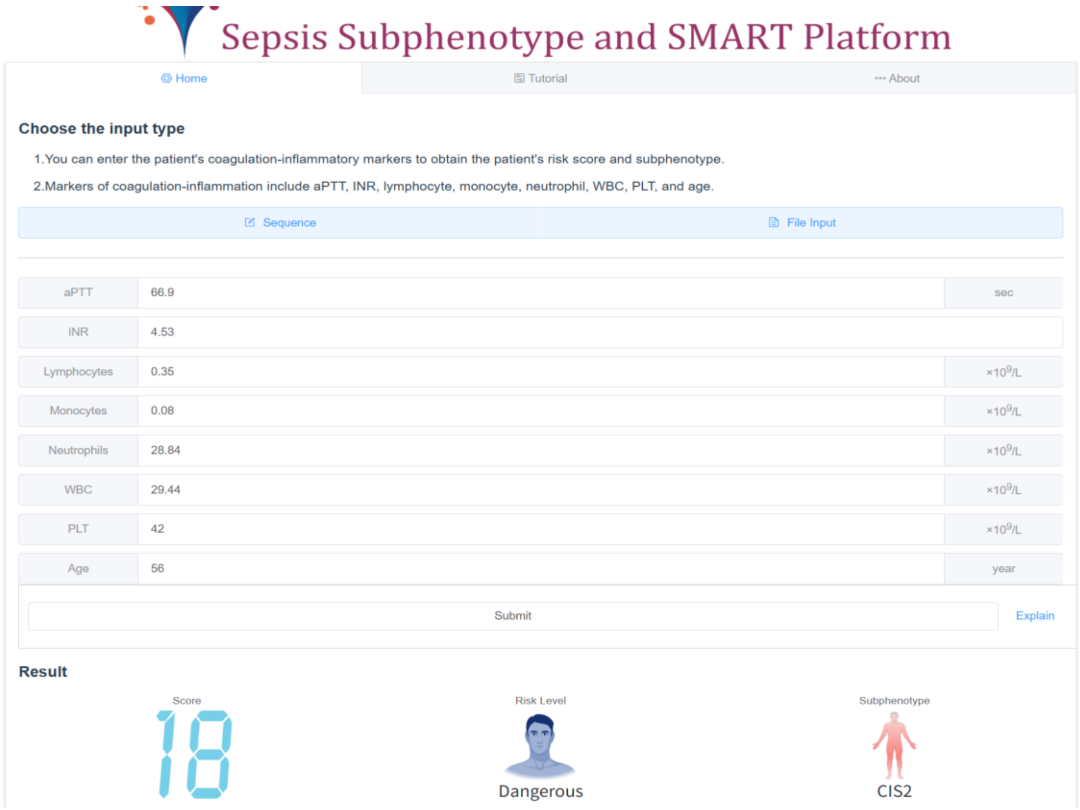

Case20.

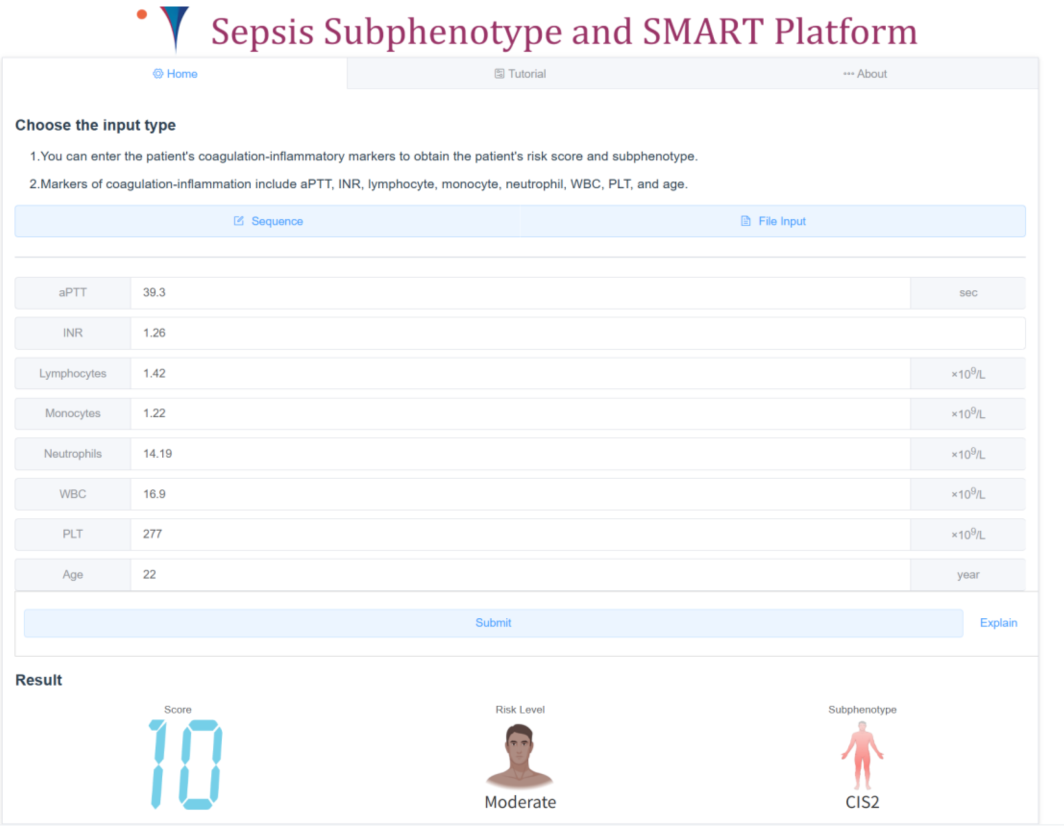

Case21.

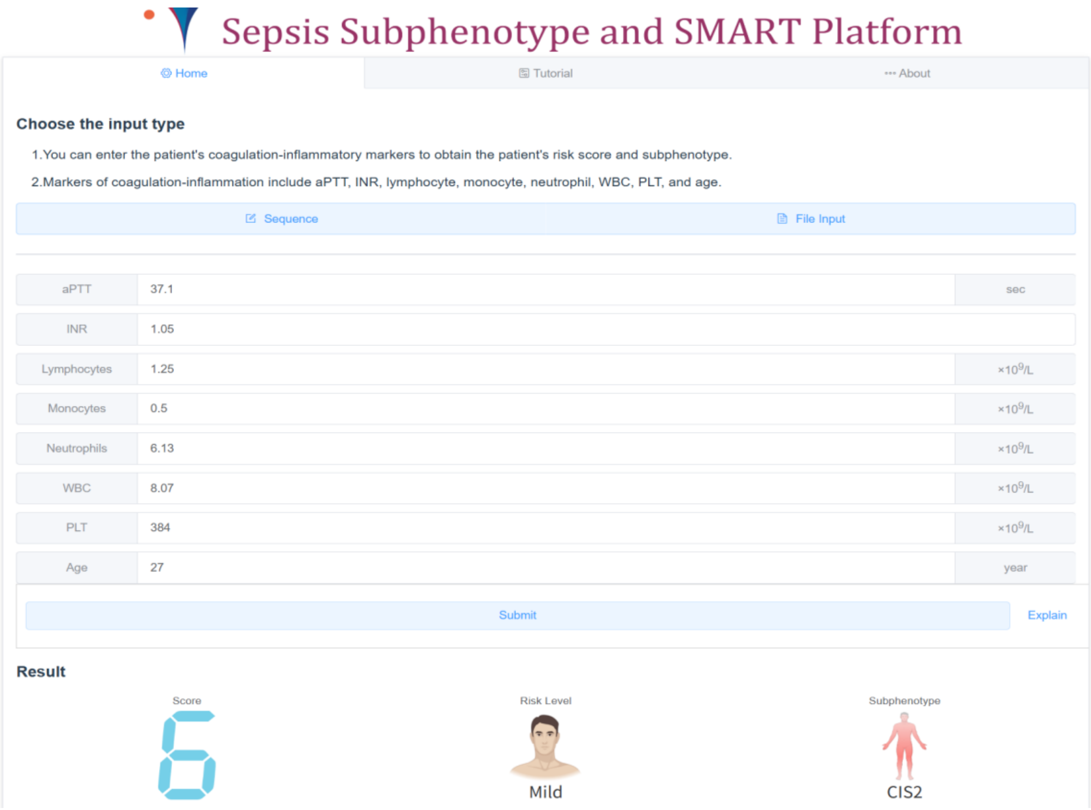

Case22.

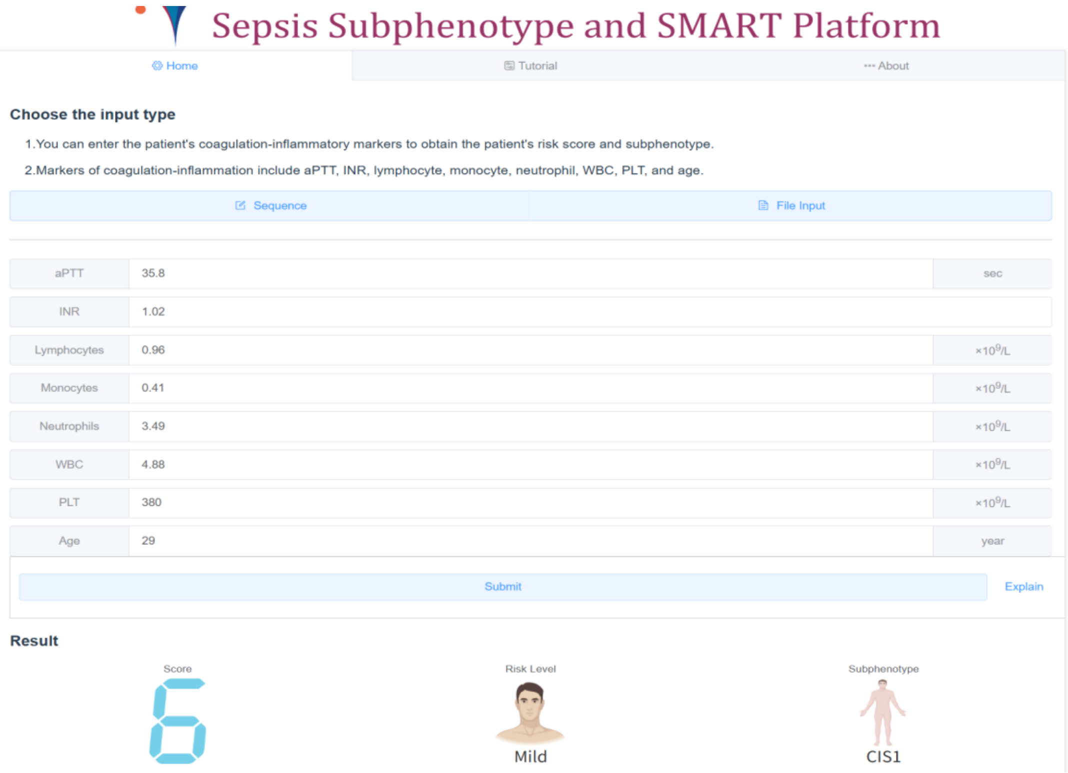

Case23.

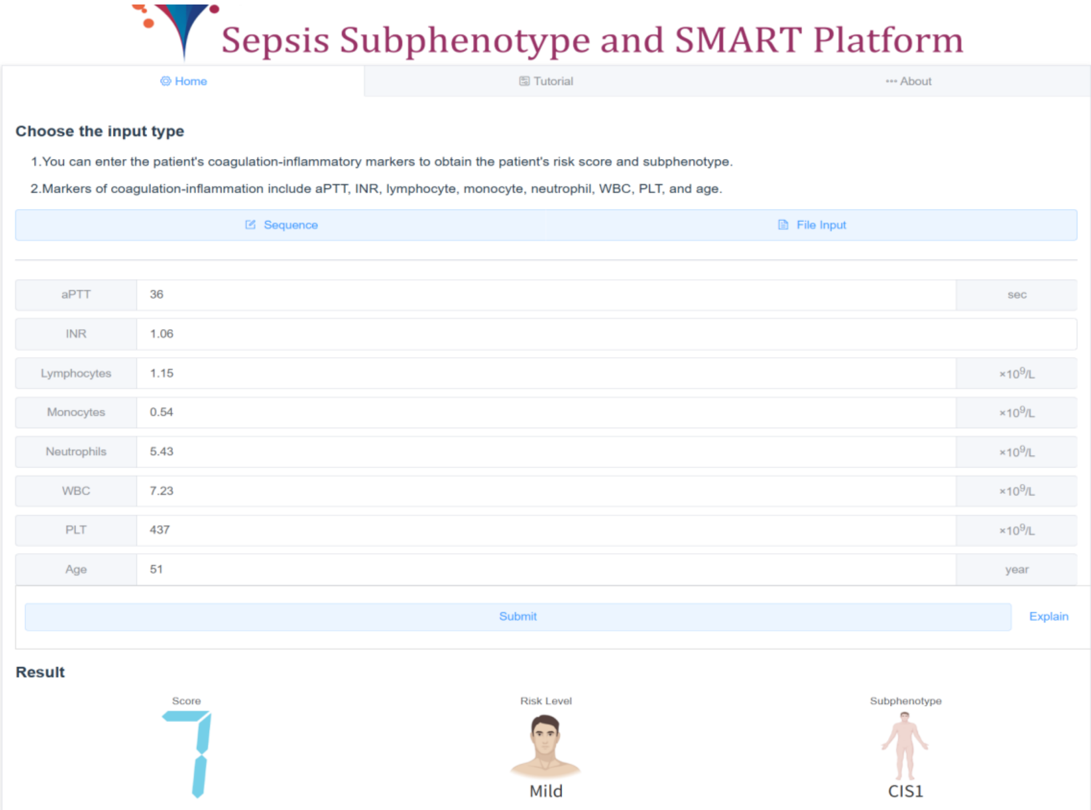

Case24.

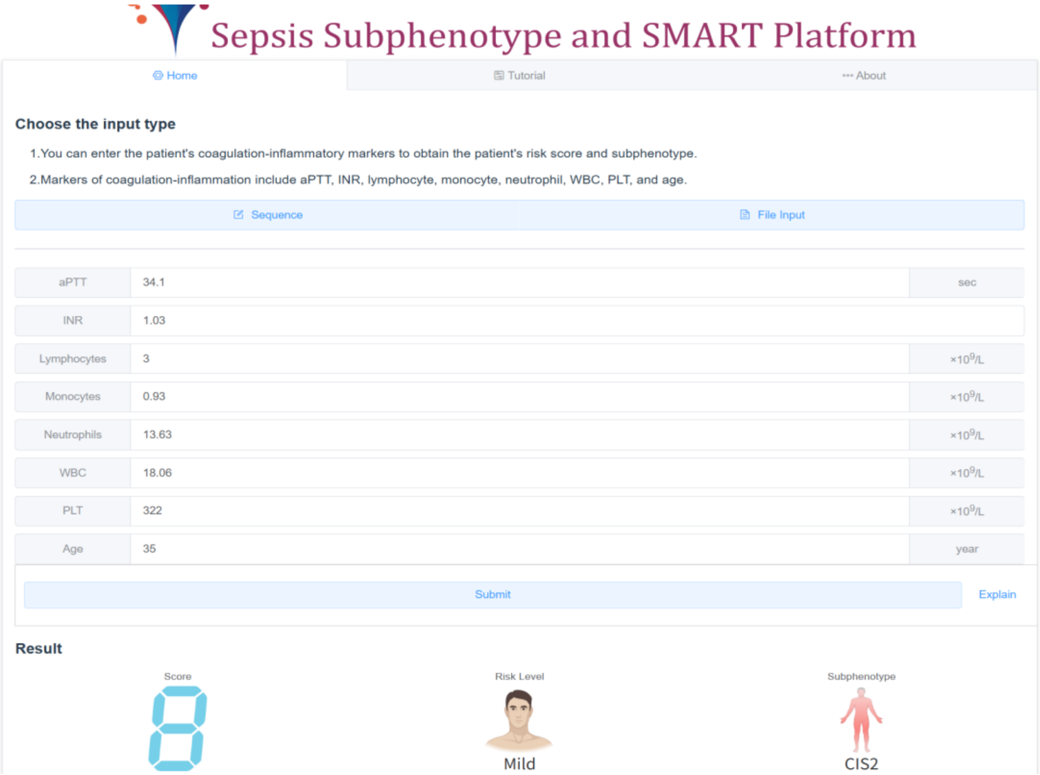

Case25.

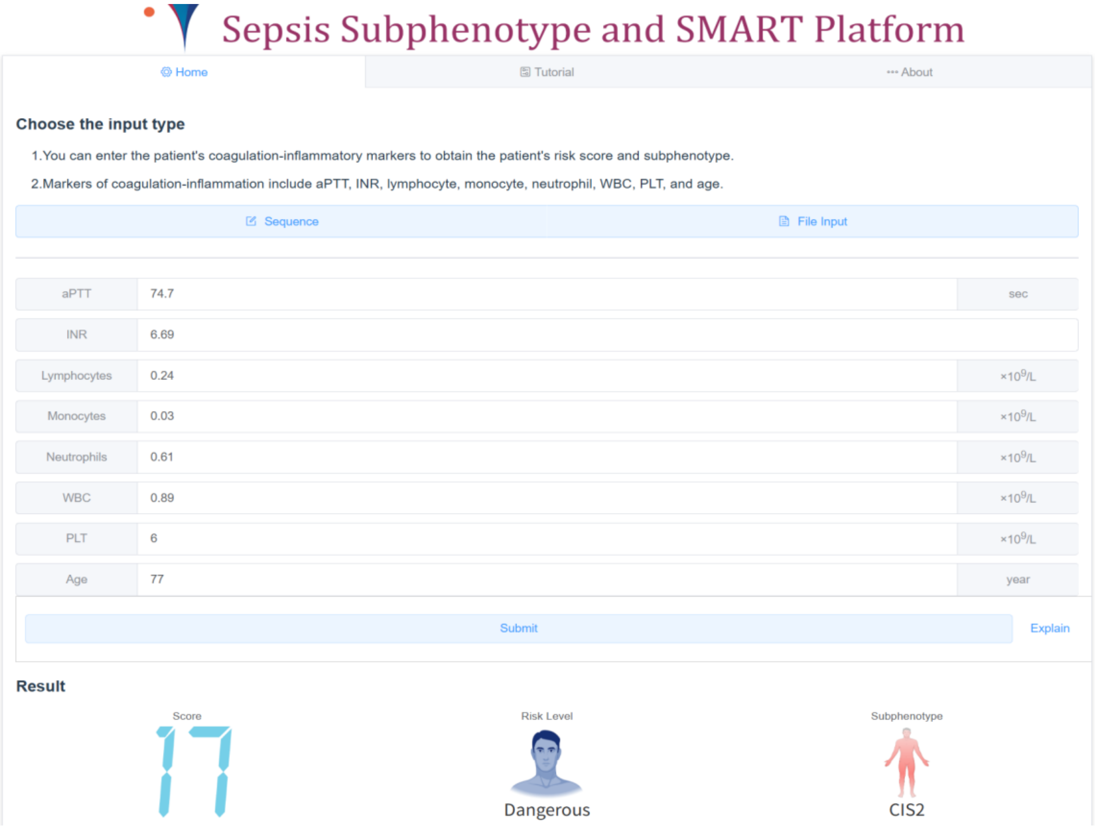

Case26.

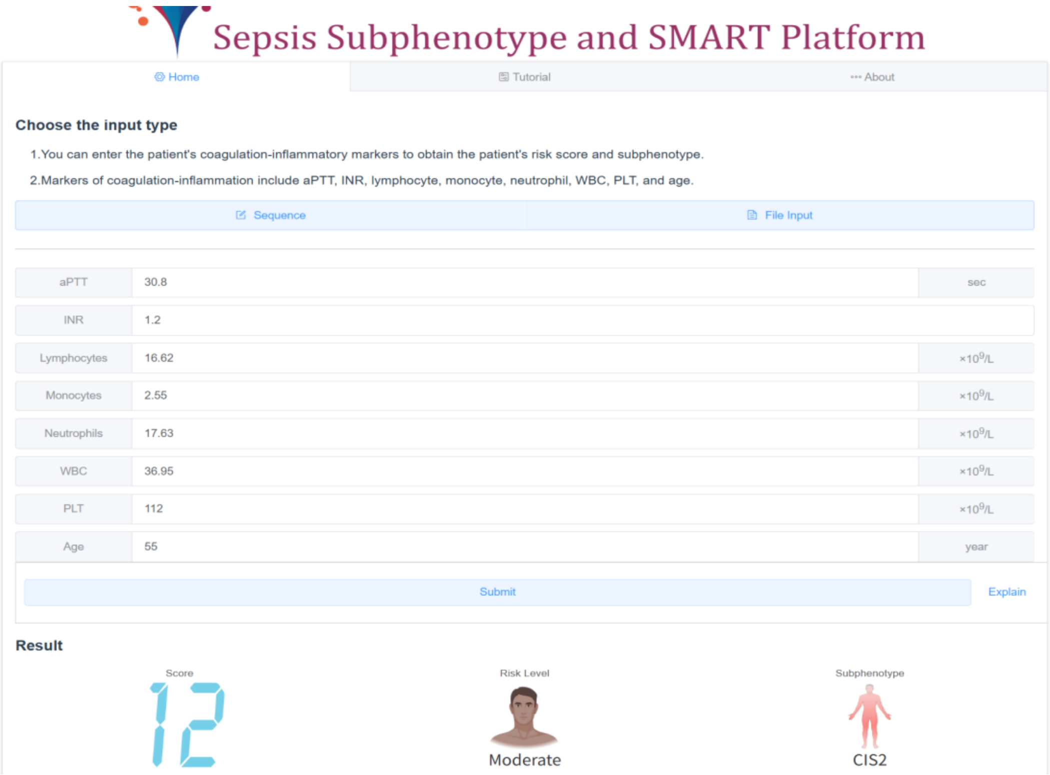

Case27.

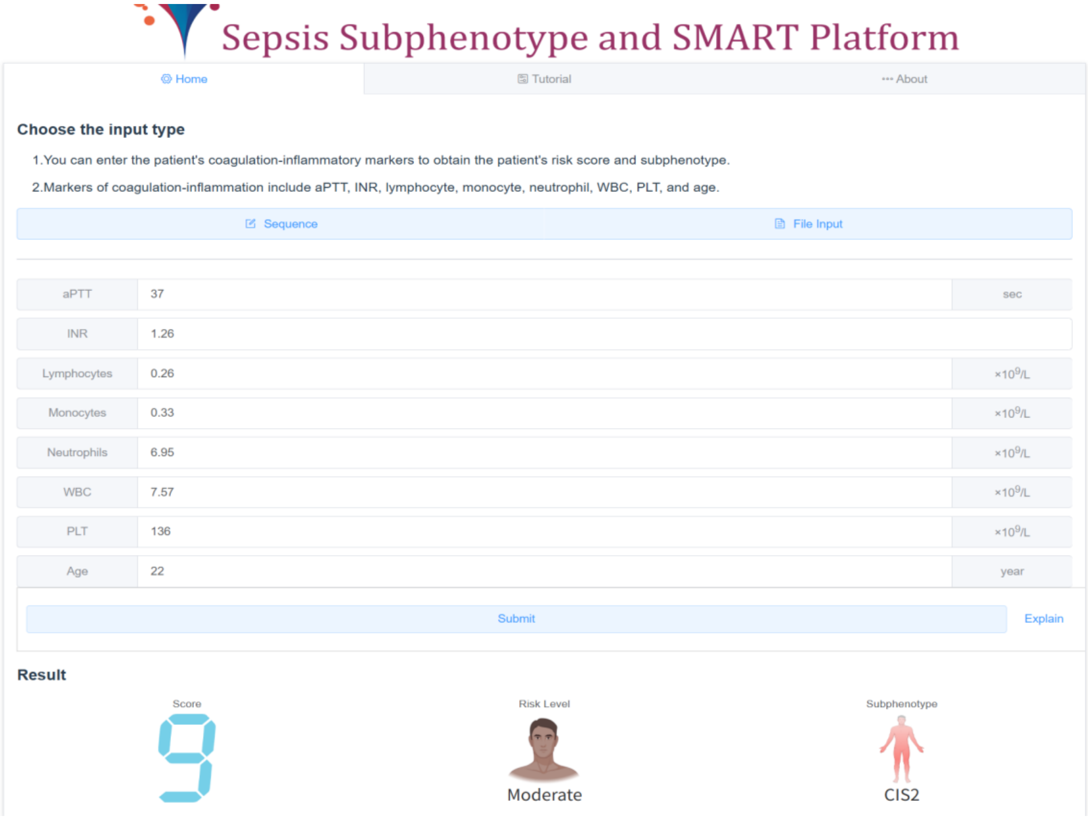

Case28.

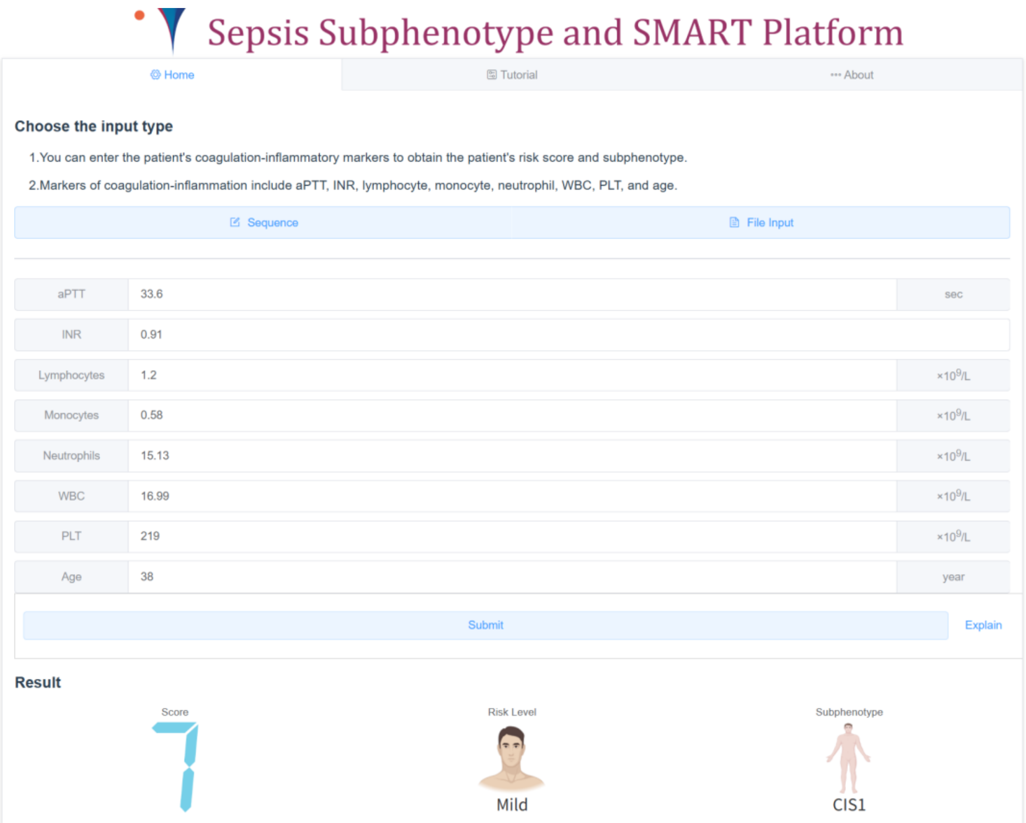

Case29.

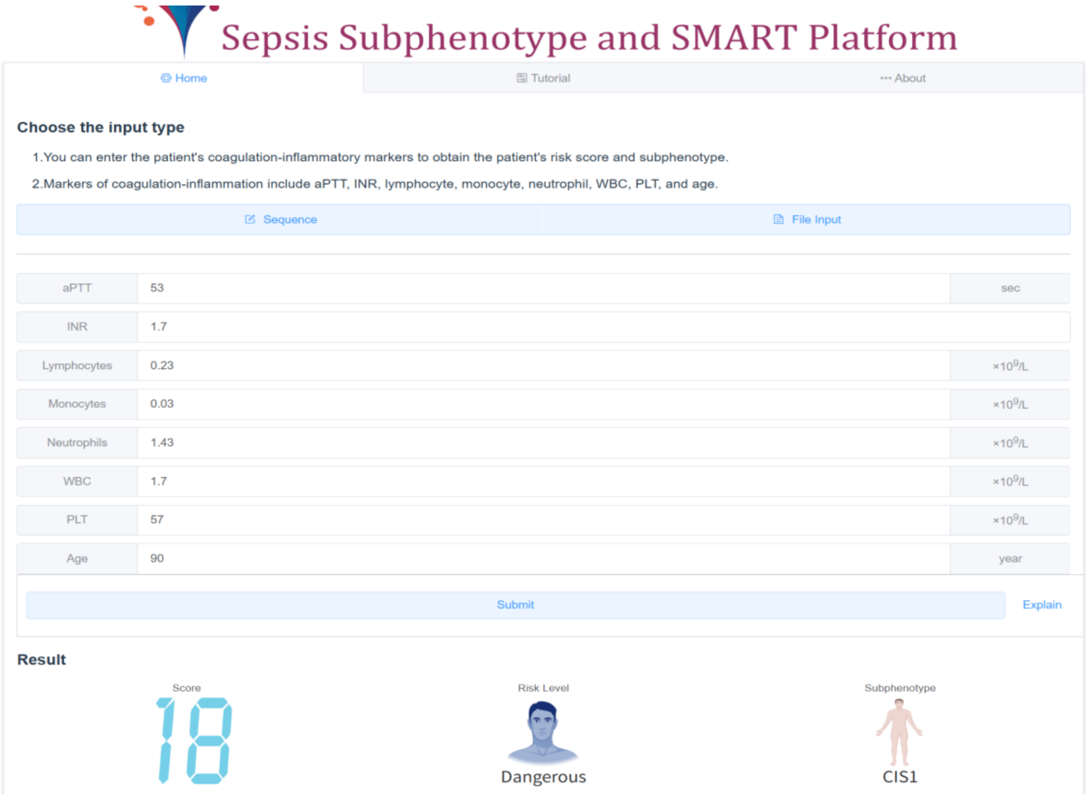

Case30.

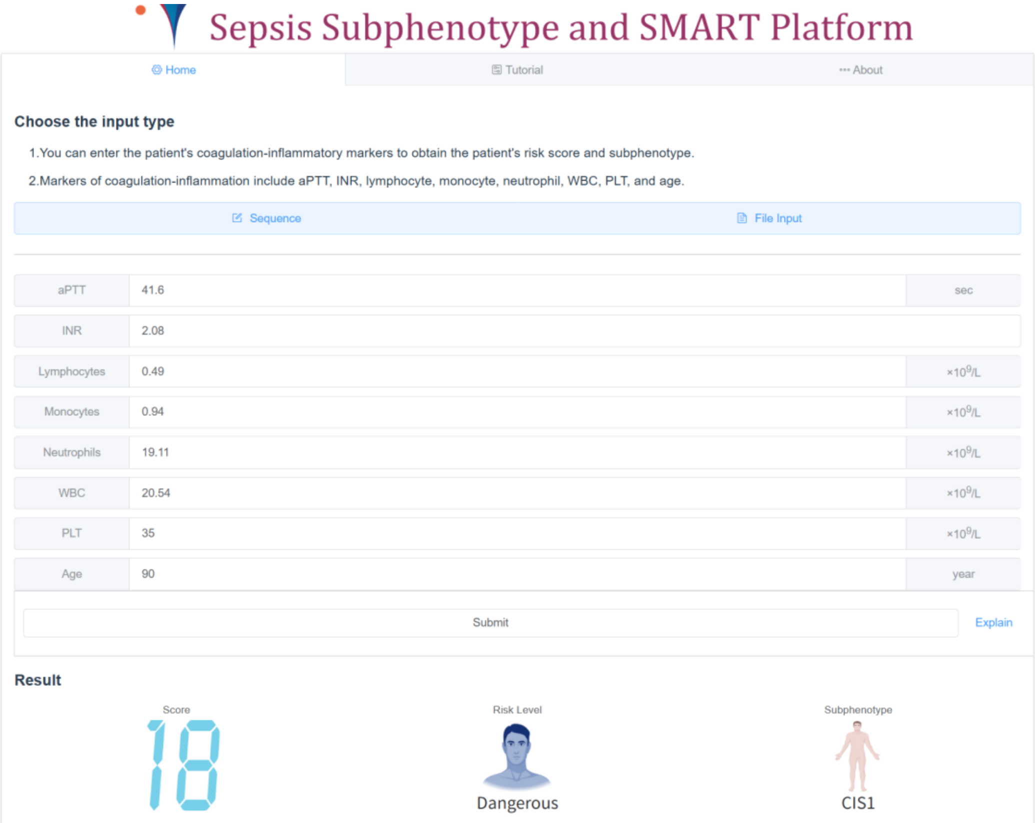

Case31.

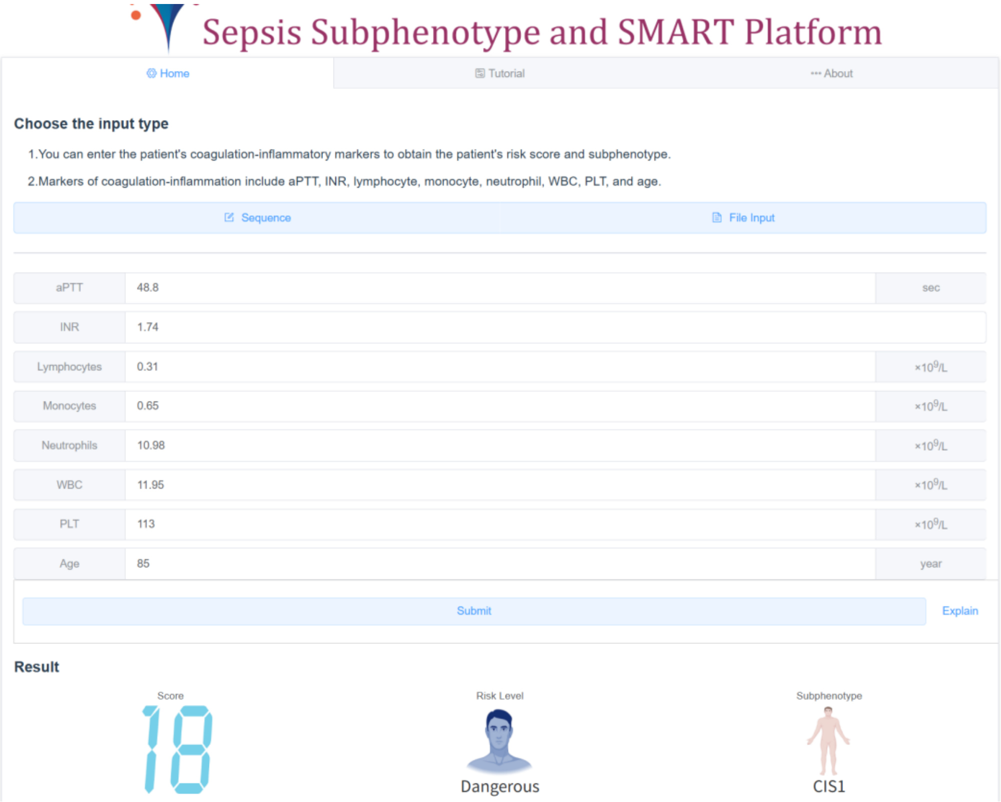

Case32.

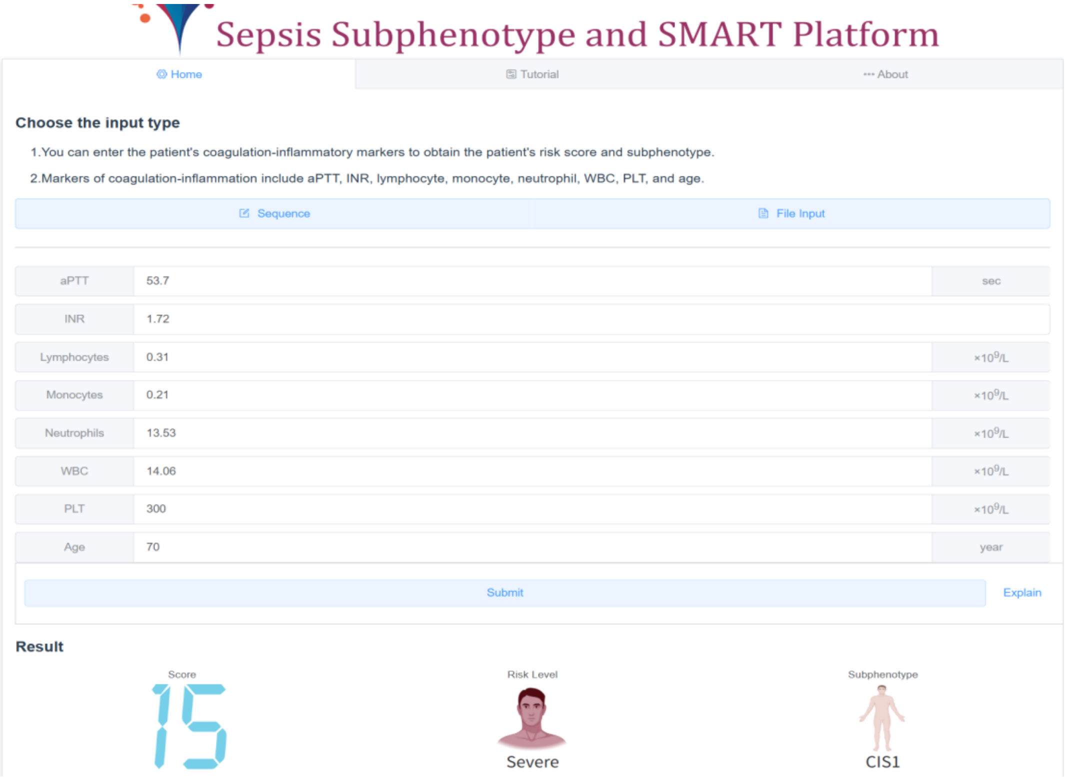

Case33.

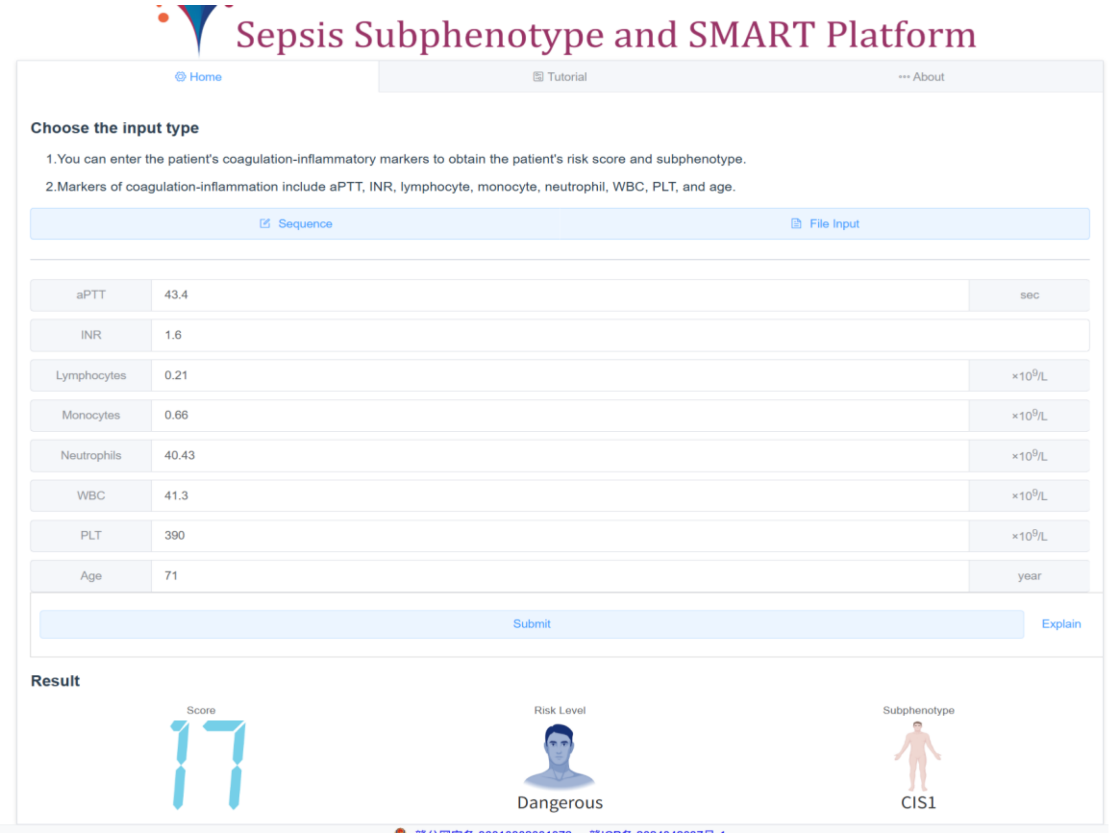

Case34.

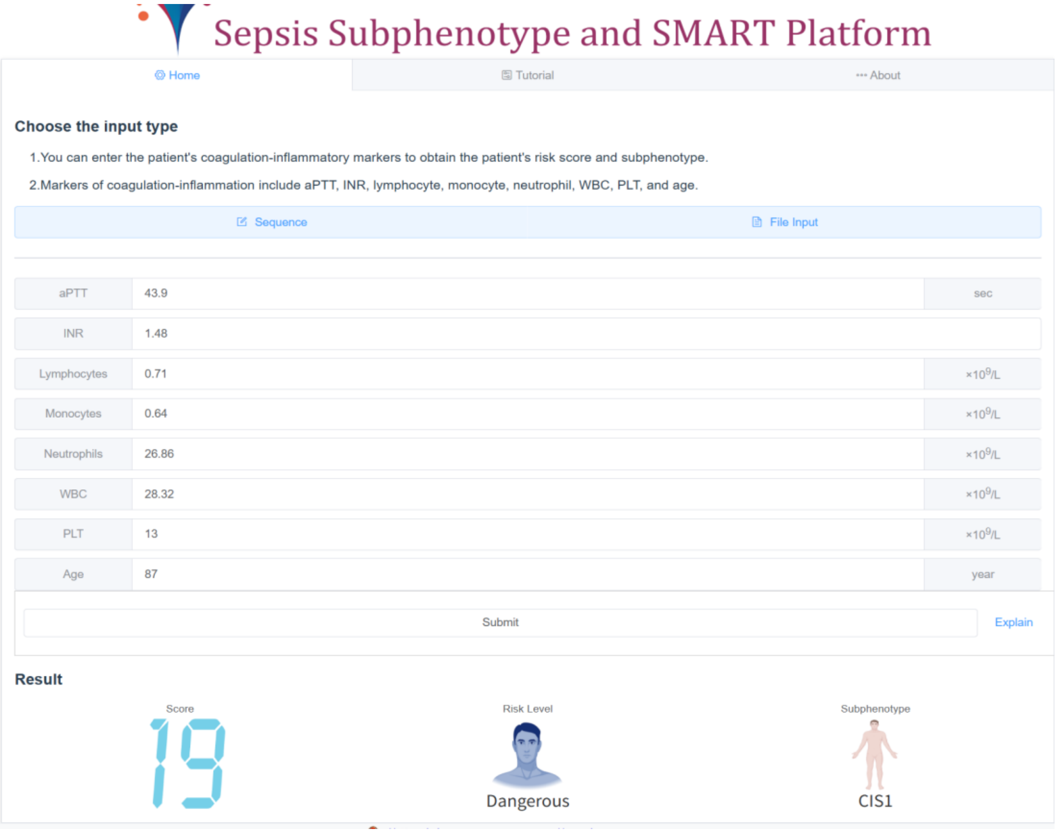

Case35.

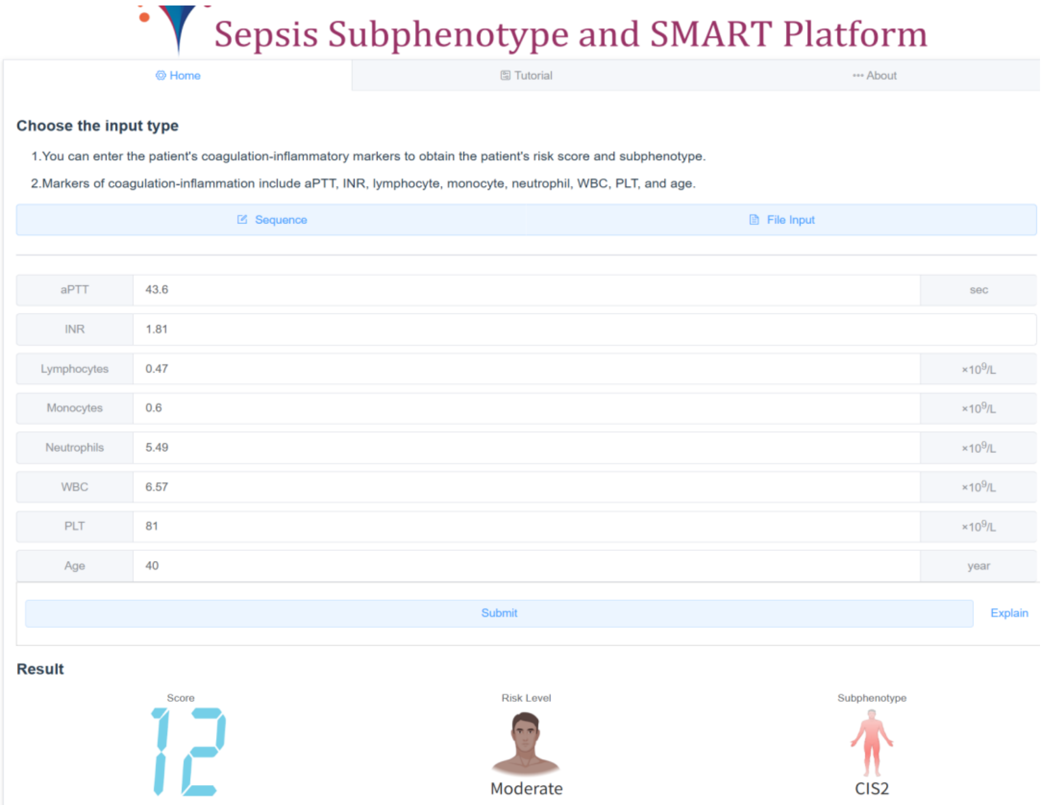

Case36.

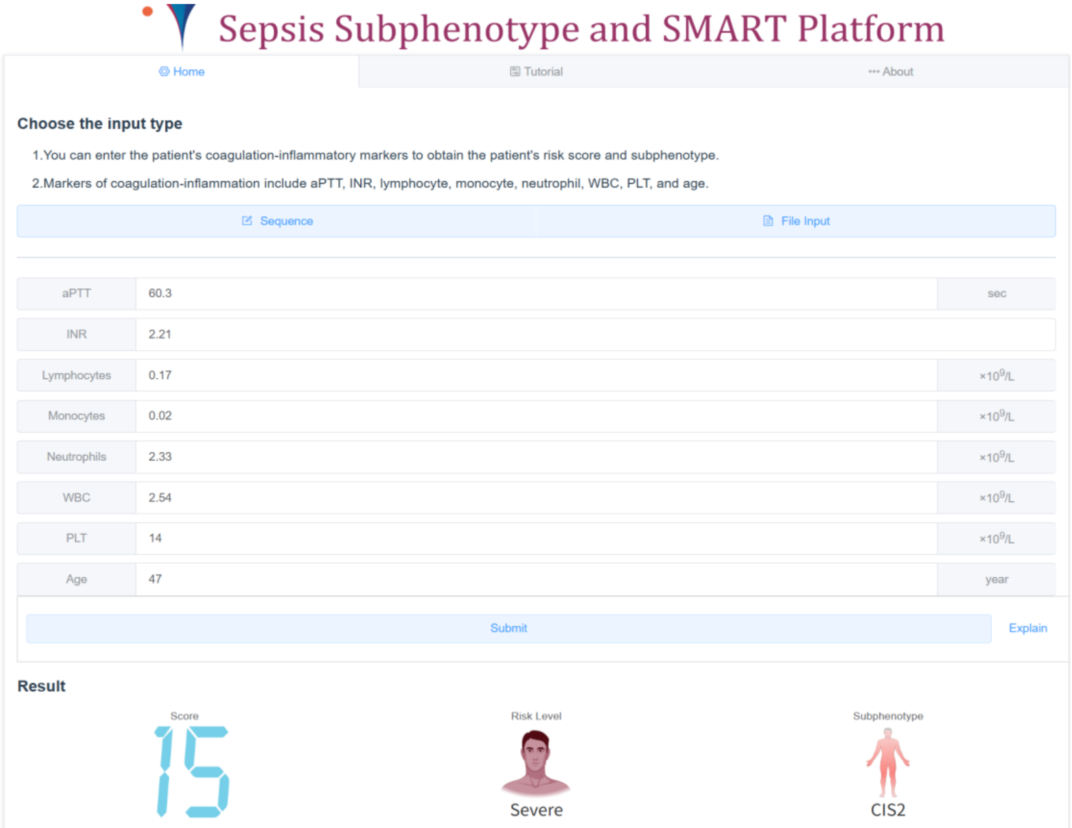

Case37.

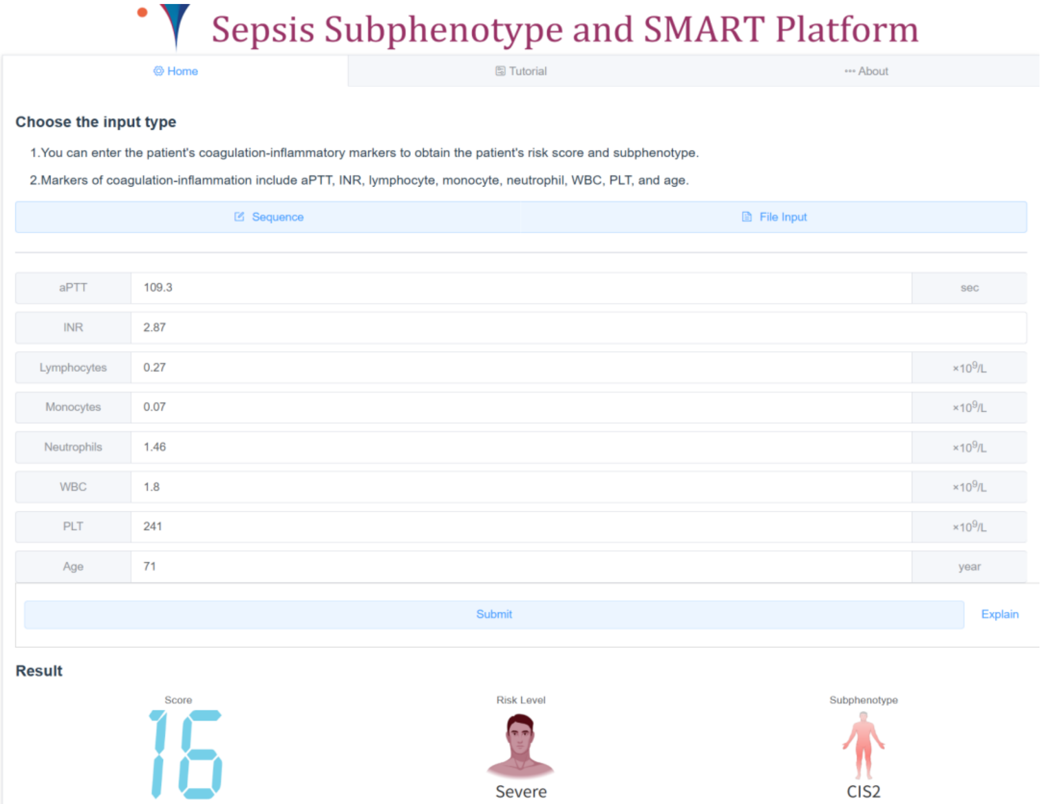

Case38.

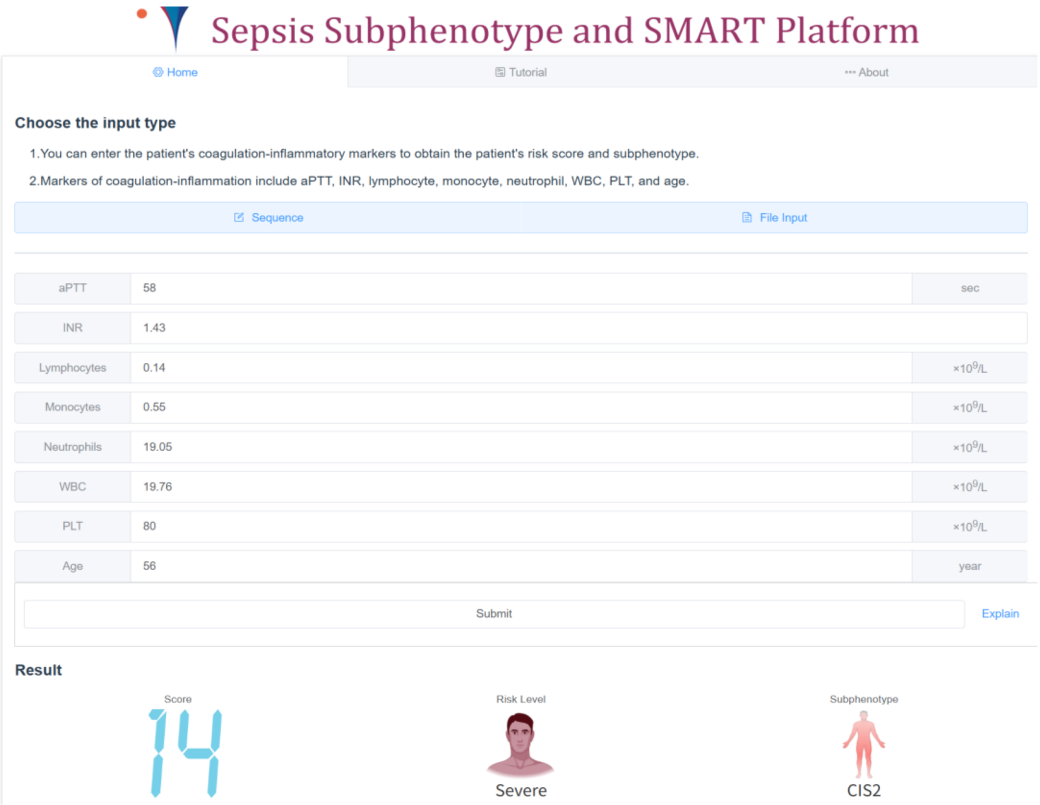

Case39.

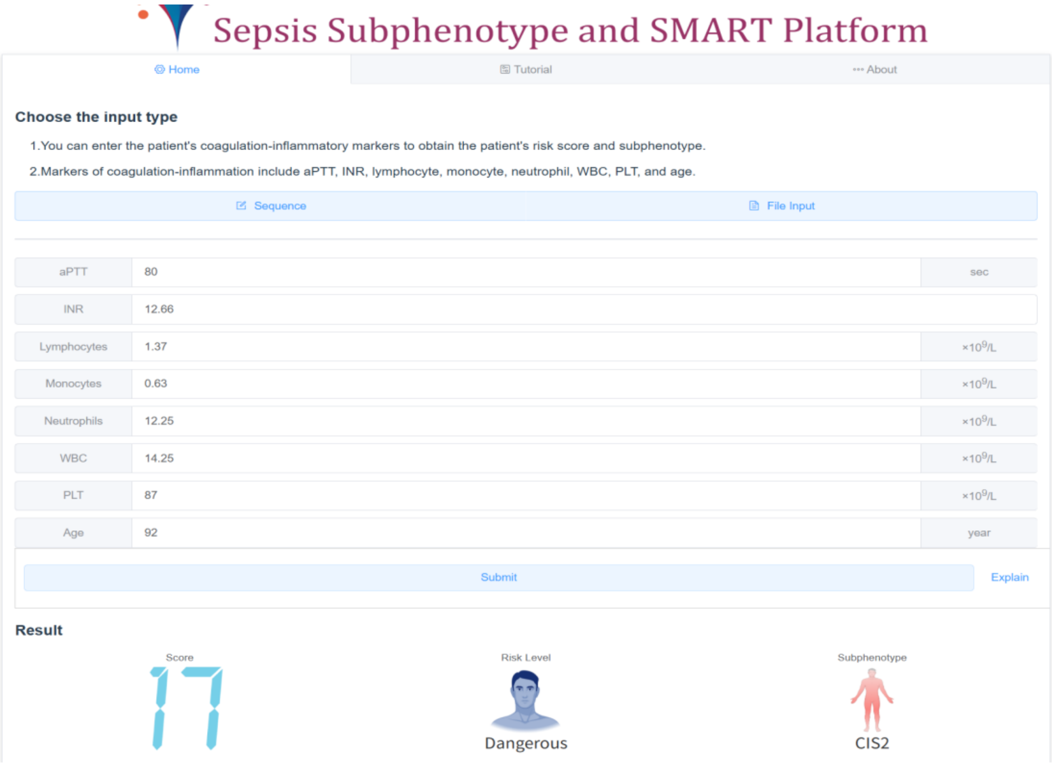

Case40.

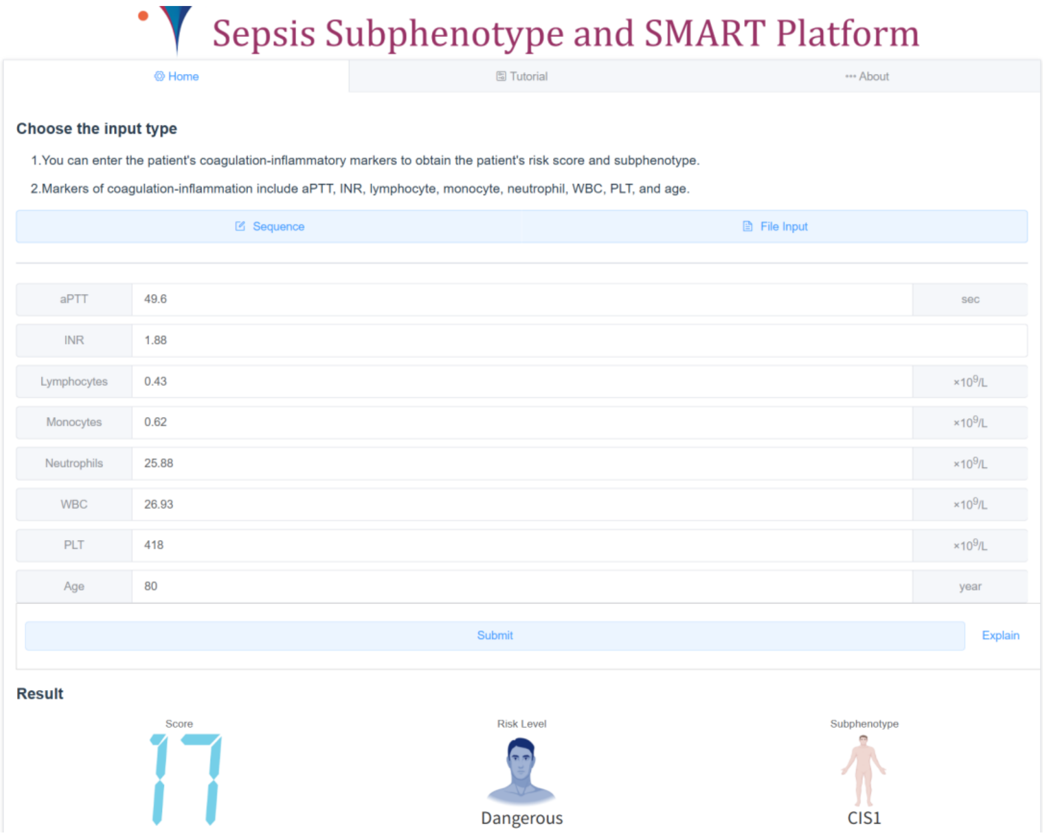

Sepsis Subphenotype and SMART Platform
Home
Tutorial
About
Choose the input type
1.You can enter the patient's coagulation-inflammatory markers to obtain the patient's risk score and subphenotype.
2.Markers of coagulation-inflammation include aPTT, INR, lymphocyte, monocyte, neutrophil, WBC, PLT, and age.
Sequence
File Input
aPTT 49.6 sec
INR 1.88
Lymphocytes 0.43 ×10⁹/L
Monocytes 0.62 ×10⁹/L
Neutrophils 25.88 ×10⁹/L
WBC 26.93 ×10⁹/L
PLT 418 ×10⁹/L
Age 80 year
Submit
Explain
Result
Score
17
Risk Level
Dangerous
Subphenotype
CIS1
