# Supplementary material for: Explainable AI unravels sepsis heterogeneity via coagulation-inflammation profiles for prognosis and stratification
Source: Nat Commun. 2025 Nov 24;16:10396. doi: 10.1038/s41467-025-65365-z (PMC12644763; doi:10.1038/s41467-025-65365-z)
Supplement: Supplementary file 2 — Reporting Summary [file 41467_2025_65365_MOESM2_ESM.pdf]

nature portfolio

Corresponding author(s): Yang Luo; Fei Guo; Bailin Niu

Last updated by author(s): Sep 6, 2025

Reporting Summary

Nature Portfolio wishes to improve the reproducibility of the work that we publish. This form provides structure for consistency and transparency in reporting. For further information on Nature Portfolio policies, see our Editorial Policies and the Editorial Policy Checklist.

Statistics

For all statistical analyses, confirm that the following items are present in the figure legend, table legend, main text, or Methods section.

n/a	Confirmed	
[ ]	[x]	The exact sample size (*n*) for each experimental group/condition, given as a discrete number and unit of measurement
[x]	[ ]	A statement on whether measurements were taken from distinct samples or whether the same sample was measured repeatedly
[ ]	[x]	The statistical test(s) used AND whether they are one- or two-sided<br>*Only common tests should be described solely by name; describe more complex techniques in the Methods section.*
[ ]	[x]	A description of all covariates tested
[ ]	[x]	A description of any assumptions or corrections, such as tests of normality and adjustment for multiple comparisons
[x]	[ ]	A full description of the statistical parameters including central tendency (e.g. means) or other basic estimates (e.g. regression coefficient) AND variation (e.g. standard deviation) or associated estimates of uncertainty (e.g. confidence intervals)
[ ]	[x]	For null hypothesis testing, the test statistic (e.g. *F*, *t*, *r*) with confidence intervals, effect sizes, degrees of freedom and *P* value noted<br>*Give P values as exact values whenever suitable.*
[x]	[ ]	For Bayesian analysis, information on the choice of priors and Markov chain Monte Carlo settings
[ ]	[x]	For hierarchical and complex designs, identification of the appropriate level for tests and full reporting of outcomes
[ ]	[x]	Estimates of effect sizes (e.g. Cohen's *d*, Pearson's *r*), indicating how they were calculated

Our web collection on statistics for biologists contains articles on many of the points above.

Software and code

Policy information about availability of computer code

Data collection	*Provide a description of all commercial, open source and custom code used to collect the data in this study, specifying the version used OR state that no software was used.*
Data analysis	Statistical analyses were performed via Python 3.8 and R 4.3.2. ROC curves were constructed to assess the predictive power of the SepsisFormer, SMART, and transcriptomic diagnostic and prognostic prediction models. Additionally, the performance evaluation of the transcriptomics prognostic prediction models included calibration curves, decision curves, and kappa consistency coefficient analyses. The effect of heparin treatment was evaluated via Kaplan–Meier plots for 28-day mortality and the Cox proportional hazards model. Kaplan–Meier plots were generated via GraphPad Prism 8.0 to examine the impact of three consecutive days of heparin treatment on survival outcomes in septic patients stratified by subphenotype and risk level. The Cox proportional hazards model was employed via HR to quantify the benefit of heparin across different subphenotypes and risk levels. Continuous variables are presented as medians (interquartile ranges) and were analyzed via non-parametric two-tailed Mann–Whitney U tests. Levene tests were used for variance homogeneity, and two-sided Wilcoxon rank-sum tests were used for violin plots. The log-rank test was applied to the Kaplan–Meier plots. A significance level of p<0.05 was considered statistically significant. DeLong's tests were performed to statistically compare the AUCs of different models using Delong_test from the MLstatkit.stats package, in conjunction with roc_auc_score from scikit-learn.

For manuscripts utilizing custom algorithms or software that are central to the research but not yet described in published literature, software must be made available to editors and reviewers. We strongly encourage code deposition in a community repository (e.g. GitHub). See the Nature Portfolio guidelines for submitting code & software for further information.

Data

Policy information about availability of data

All manuscripts must include a data availability statement. This statement should provide the following information, where applicable:

- Accession codes, unique identifiers, or web links for publicly available datasets
- A description of any restrictions on data availability
- For clinical datasets or third party data, please ensure that the statement adheres to our policy

Source data are provided in this paper. In this study, EHR data were obtained from the First Affiliated Hospital of Chongqing Medical University and three publicly available databases: MIMIC-III(https://physionet.org/content/mimiciii/1.4/), MIMIC-IV(https://physionet.org/content/mimiciv/2.2/), and eICU-CRD (https://www.physionet.org/content/eicu-crd/2.0/). For the public EHR data, we adhered to all data use agreements, conducting experiments on observational, retrospective data. All three datasets require user registration and a signed data use agreement for timely access.Transcriptomic data were obtained from the Ningbo Medical Center Lihuili Hospital and four publicly available datasets: the Gene Expression Omnibus (GEO, https://www.ncbi.nlm.nih.gov/geo/), the Kyoto Encyclopedia of Genes and Genomes (KEGG, https://www.genome.jp/kegg/), GeneCards (https://www.genecards.org/), and DisGeNET (https://www.disgenet.org/). To promote transparency, reproducibility, and clinical applicability, we have made the following resources publicly available.

Research involving human participants, their data, or biological material

Policy information about studies with human participants or human data. See also policy information about sex, gender (identity/presentation), and sexual orientation and race, ethnicity and racism.

Reporting on sex and gender	In the design and implementation of this study, there is no sex or gender difference and no sex or gender bias.
Reporting on race, ethnicity, or other socially relevant groupings	There is no race, ethnicity, or other socially relevant groupings difference.
Population characteristics	This study utilizes advanced AI technology to open up a risk stratification and subphenotypic classification tool for sepsis. Patient data from different databases were divided into the training set and the validation set. There is no comparison of gender differences and basic characteristics of the population involved here, but it is presented in Supplementary Information Table 2.
Recruitment	We were a retrospective study, directly enrolling patients based on the inclusion and exclusion criteria.
Ethics oversight	As this study involved multi-center and multi-omics data, it received approval from the Ethics Committee of the First Affiliated Hospital of Chongqing Medical University (NO. 2019-312), the Ethics Committee of Chongqing University Affiliated Central Hospital (NO. 2025-55), and the Ethics Committee of Ningbo Medical Center Lihuili Hospital (NO. KY2023SL146-01). Permission to use the data was obtained for all the databases (MIMIC-III No. 36181465, MIMIC-IV No. 46463103, and eICU-CRD No. 12855636),with the permission of the relevant databases, the informed consent form and ethical approval were waived.

Note that full information on the approval of the study protocol must also be provided in the manuscript.

Field-specific reporting

Please select the one below that is the best fit for your research. If you are not sure, read the appropriate sections before making your selection.

[x] Life sciences [ ] Behavioural & social sciences [ ] Ecological, evolutionary & environmental sciences

For a reference copy of the document with all sections, see nature.com/documents/nr-reporting-summary-flat.pdf

Life sciences study design

All studies must disclose on these points even when the disclosure is negative.

Sample size	A total of 428 septic patients admitted to the First Affiliated Hospital of Chongqing Medical University between January 2018 and April 2021 were enrolled. 11,980 septic patients from publicly available databases were included (MIMIC-III: 2,371; MIMIC-IV: 4,191; eICU-CRD: 5,418). Blood samples for RT-qPCR were collected from 29 septic patients and 11 healthy volunteers at Ningbo Medical Center Lihuili Hospital. The GSE65682 dataset from the GEO database comprises 760 sepsis patient samples and 42 healthy control samples. For real-time and external validation, 40 patients with sepsis were enrolled at Chongqing Emergency Medical Center between March 24, 2025, and April 28, 2025.
Data exclusions	Patients were enrolled within one hour of admission or within one hour of an acute exacerbation for current inpatients, following the Sepsis-3 definition; and patients were excluded with incomplete blood test results and those under 18 years of age.
Replication	N/A
Randomization	N/A
Blinding	N/A

Reporting for specific materials, systems and methods

We require information from authors about some types of materials, experimental systems and methods used in many studies. Here, indicate whether each material, system or method listed is relevant to your study. If you are not sure if a list item applies to your research, read the appropriate section before selecting a response.

Materials & experimental systems

n/a	Involved in the study
x	☐ Antibodies
x	☐ Eukaryotic cell lines
x	☐ Palaeontology and archaeology
x	☐ Animals and other organisms
x	☐ Clinical data
x	☐ Dual use research of concern
x	☐ Plants

Methods

n/a	Involved in the study
x	☐ ChIP-seq
x	☐ Flow cytometry
x	☐ MRI-based neuroimaging

Plants

Seed stocks	N/A
Novel plant genotypes	N/A
Authentication	N/A
